# Short-course primaquine for the radical cure of *Plasmodium vivax* malaria: a multicentre, randomised, placebo-controlled non-inferiority trial

**DOI:** 10.1016/S0140-6736(19)31285-1

**Published:** 2019-09-14

**Authors:** Walter R J Taylor, Kamala Thriemer, Lorenz von Seidlein, Prayoon Yuentrakul, Thanawat Assawariyathipat, Ashenafi Assefa, Sarah Auburn, Krisin Chand, Nguyen Hoang Chau, Phaik Yeong Cheah, Le Thanh Dong, Mehul Dhorda, Tamiru Shibru Degaga, Angela Devine, Lenny L Ekawati, Fahmi Fahmi, Asrat Hailu, Mohammad Anwar Hasanzai, Tran Tinh Hien, Htee Khu, Benedikt Ley, Yoel Lubell, Jutta Marfurt, Hussein Mohammad, Kerryn A Moore, Mohammad Nader Naddim, Ayodhia Pitaloka Pasaribu, Syahril Pasaribu, Cholrawee Promnarate, Awab Ghulam Rahim, Pasathron Sirithiranont, Hiwot Solomon, Herawati Sudoyo, Inge Sutanto, Ngo Viet Thanh, Nguyen Thi Tuyet-Trinh, Naomi Waithira, Adugna Woyessa, Fazal Yamin Yamin, Arjen Dondorp, Julie A Simpson, J Kevin Baird, Nicholas J White, Nicholas P Day, Ric N Price

**Affiliations:** a Mahidol-Oxford Tropical Medicine Research Unit, Faculty of Tropical Medicine, Mahidol University, Bangkok, Thailand; b Global and Tropical Health Division, Menzies School of Health Research and Charles Darwin University, Darwin, NT, Australia; c Centre for Tropical Medicine and Global Health, Nuffield Department of Medicine, University of Oxford, Oxford, UK; d Ethiopian Public Health Institute, Addis Ababa, Ethiopia; e Eijkman-Oxford Clinical Research Unit, Eijkman Institute of Molecular Biology, Jakarta, Indonesia; f Oxford University Clinical Research Unit, Hospital for Tropical Diseases, Ho Chi Minh City, Vietnam; g Institute of Malariology, Parasitology and Entomology, Ho Chi Minh City, Vietnam; h Worldwide Antimalarial Resistance Network, Asia Regional Centre, Faculty of Tropical Medicine, Mahidol University, Bangkok, Thailand; i College of Medicine & Health Sciences, Arbaminch University, Arbaminch, Ethiopia; j Centre for Epidemiology and Biostatistics, Melbourne School of Population and Global Health, University of Melbourne, Melbourne, VIC, Australia; k Universitas Sumatera Utara, Medan, Indonesia; l College of Health Sciences, Addis Ababa University, Addis Ababa, Ethiopia; m Health Protection and Research Organisation, Kabul, Afghanistan; n Maternal and Child Health Program, Life Sciences and Public Health, Burnet Institute, Melbourne, VIC, Australia; o Nangarhar Medical Faculty, Nangarhar University, Ministry of Higher Education, Jalalabad, Afghanistan; p Federal Ministry of Health, Addis Ababa, Ethiopia; q Eijkman Institute for Molecular Biology, Jakarta, Indonesia; r Faculty of Medicine, Universitas Indonesia, Jakarta, Indonesia; s Krong-Pa Health Centre, Krong-Pa, Vietnam; t Health and Social Development Organization, Kabul, Afghanistan

## Abstract

**Background:**

Primaquine is the only widely used drug that prevents *Plasmodium vivax* malaria relapses, but adherence to the standard 14-day regimen is poor. We aimed to assess the efficacy of a shorter course (7 days) of primaquine for radical cure of vivax malaria.

**Methods:**

We did a randomised, double-blind, placebo-controlled, non-inferiority trial in eight health-care clinics (two each in Afghanistan, Ethiopia, Indonesia, and Vietnam). Patients (aged ≥6 months) with normal glucose-6-phosphate dehydrogenase (G6PD) and presenting with uncomplicated vivax malaria were enrolled. Patients were given standard blood schizontocidal treatment and randomly assigned (2:2:1) to receive 7 days of supervised primaquine (1·0 mg/kg per day), 14 days of supervised primaquine (0·5 mg/kg per day), or placebo. The primary endpoint was the incidence rate of symptomatic *P vivax* parasitaemia during the 12-month follow-up period, assessed in the intention-to-treat population. A margin of 0·07 recurrences per person-year was used to establish non-inferiority of the 7-day regimen compared with the 14-day regimen. This trial is registered at ClinicalTrials.gov (NCT01814683).

**Findings:**

Between July 20, 2014, and Nov 25, 2017, 2336 patients were enrolled. The incidence rate of symptomatic recurrent *P vivax* malaria was 0·18 (95% CI 0·15 to 0·21) recurrences per person-year for 935 patients in the 7-day primaquine group and 0·16 (0·13 to 0·18) for 937 patients in the 14-day primaquine group, a difference of 0·02 (−0·02 to 0·05, p=0·3405). The incidence rate for 464 patients in the placebo group was 0·96 (95% CI 0·83 to 1·08) recurrences per person-year. Potentially drug-related serious adverse events within 42 days of starting treatment were reported in nine (1·0%) of 935 patients in the 7-day group, one (0·1%) of 937 in the 14-day group and none of 464 in the control arm. Four of the serious adverse events were significant haemolysis (three in the 7-day group and one in the 14-day group).

**Interpretation:**

In patients with normal G6PD, 7-day primaquine was well tolerated and non-inferior to 14-day primaquine. The short-course regimen might improve adherence and therefore the effectiveness of primaquine for radical cure of *P vivax* malaria.

**Funding:**

UK Department for International Development, UK Medical Research Council, UK National Institute for Health Research, and the Wellcome Trust through the Joint Global Health Trials Scheme (MR/K007424/1) and the Bill & Melinda Gates Foundation (OPP1054404).

## Introduction

*Plasmodium vivax* malaria is a major cause of morbidity and a contributor to mortality in Asia, Oceania, the Horn of Africa, and the Americas.^1^, ^2^ The infection is characterised by an acute febrile illness followed by recurrent febrile illnesses in the weeks to months after the initial infection.^3^ These malaria relapses arise from dormant liver stages (hypnozoites) that are refractory to most antimalarial drugs.^4^, ^5^, ^6^ Relapse is the main cause of vivax illness in endemic areas.^6^, ^7^ Primaquine, an 8-aminoquinoline, is the only widely available drug that kills hypnozoites and prevents relapses,^8^ but its use is limited by the risk of acute haemolysis in patients with glucose-6-phosphate dehydrogenase (G6PD) deficiency. When supervised, a 14-day regimen of primaquine effectively prevents *P vivax* relapses.^9^, ^10^, ^11^ However, in most malaria-endemic settings, daily supervision of a prolonged treatment regimen is not practical; an unsupervised regimen is associated with a significantly reduced primaquine adherence and effectiveness compared with a supervised regimen.^12^, ^13^, ^14^

**Research in context**

**Evidence before this study**

The primary determinant of primaquine radical cure efficacy is the total dose of primaquine administered. In areas with frequent-relapsing strains of *Plasmodium vivax*, WHO recommends a high-dose primaquine regimen of 7 mg/kg administered over 14 days (0·5 mg/kg per day, equivalent to an adult dose of 30 mg per day). In 2012, at the inception of the study, a systematic review was done using the search terms “vivax” and “primaquine” to identify all articles in the English language on PubMed, MEDLINE, Web of Science, Embase, and the Cochrane Database of Systematic Reviews between Jan 1, 1950, and Dec 31, 2011. The search identified 18 studies that compared the efficacy of schizontocidal treatment with and without primaquine. However, wide variation in study design and duration of follow-up confounded comparison of antirelapse efficacy across different endemic settings. The efficacy of an unsupervised 14-day primaquine regimen was significantly lower than that of a daily observed 14-day regimen (odds ratio 0·18 [95% CI 0·06–0·57]). Only one study compared the efficacy of a high-dose primaquine regimen administered over 7 or 14 days, and although the results suggested equivalence, patients were only followed up for 28 days. A 2019 randomised controlled trial compared the antirelapse efficacy of high-dose primaquine administered over 7 or 14 days in Thai patients, and found non-inferiority at a 10% margin. However the generalisability of these findings across other endemic locations has yet to be confirmed.

**Added value of this study**

Our large multicentre clinical trial in patients with *P vivax* was done at eight sites in four countries with differing *P vivax* endemicity, to assess the safety and radical curative efficacy of a 7-day high-dose (1 mg/kg per day) primaquine regimen compared with a 14-day regimen (0·5 mg/kg per day). The study shows that, in patients screened with the qualitative fluorescent spot test as glucose-6-phosphate dehydrogenase normal, the 7-day primaquine regimen was non-inferior to the 14-day regimen, with an acceptable safety profile.

**Implications of all the available evidence**

In patients with normal G6PD, a 7-day high-dose course of primaquine is well tolerated and is as effective as a 14-day course. The shorter regimen has the potential to improve adherence and therefore the effectiveness of primaquine for the radical cure of *P vivax* malaria.

WHO recommends primaquine (0·5 mg/kg per day for 14 days) for patients with normal G6PD activity in areas where frequent relapsing strains of *P vixax* are prevalent.^15^ However, G6PD testing is usually unavailable and many countries recommend lower doses (0·25 mg/kg per day for 14 days) to reduce the risk of drug-induced haemolysis. In practice, primaquine is rarely used, even at this lower dose, mainly because of a fear of drug-induced haemolysis and poor awareness of the benefits of primaquine in preventing relapsing infections in high-transmission areas.^16^, ^17^ The primary determinant of the radical curative efficacy of primaquine is the total dose administered.^8^ Shorter courses with higher daily doses might improve adherence, and thus effectiveness, without reducing efficacy,^18^, ^19^ but concerns over safety in patients with unknown G6PD status have made policy makers reluctant to adopt this strategy. The primary objective of this study was to evaluate the tolerability, safety, and efficacy of a 7-day high-dose (1 mg/kg per day) primaquine regimen for the radical cure of vivax malaria in patients screened as G6PD normal.

## Methods

### Study design

This was a randomised, double-blind, placebo-controlled, non-inferiority trial in patients with normal G6PD and uncomplicated vivax malaria presenting at two health-care clinics in each of the following countries: Afghanistan, Ethiopia, Indonesia, and Vietnam (appendix pp 3–5). Ethical and drug regulatory approvals were obtained from the relevant national and local committees and authorities, the Oxford Tropical Research Ethics Committee (1014–13), and the Human Research Ethics Committee of the Northern Territory Department of Health, Australia (13–1991; appendix pp 6–7). The study protocol has been published previously.^20^

At the time of the study, primaquine radical cure was national policy in Indonesia, Vietnam, and Afghanistan, but not Ethiopia. The justification of a placebo control group in the Indonesia, Vietnam, and Afghanistan sites was based on clinical equipoise, considering the risks and benefits of different treatment regimens and the importance of controlling for variation in the risk of relapse. Rapid initial clinical cure was achieved with highly efficacious schizontocidal treatment, which was administered to all patients. Further details on the rationale for including a placebo control group have been published previously.^21^

### Participants

Patients presenting with uncomplicated vivax malaria (mono-infection or mixed infection) who had fever or a history of fever within the past 48 h, were aged older than 6 months, weighed at least 5 kg, had a haemoglobin concentration of 9 g/dL or more, and had normal G6PD status as assessed by the fluorescent spot test were eligible for enrolment. Patients were excluded if they were pregnant or lactating, could not tolerate oral treatment, had previous haemolytic episodes or blood transfusion within the past 90 days, or had signs of severe malaria. Other exclusion criteria included any hypersensitivity to study drugs, or concomitant medication with the potential to cause haemolysis or interfere with the pharmacokinetics of the study drugs.

Before enrolment, written informed consent was obtained from the patient or their guardian. Written assent was also obtained from patients who were 12–18 years old. Patients with G6PD deficiency were excluded from the randomised trial, but were enrolled into a parallel observational group and treated with chloroquine or dihydroartemisinin-piperaquine plus supervised primaquine (0·75 mg/kg) once a week for 8 weeks.

### Randomisation and masking

Eligible patients were treated with a blood schizontocidal drug and randomly assigned in a 2:2:1 ratio to a 7-day primaquine regimen, a 14-day primaquine regimen, or placebo. The allocation ratio of 2:2:1 was based on the sample size requirements for the non-inferiority comparison of a 7-day primaquine regimen with a 14-day regimen, and the superiority comparisons of the 7-day and 14-day primaquine regimens with the placebo. Randomisation was done using STATA version 14.1 (StataCorp, College Station, TX, USA), which generated blocks of 20 for each dosing band. The independent statistician who generated the randomisation list and selected code letters for primaquine or placebo was not otherwise involved in the conduct of the trial and did not visit any of the study sites. Identical primaquine and placebo tablets were produced by the same manufacturer (Centurion Laboratories; Vadodara, India) and packaged in blister packs (Bilcare Research; Pune, India). Randomisation in blocks of 20 was done at each site. Boxes of primaquine or placebo tablets for each of three bodyweight bands were provided and numbered sequentially. Patients were allocated randomly to a treatment group and given the next numbered box within the appropriate weight band of that group. Participants and all of the local study team were masked to treatment assignments.

### Procedures

All patients were treated as outpatients with the local first-line treatment for *P vivax*, which was chloroquine in Ethiopia, Afghanistan, and Vietnam and dihydroartemisinin-piperaquine in Indonesia, according to local guidelines (appendix p 4). The study drug (primaquine or placebo) was started on either day 0 or day 1. Patients in the 7-day primaquine group received 7 days of primaquine (1·0 mg/kg per day; 7·0 mg/kg total dose) followed by 7 days of daily placebo. Patients in the 14-day primaquine group received 14 days of primaquine (0·5 mg/kg per day; 7 mg/kg total dose). Patients in the placebo group received 14 days of daily placebo (90% starch, calcium phosphate). Primaquine was administered as 15 mg tablets. Treatment was fully supervised and was dosed according to bodyweight (appendix p 9). For children weighing less than 23 kg, tablets were dissolved in 5 mL of syrup and administered as a suspension (appendix p 10). In Indonesia, Vietnam, and Afghanistan, where primaquine radical cure is recommended in the national antimalarial guidelines, a 14-day course of primaquine was administered to all patients in the placebo group at the end of the study follow-up.

At enrolment, a medical history was taken, a physical examination was done, and antimalarial treatment with chloroquine or dihydroartemisinin-piperaquine was initiated. Primaquine or placebo was usually started on the day of enrolment, although sites had clinical discretion to start the next day. After completion of treatment, patients were asked to return weekly until day 42 and then monthly for 1 year. At each visit, a medical history was taken, a symptom questionnaire was done, and any adverse events or serious adverse events were recorded. Patients were encouraged to report to the study centre if they became ill. Patients who missed their scheduled follow-up visits were contacted by study staff and encouraged to return to the study centre for review. Blood films were examined immediately for all symptomatic patients, but otherwise stored for later examination. Recurrent parasitaemia with any *Plasmodium* species within 28 days was considered a treatment failure and treated with artemisinin-based combination therapy or 7 days of quinine, according to national guidelines. After 28 days, patients with recurrent symptomatic vivax parasitaemia were prescribed the same treatment allocated at enrolment. Patients presenting with their fourth (third in Vietnam) or subsequent symptomatic *P vivax* recurrence were treated with open-label supervised primaquine (0·5 mg/kg for 14 days).

At the initial screening, venous blood was collected and tested for G6PD deficiency with the qualitative fluorescent spot test (R&D Diagnostics; Athens, Greece). Standard thick (6 μL blood on a 12-mm diameter template) and thin malaria films were prepared at each visit for Giemsa staining (3%, 40–50 min) according to the WHO Research Malaria Microscopy Standards (appendix p 11). Blood films were considered negative if no parasites were detected after examining 200 high-power fields (magnification × 1000) on the thick film. Parasite counts were obtained from thick or thin films by counting the number of asexual parasites on a minimum of 40 high power fields or per 2000 erythrocytes. Parasite densities were estimated on the basis of calculated volumes of blood examined per 40 high power fields on the thick film or by assuming 5 × 10^6^ erythrocytes per μL for thin film counts. Microscopists were trained in study laboratory procedures on-site and continuous quality control was implemented at all sites. Approximately 10% of slides, including all the slides from day 0, the day of recurrent parasitaemia, and the 6-month follow-up visits were assessed periodically over the course of the trial by expert malaria microscopists at the Mahidol-Oxford Tropical Medicine Research Unit in Bangkok, Thailand. Results of the quality control procedures are presented in the appendix (pp 11–13). Haemoglobin concentration was checked at each visit (HemoCue; Ängelholm, Sweden). Venous and finger-prick blood samples were collected at predefined timepoints for molecular and serological analyses.

### Outcomes

The primary endpoint was defined as the incidence rate of symptomatic *P vivax* parasitaemia (mono-infection or mixed) over 12 months, with the primary analysis comparing this outcome in patients treated with 7-day and 14-day primaquine. The primary endpoint was calculated by site and then pooled across all sites. Secondary 12-month efficacy endpoints were the incidence rate and incidence risk (time-to-event analysis) of any (symptomatic or asymptomatic) *P vivax* parasitaemia, *Plasmodium falciparum* parasitaemia, or all-species parasitaemia, comparing the treatment groups to each other and with the placebo group. Other efficacy endpoints were the incidence risks of *P vivax* at 28 and 42 days, the proportion of patients with *P vivax* parasitaemia on days 1, 2, and 3, and the proportion with fever on days 1, 2, and 3. The number of recurrences avoided per 1000 patients and the number needed to treat (NNT) to avoid one recurrence were derived from the rate difference.

Safety endpoints were the incidence risk of severe anaemia (haemoglobin <7 g/dL) or transfusion, an acute drop in haemoglobin of more than 5 g/dL within 7 days, haemoglobin concentration on day 3 and day 7, the median time to haemoglobin nadir, grade 3 or 4 adverse events within 42 days, and severe adverse events.^22^ The relationship between treatment and adverse events or severe adverse events was determined by the site investigator. The tolerability of primaquine was assessed from the proportion of patients reporting each symptom from a routine questionnaire that was done at least once between days 1 and 14.

Efficacy and safety outcomes from the patients with G6PD deficiency who were given weekly primaquine and cost data will be reported separately.

### Statistical analysis

The original sample size calculation of 750 patients per primaquine group was based on an assumed incidence rate of 0·2 symptomatic *P vivax* recurrences per person-year in each group, a non-inferiority margin of 0·07 recurrences per person-year, a projected loss of 20% of patients to follow-up or protocol violations, a one-sided significance level of 2·5%, and a power of 80%. Two additional sites in Ethiopia were included to speed up recruitment. The 25% increase in sample size improved the generalisability of the study, and increased the power to detect non-inferiority between the 7-day and 14-day primaquine groups to 86%. Power calculations for superiority comparisons with the placebo group are provided in the appendix (p 8).

To assess the primary efficacy endpoint for radical cure, the absolute incidence rate difference (95% CI) in symptomatic *P vivax* recurrences per person-year between the 7-day and 14-day primaquine group was estimated using weighted least-squares regression with a robust standard error. For the secondary efficacy endpoints comparing 7-day primaquine versus placebo, 14-day primaquine versus placebo, and 7-day versus 14-day primaquine, incidence rate ratios were estimated using negative binomial regression (analysis of any recurrences). Cumulative incidence risks were estimated by Kaplan-Meier analysis. Hazard ratios (HRs) were estimated by Cox regression (analysis of time to the first recurrence). The main analysis was done on the intention-to-treat population. Details of censoring for incidence risk analyses, sensitivity analyses, investigation of study site heterogeneity, and safety analyses are in the appendix (pp 14–15). All analyses were done using STATA version 14.1.

The Data and Safety Monitoring Board did a blinded safety review every 6 months. The trial was registered at ClinicalTrials.gov (NCT01814683).

### Role of the funding source

The funders of the study had no role in study design, data collection, data analysis, data interpretation, or writing of the report. All authors had full access to the data in the study and had final responsibility for the decision to submit for publication.

## Results

**Figure 1:**
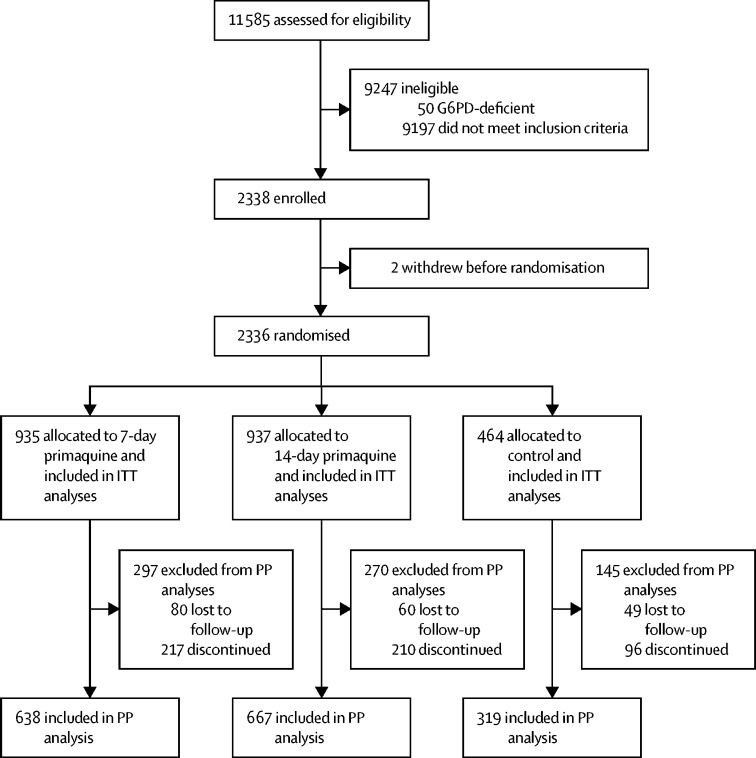
Trial profile G6PD=glucose-6-phosphate dehydrogenase. ITT=intention-to-treat. PP=per-protocol.

Between July 20, 2014, and Nov 25, 2017, 11 585 patients were screened for inclusion into the study, of whom 9197 did not meet the enrolment criteria and 50 were G6PD-deficient (figure 1). 2338 patients were enrolled into the main study, of whom two patients withdrew their consent before randomisation. The remaining 2336 patients were assigned to receive either 7-day primaquine (n=935), 14-day primaquine (n=937), or placebo (n=464; figure 1; appendix p 16). By country, 431 (18·5%) of 2336 patients were in Afghanistan, 580 (24·8%) were in Ethiopia, 1000 (42·8%) were in Indonesia, and 325 (13·9%) were in Vietnam.

Baseline patient characteristics were similar across the three groups (table 1). 1010 (43·2%) of 2336 patients were younger than 15 years old. The geometric mean *P vivax* parasitaemia was 3440 parasites per μL (95% normal range 141–53 037; appendix pp 17–18). The mean total dose of primaquine given was 7·39 mg base per kg (range 0·80–15·00) in the 7-day primaquine group and 7·39 mg base per kg (0·50–15·00) in the 14-day primaquine group. In the 7-day primaquine group, 53 (5·7%) of 935 patients failed to complete treatment, compared with 37 (3·9%) of 937 in the 14-day primaquine group and 14 (3·0%) of 464 in the placebo group. For patients who completed a full course of treatment, the mean total dose was 7·53 mg base per kg (range 3·80–15·00) in the 7-day primaquine group and 7·54 mg base per kg (4·20–15·00) in the 14-day primaquine group (appendix pp 19–20).

**Table 1:** Baseline characteristics

		**Placebo (n=464)**	**7-day primaquine (n=935)**	**14-day primaquine (n=937)**	**Total (n=2336)**
Site					
	Jalalabad, Afghanistan	60 (12·9%)	125 (13·4%)	126 (13·4%)	311 (13·3%)
	Laghman, Afghanistan	23 (5·0%)	48 (5·1%)	49 (5·2%)	120 (5·1%)
	Arba Minch, Ethiopia	74 (15·9%)	149 (15·9%)	148 (15·8%)	371 (15·9%)
	Metahara, Ethiopia	40 (8·6%)	85 (9·1%)	84 (9·0%)	209 (8·9%)
	Hanura, Indonesia	118 (25·4%)	229 (24·5%)	228 (24·3%)	575 (24·6%)
	Tanjung Leidong, Indonesia	85 (18·3%)	171 (18·3%)	169 (18·0%)	425 (18·2%)
	Dak O and Bu Gia Map, Vietnam	43 (9·3%)	87 (9·3%)	89 (9·5%)	219 (9·4%)
	Krong Pa, Vietnam	21 (4·5%)	41 (4·4%)	44 (4·7%)	106 (4·5%)
Age, years		17 (10–28)	16 (10–25)	16 (10–26)	16 (10–26)
Age category					
	6 to <12 months	1 (0·2%)	1 (0·1%)	1 (0·1%)	3 (0·1%)
	≥1 year to <5 years	28 (6·0%)	60 (6·4%)	63 (6·7%)	151 (6·5%)
	≥5 years to <15 years	163 (35·1%)	358 (38·3%)	335 (35·8%)	856 (36·6%)
	≥15 years	272 (58·6%)	516 (55·2%)	538 (57·4%)	1326 (56·8%)
Sex					
	Male	296 (63·8%)	563 (60·2%)	608 (64·9%)	1467 (62·8%)
	Female	168 (36·2%)	372 (39·8%)	329 (35·1%)	869 (37·2%)
Bodyweight, kg		47·0 (26·4–57·5)	46·2 (26·0–57·0)	46·8 (25·3–56·6)	46·6 (26·0–57·0)
Bodyweight category, kg					
	5·0–9·9	3 (0·6%)	8 (0·9%)	6 (0·6%)	17 (0·7%)
	10·0–22·9	89 (19·2%)	180 (19·3%)	180 (19·2%)	449 (19·2%)
	23·0–34·9	67 (14·4%)	136 (14·5%)	143 (15·3%)	346 (14·8%)
	35·0–45·9	64 (13·8%)	135 (14·4%)	121 (12·9%)	320 (13·7%)
	≥46·0	241 (51·9%)	476 (50·9%)	487 (52·0%)	1204 (51·5%)
*Plasmodium vivax* parasites per μL*		3702 (150–53 057)	3330 (144–55 000)	3426 (137–50 000)	3440 (141–53 037)
Gametocytes present		335 (72·2%)	713 (76·3%)	704 (75·1%)	1752 (75·0%)
Gametocytes per μL*		211 (15–3037)	200 (15–3815)	198 (15–2545)	201 (15–3037)
Body temperature, °C		37·9 (1·2)	37·8 (1·2)	37·8 (1·2)	37·8 (1·2)
Fever†		267 (57·9%)	537 (57·9%)	538 (57·5%)	1342 (57·8%)
Haemoglobin, g/dL		13·0 (1·7)	13·0 (1·8)	12·9 (1·7)	13·0 (1·7)
Haemoglobin <10 g/dL		19 (4·1%)	30 (3·2%)	40 (4·3%)	89 (3·8%)

Data are n (%), median (IQR), or mean (SD), unless otherwise indicated.

* Data are geometric mean (95% normal range).

† Defined as axillary temperature of >37·5°C or oral temperature of >38°C.

Fever clearance times were similar between groups, with 2145 (92·9%) of 2309 patients becoming afebrile within 24 h of starting treatment. Overall, 2217 (96·0%) of 2310 patients cleared peripheral parasitaemia within 2 days of starting treatment, with no significant difference between treatment groups (appendix p 21).

**Figure 2:**
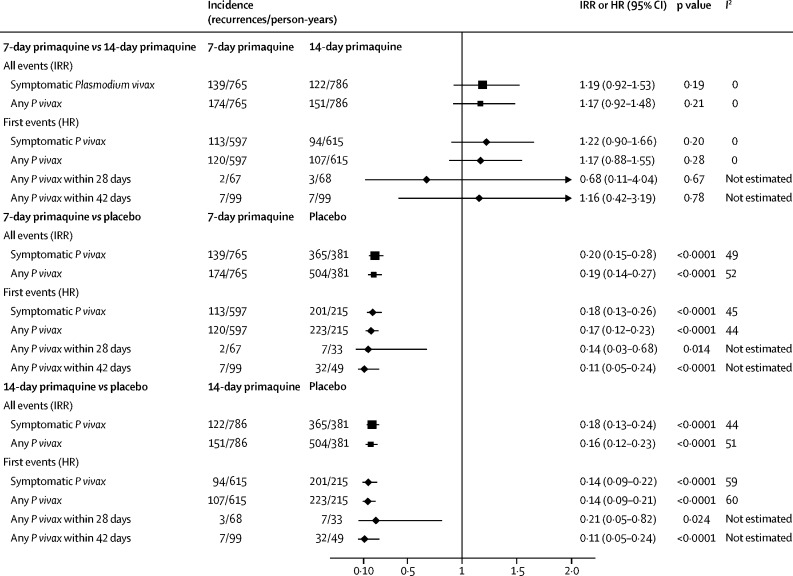
Forest plot of IRRs and HRs IRR=incidence rate ratio. HR=hazard ratio.

The incidence rate of symptomatic recurrent *P vivax* malaria was 0·18 recurrences per person-year (95% CI 0·15 to 0·21) in the 7-day primaquine group, 0·16 (0·13 to 0·18) in the 14-day primaquine group, and 0·96 (0·83 to 1·08) in the placebo group (table 2, figure 2, appendix pp 22–23). The incidence rate difference between the 7-day and 14-day groups was 0·02 recurrences per person-year (95% CI −0·02 to 0·05, p=0·34), which was within the predefined non-inferiority margin of 0·07 recurrences per person-year. The incidence rate ratio for 7-day versus 14-day primaquine was 1·19 (95% CI 0·92 to 1·53, p=0·18; table 2). The number of recurrences avoided per 1000 patients was 781 for the 7-day primaquine group (NNT 1·28, 95% CI 1·12 to 1·56) and 800 for the 14-day primaquine group (1·25, 1·09 to 1·52). The results of site-specific, per-protocol, and all a priori sensitivity analyses are presented in the appendix (pp 23–28).

**Table 2:** Incidence rate of parasitaemia

	**Incidence rate per person-year (95% CI)**			**7-day primaquine *vs* control**		**14-day primaquine *vs* control**		**7-day primaquine *vs* 14-day primaquine**	
	Placebo (n=464)	7-day primaquine (n=935)	14-day primaquine (n=937)	Incidence rate ratio	p value	Incidence rate ratio	p value	Incidence rate ratio	p value
Total patient days of follow-up*	139 258	279 371	287 200	..	..	..	..	..	..
Symptomatic *Plasmodium vivax* parasitaemia	0·96 (0·83–1·08)	0·18 (0·15–0·21)	0·16 (0·13–0·18)	0·20 (0·15–0·28)	<0·0001	0·18 (0·13–0·24)	<0·0001	1·19 (0·92–1·53)	0·18
Any *Plasmodium vivax* parasitaemia	1·32 (1·16–1·48)	0·23 (0·19–0·27)	0·19 (0·16–0·23)	0·19 (0·14–0·27)	<0·0001	0·16 (0·12–0·23)	<0·0001	1·17 (0·92–1·48)	0·21
Any *Plasmodium falciparum* parasitaemia	0·10 (0·05–0·15)	0·15 (0·11–0·19)	0·10 (0·07–0·14)	1·22 (0·80–1·85)	0·36	0·97 (0·63–1·49)	0·89	1·25 (0·89–1·76)	0·19
Parasitaemia of any species	1·42 (1·25–1·59)	0·38 (0·32–0·44)	0·30 (0·24–0·35)	0·25 (0·19–0·33)	<0·0001	0·21 (0·16–0·27)	<0·0001	1·23 (0·99–1·52)	0·061

* From the first visit to the end of follow-up, or until the last visit if lost.

The incidence rate of any (symptomatic or asymptomatic) recurrent vivax malaria at 1 year was 0·23 recurrences per person-year (95% CI 0·19–0·27) in the 7-day primaquine group and 0·19 (0·16–0·23) in the 14-day primaquine group (p=0·21; table 2, figure 2, appendix pp 29–36). These incidence rates were significantly lower than in the placebo group, for which the incidence rate was 1·32 recurrences per person-year (95% CI 1·16–1·48). The incidence rate ratio was 0·19 (95% CI 0·14–0·27, p<0·0001) for 7-day primaquine versus placebo and 0·16 (0·12–0·23, p<0·0001) for 14-day primaquine versus placebo.

**Figure 3:**
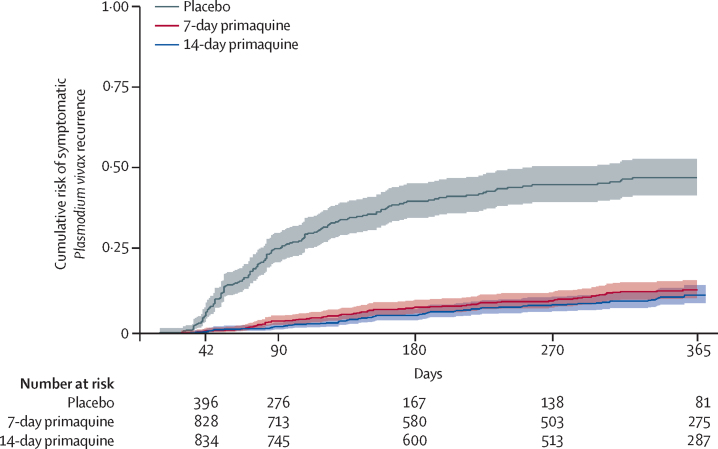
Cumulative incidence of the first symptomatic recurrence of *Plasmodium vivax* parasitaemia

In the time-to-event analysis, the incidence risk of symptomatic *P vivax* at 1 year was 14·28% (95% CI 11·75–17·29) in the 7-day primaquine group and 12·72% (10·19–15·82) in the 14-day primaquine group (p=0·20; table 3). The incidence risk was significantly lower in both treatment groups than in the placebo group, for which the incidence risk was 48·73% (95% CI 43·40–54·36; figure 3). At the two Indonesian sites, the incidence risks of symptomatic vivax recurrence were 39·33% (95% CI 30·31–49·92) and 28·59% (19·39–40·90) after treatment with dihydroartemisinin-piperaquine alone compared with incidence risks of more than 50% at the other sites, where chloroquine was used (appendix p 37).

**Table 3:** Incidence risk of parasitaemia

	**Incidence risk, % (95% CI)**			**7-day primaquine *vs* control**		**14-day primaquine *vs* control**		**7-day primaquine *vs* 14-day primaquine**	
	Placebo (n=464)	7-day primaquine (n=935)	14-day primaquine (n=937)	HR (95% CI)	p value	HR (95% CI)	p value	HR (95% CI)	p value
**28 days**									
Any *Plasmodium vivax* parasitaemia	1·71% (0·82–3·56)	0·23% (0·06–0·93)	0·33% (0·10–1·00)	0·14 (0·03–0·68)	0·014	0·21 (0·05–0·82)	0·024	0·68 (0·11–4·04)	0·67
**42 days**									
Any *Plasmodium vivax* parasitaemia	7·88% (5·64–10·97)	0·87% (0·41–1·81)	0·82% (0·39–1·72)	0·11 (0·05–0·24)	<0·0001	0·11 (0·05–0·24)	<0·0001	1·16 (0·42–3·19)	0·78
**1 year**									
Symptomatic *Plasmodium vivax* parasitaemia	48·73% (43·40–54·36)	14·28% (11·75–17·29)	12·72% (10·19–15·82)	0·18 (0·13–0·26)	<0·0001	0·14 (0·09–0·22)	<0·0001	1·22 (0·90–1·66)	0·20
Any *Plasmodium vivax* parasitaemia	58·40% (53·28–63·61)	15·99% (13·35–19·08)	14·75% (12·08–17·93)	0·17 (0·12–0·23)	<0·0001	0·14 (0·09–0·21)	<0·0001	1·17 (0·88–1·55)	0·28
Any *Plasmodium falciparum* parasitaemia	11·40% (7·66–16·78)	8·67% (6·80–11·03)	7·22% (5·51–9·45)	1·22 (0·80–1·85)	0·36	0·97 (0·63–1·49)	0·89	1·25 (0·89–1·76)	0·19
Parasitaemia of any species	1·42% (1·25–1·59)	0·38% (0·32–0·44)	0·30% (0·24–0·35)	0·22 (0·15–0·32)	<0·0001	0·18 (0·13–0·26)	<0·0001	1·22 (0·97–1·53)	0·091

HR=hazard ratio.

At the end of the 1-year follow-up, the incidence rate of symptomatic *P falciparum* infection was 0·10 (95% CI 0·07–0·13) recurrences per person-year in the 7-day primaquine group, 0·07 (0·05–0·09) in the 14-day primaquine group, and 0·07 (0·04–0·10) in the control group.

14 (0·6%) of 2336 patients had recurrent *P vivax* parasitaemia within 28 days. The incidence risk of any (symptomatic or asymptomatic) *P vivax* parasitaemia at day 28 was 0·23% (95% CI 0·06–0·93) in the 7-day primaquine group, 0·33% (0·10–1·00) in the 14-day primaquine group, and 1·71% (0·82–3·56) in the placebo group. At 42 days, the incidence risk of any *P vivax* parasitaemia was 0·87% (95% CI 0·41–1·81) in the 7-day primaquine group and 0·82% (0·39–1·72) in the 14-day primaquine group (p=0·78). Compared with the placebo group, the HR was 0·11 (95% CI 0·05–0·24, p<0·0001) for both treatment groups (table 3, Figure 2, Figure 3).

Reporting of serious adverse events and adverse events varied significantly by site (appendix pp 38–43). 27 serious adverse events were reported (table 4). Ten serious adverse events occurred within 42 days that were considered to be related to the study drug, mostly in the 7-day primaquine group (table 4). Four of the serious adverse events were significant haemolysis, which occurred 3–5 days after starting treatment (table 4). One of the patients with significant haemolysis was a Vietnamese man with G6PD deficiency who was erroneously enrolled into the 7-day primaquine group; he required a blood transfusion after his haemoglobin decreased from 15·3 g/dL to 6·4 g/dL. The two other patients with significant haemolysis in the 7-day primaquine group were women; their haemoglobin concentrations decreased from 11·6 g/dL to 6·9 g/dL and from 13·7 g/dL to 9·5 g/dL. One man in the 14-day primaquine group had significant haemolysis with a decrease in haemoglobin from 10·2 g/dL to 6·8 g/dL. All three patients recovered within 1–3 days of stopping the study drug and none required a blood transfusion. Other serious adverse events attributed to the study drugs included five patients in the 7-day primaquine group who had gastrointestinal symptoms that were severe enough to discontinue treatment temporarily; all subsequently restarted the study drug and completed a full course. One patient in the 7-day group developed bronchospasm and methaemoglobinaemia on day 10. The median duration of the drug-related serious adverse events was 3·5 days (range 1–13). One patient died: a 66-year-old man in the 7-day primaquine group who had a myocardial infarction 5 months after enrolment. This event was deemed to be unrelated to the study drug (appendix pp 38–40).

**Table 4:** Safety endpoints

	**Placebo (n=464)**	**7-day primaquine (n=935)**	**14-day primaquine (n=937)**
**≤1 h**			
Vomiting study drug	11 (2·4%)	21 (2·2%)	20 (2·1%)
1–14 days*			
Vomiting	62 (13·4%)	192 (20·5%)	139 (14·8%)
Headache	225 (48·5%)	480 (51·3%)	465 (49·6%)
Nausea	153 (33·0%)	346 (37·0%)	321 (34·3%)
Diarrhoea	24 (5·2%)	95 (10·2%)	52 (5·5%)
Skin rash	13 (2·8%)	22 (2·4%)	26 (2·8%)
Poor appetite	170 (36·6%)	407 (43·5%)	374 (39·9%)
Abdominal pain	133 (28·7%)	400 (42·8%)	318 (33·9%)
Myalgia or arthralgia	118 (25·4%)	243 (26·0%)	236 (25·2%)
Fever	148 (31·9%)	294 (31·4%)	319 (34·0%)
Passing dark urine	23 (5·0%)	55 (5·9%)	48 (5·1%)
Dizziness	77 (16·6%)	166 (17·8%)	157 (16·8%)
Shortness of breath	19 (4·1%)	33 (3·5%)	29 (3·1%)
Itching	14 (3·0%)	24 (2·6%)	26 (2·8%)
Any gastrointestinal symptoms†	231 (49·8%)	558 (59·7%)	513 (54·7%)
**≤42 days**			
SAE (primaquine-related; all severities)‡	0	9 (1·0%)	1 (0·1%)
SAE (primaquine-unrelated; all severities)	4 (0·9%)	3 (0·3%)	0
AE (grades 3 and 4)§	0	7 (1·4%)	1 (0·1%)
**≤1 year**			
SAE (primaquine-related; all severities)	0	9 (1·0%)	1 (0·1%)
SAE (primaquine-unrelated; all severities)	4 (0·9%)	9 (1·0%)	4 (0·4%)

Data are the proportion of patients reporting each symptom at least once (n [%]). SAE=serious adverse event. AE=adverse event.

* Symptoms elicited from daily questionnaires, 1–14 days after starting antimalarial treatment.

† Composite of any of the following: nausea, vomiting, anorexia, diarrhoea, or abdominal pain.

‡ Related events include those that are possibly, probably, or definitely related.

§ Within 42 days of enrolment; excludes SAEs; all events were grade 3.

Adverse events within 42 days with a severity grade of 3 or 4 were reported in eight patients, mostly in the 7-day primaquine group (table 4, appendix p 44). Six of the adverse events were gastrointestinal symptoms and five occurred within the first 6 days of treatment. 21 (2·2%) of 935 patients in the 7-day group and 20 (2·1%) of 937 patients in the 14-day group vomited the study drugs within an hour, compared with 11 (2·4%) of 464 in the placebo group (table 4). In the 7-day primaquine group, 558 (59·7%) of 935 patients reported gastrointestinal symptoms during their course of treatment, which was significantly more than in the 14-day group (513 [54·7%] of 937, p=0·035) or the placebo group (231 [49·8%] of 464, p=0·0006; table 4, appendix pp 45–52). The median duration of gastrointestinal symptoms was 3 days (IQR 2–5) in the 7-day primaquine group, compared with 2 days (1–4) in the 14-day group or the placebo group (p<0·0001).

The median time to haemoglobin nadir was 3 days (IQR 3–7) in the placebo group and 3 days (3–13) in both primaquine groups. The mean absolute decreases in haemoglobin at day 3 were 0·52 g/dL (SD 1·19) in the 7-day primaquine group, 0·62 g/dL (1·09) in the 14-day group, and 0·72 g/dL (1·12) in the placebo group. The corresponding mean percentage change in haemoglobin between day 0 and day 3 was −3·60% (SD 9·17) in the 7-day group, −4·53% (8·47) in the 14-day group, and −5·26% (8·64) in the placebo group (appendix p 54). The mean absolute decreases in haemoglobin at day 7 were 0·01 g/dL (SD 1·23) in the 7-day group, 0·12 g/dL (1·09) in the 14-day group, and 0·31 g/dL (1·20) in the placebo group. The haemoglobin concentration decreased to 6 g/dL in four patients (three men and one woman; three patients in the 7-day group and one in the 14-day group). One male patient with normal G6PD in the 14-day group had an acute decrease in haemoglobin of more than 5 g/dL. 19 patients had a percentage decrease in haemoglobin of more than 25% between enrolment and day 3, (eight [0·9%] of 891 patients in the 7-day group, eight [0·9%] of 895 in the 14-day group, and three [0·7%] of 432 in the placebo group; appendix pp 53–55).

## Discussion

This large, multinational, placebo-controlled trial confirms the high antirelapse efficacy of primaquine at a total dose of 7 mg/kg and demonstrates non-inferiority of 7-day primaquine compared with 14-day primaquine. A supervised 7-day course of high-dose primaquine prevented an estimated 781 recurrences per 1000 patients, equivalent to an NNT of 1·28. By comparison, the 14-day course of primaquine prevented 800 recurrences per 1000 patients, equivalent to an NNT of 1·25. The 7-day course was associated with a slightly higher frequency of adverse events than the 14-day course, which were mostly attributable to gastrointestinal symptoms.

Primaquine has been used for the radical cure of *P vivax* malaria for more than 60 years. WHO recommends the use of primaquine after screening for G6PD deficiency, but in most malaria endemic countries, reliable G6PD testing is unavailable. Therefore, particularly in areas of high prevalence of G6PD deficiency, primaquine is rarely used because of concerns over safety.^17^ The antirelapse efficacy of primaquine is related to the total dose administered in the full treatment course, whereas haemolytic toxicity depends on the daily dose administered, particularly at the beginning of the treatment course.^23^, ^24^

For safety reasons, primaquine is widely recommended as a 14-day regimen; however, adherence to such a long course of treatment for an acute febrile illness can be low.^13^, ^14^ Interventions such as directly observed treatment ensure adherence and therefore improve effectiveness,^10^, ^25^ but these measures will be less costly and easier to implement for a short-course regimen.

Patients in the 7-day primaquine group in our study received twice the daily dose of those in the standard 14-day high-dose regimen (0·5 mg/kg per day) and four times the dose of the 14-day low-dose regimen (0·25 mg/kg per day) that is recommended by most national malaria control programmes.^15^ The main dose-limiting toxic effect of primaquine is related to upper gastrointestinal intolerance, which results in abdominal pain, nausea, and vomiting. Patients treated with 1 mg/kg per day of primaquine had a similarly low rate of early vomiting to those treated with 0·5 mg/kg per day; however, patients given 1 mg/kg per day were more likely to report gastrointestinal symptoms. The reported symptoms generally occurred early, were mild in severity, and resolved within 72 h. Gastrointestinal symptoms can result in reduced adherence to a complete course of treatment, and this might have contributed to a greater number of patients with incomplete treatment in the 7-day primaquine group compared with the placebo group (5·7% *vs* 3·0%). Ingesting primaquine with food can improve gastrointestinal tolerability considerably and should be encouraged in all patients reporting symptoms.^26^

In malaria-endemic areas, the most widely used test to diagnose G6PD deficiency is the fluorescent spot test, a qualitative assay that requires laboratory facilities and can only identify individuals with less than 30% enzyme activity.^27^ All of the patients enrolled into our study were screened for G6PD deficiency with the fluorescent spot test. Therefore, although G6PD-deficient hemizygous males and homozygous females will have been excluded, heterozygous females with intermediate G6PD activity (30–70%) will have tested as G6PD-normal and been enrolled. Patients in the 7-day primaquine group had slightly increased haemolysis compared with the 14-day group, but haemoglobin reductions were small. Haemoglobin decreased to less than 7 g/dL in only one patient with normal G6PD. Four haemolytic serious adverse events were reported, including one blood transfusion in a male patient with G6PD deficiency who was erroneously enrolled. Two of the three other haemolytic serious adverse events occurred in women who might have had intermediate G6PD activity. In a 2017 study of a high-dose (1 mg/kg per day) 7-day primaquine regimen, two of 34 heterozygous females required a blood transfusion,^23^ yet blood transfusions were not required in any of the 701 female patients in our study. Our findings suggest that with robust G6PD testing and vigilance for early signs of haemolysis, 7-day or 14-day primaquine treatment could be stopped in patients with early signs of haemolysis (haemoglobinuria or a substantial fall in haemoglobin) and the severe consequences of haemolysis could be avoided. However, the large-scale roll-out of short-course, high-dose primaquine regimens in resource-poor settings will require a broader agenda to educate communities and providers about the risks and benefits of radical cure.^17^

Tafenoquine, a slowly eliminated 8-aminoquinoline, was licensed in the USA and Australia in 2018. When combined with chloroquine, tafenoquine offers a single dose option for *P vivax* radical cure.^28^ Although tafenoquine overcomes the issue of adherence, the prolonged exposure caused by its slow elimination can result in protracted haemolysis, even in women who are heterozygous for G6PD deficiency.^29^ For this reason, the use of tafenoquine is restricted to patients with more than 70% G6PD enzyme activity. Diagnosis of G6PD deficiency at this more stringent threshold requires a quantitative assay, which adds cost and complexity to large-scale roll-out. Tafenoquine is currently only licensed for radical cure in patients aged 16 years or older, although clinical trials in children are ongoing. Primaquine is rapidly eliminated and thus treatment can be stopped at the first signs of haemolysis. Therefore, primaquine can generally be prescribed safely in people with G6PD enzyme activity greater than 30%. For this reason, the availability of a safe and effective radical cure with primaquine is a necessary and potentially more accessible therapeutic option for the foreseeable future.

A major strength of our study is its multicentre design, with patients enrolled from four vivax-endemic countries with diverse patient populations and relapse periodicity. Therefore, our findings are likely to be generalisable to other vivax-endemic regions. Our study also has several limitations. Approximately one third of patients did not complete the 1-year follow-up, mainly because of the late start of the study in Ethiopia, where a high proportion of patients were unable to complete follow-up before study termination. However, the primary outcome, incidence rate of recurrent *P vivax* parasitaemia, was not compromised because the additional Ethiopian sites ensured that the total number of patient days of follow-up exceeded that required to achieve 80% power for non-inferiority. In addition, a significant difference was found in the risk of adverse events between sites, which probably reflected differences in recording and perception of symptoms rather than true differences in events between the patient cohorts. Although most adverse events were reported from the site in Hanura, Indonesia, the double-blinded treatment allocation means that this is unlikely to have affected the robustness of the comparison between treatment regimens.

In conclusion, the efficacy of a shorter 7-day course of primaquine was non-inferior to the standard 14-day course in patients with normal G6PD and had an acceptable safety and tolerability profile. The reduced time of treatment has the potential to improve adherence and therefore the effectiveness of radical cure in vivax-endemic countries. The high-dose 7-day primaquine regimen was well tolerated, but it requires that G6PD deficiency can be excluded reliably. In malaria-endemic countries, G6PD testing at the 30% threshold can now be achieved using point-of-care qualitative and quantitative rapid diagnostic tests,^30^, ^31^ which could allow for the widespread delivery of a safe and effective short-course primaquine regimen.

## Data sharing

Deidentified individual participant data will be available immediately after publication to applicants who provide a sound proposal to the Mahidol Oxford Tropical Medicine Research Unit Data Access Committee.
