# Supplementary material for: Short-course primaquine for the radical cure of Plasmodium vivax malaria: a multicentre, randomised, placebo-controlled non-inferiority trial
Source: Lancet. 2019 Sep 14;394(10202):929–38. doi: 10.1016/S0140-6736(19)31285-1 (PMC6753019; doi:10.1016/S0140-6736(19)31285-1)
Supplement: Supplementary appendix [file mmc1.pdf]

THE LANCET

Supplementary appendix

This appendix formed part of the original submission and has been peer reviewed. We post it as supplied by the authors.

Supplement to: Taylor WRJ, Thriemer K, von Seidlein L, et al. Short-course primaquine for the radical cure of *Plasmodium vivax* malaria: a multicentre, randomised, placebo-controlled non-inferiority trial. *Lancet* 2019; published online July 18. http://dx.doi.org/10.1016/S0140-6736(19)31285-1.

Supplementary Appendices

Supplement to: Short course primaquine for the radical cure of Plasmodium vivax malaria: a randomised placebo-controlled multicentre trial

Table of Contents

1. Map of Study Sites 3

2. Information About Study Sites 4

3. List of Ethics Review Boards and Regulatory Agencies 6

4. Sample Size and Power Calculation 8

5. Primaquine Dosing of Tablets 9

6. Primaquine Suspension Dosing of Children Under 23 kgs 10

7. Microscopy Quality Control Results 11

8. Statistical Methods 14

9. Consort Flow Chart 16

10. Site-specific Baseline Tables 17

11. Scatterplots of Total Dose of PQ Received by Weight 19

12. Adherence and Total Dose of Drug Received 20

13. Parasite and Fever Clearance 21

14. Histogram of the number of symptomatic P. vivax recurrences in each treatment arm 22

15. Site Specific Results for the Primary Endpoint – Incidence Rate of Recurrent Symptomatic Episodes of P. vivax within 12 months Follow up 23

16. Per protocol Analysis 24

17. Sensitivity Analyses in the ITT Population 25

18. Site-Specific ITT Hazard Ratios (HR) and Incidence Rate Ratios (IRR) 28

a) PQ7 vs PQ14: Risks and rates for recurrent parasitemia due to symptomatic and any infection due to *P. vivax* 28

b) PQ7 vs PQ14: Risks and rates for recurrent parasitemia due to symptomatic and any infection due to *P. falciparum* 29

c) PQ7 vs Pq14: Risks and rates for recurrent parasitemia due to symptomatic and any infection due to all *Species* 30

d) PQ7 vs Control: Risks and rates for recurrent parasitemia due to symptomatic and any infection due to *P. vivax* 31

e) PQ7 vs Control: Risks and rates for recurrent parasitemia due to symptomatic and any infection due to *P. falciparum* 32

f) PQ7 vs Control: Risks and rates for recurrent parasitemia due to symptomatic and any infection due to all *species* 33

g) PQ14 vs Control: Risks and rates for recurrent parasitemia due to symptomatic and any infection due *P. vivax* 34

h) PQ14 vs Control: Risks and rates for recurrent parasitemia due to symptomatic and any infection due *P. falciparum* 35

i) PQ14 vs Control: Risks and rates for recurrent parasitemia due to symptomatic and any infection due all species 36

19. Site-specific Kaplan Meier Curves for Symptomatic P. vivax Recurrence 37

20. Details of SAEs 38

21. Site Specific Adverse Events 41

22. Details of AEs 44

23. Symptoms Elicited from Daily Questionnaires During Treatment between Days 1 and 3 45

24. Symptoms elicited from daily questionnaires during treatment – Days 4 to 14 49

25. Distribution of haemoglobin (g/dL) during follow up by treatment arm 53

26. Haemoglobin Profile by Treatment Arm 54

1. Map of Study Sites

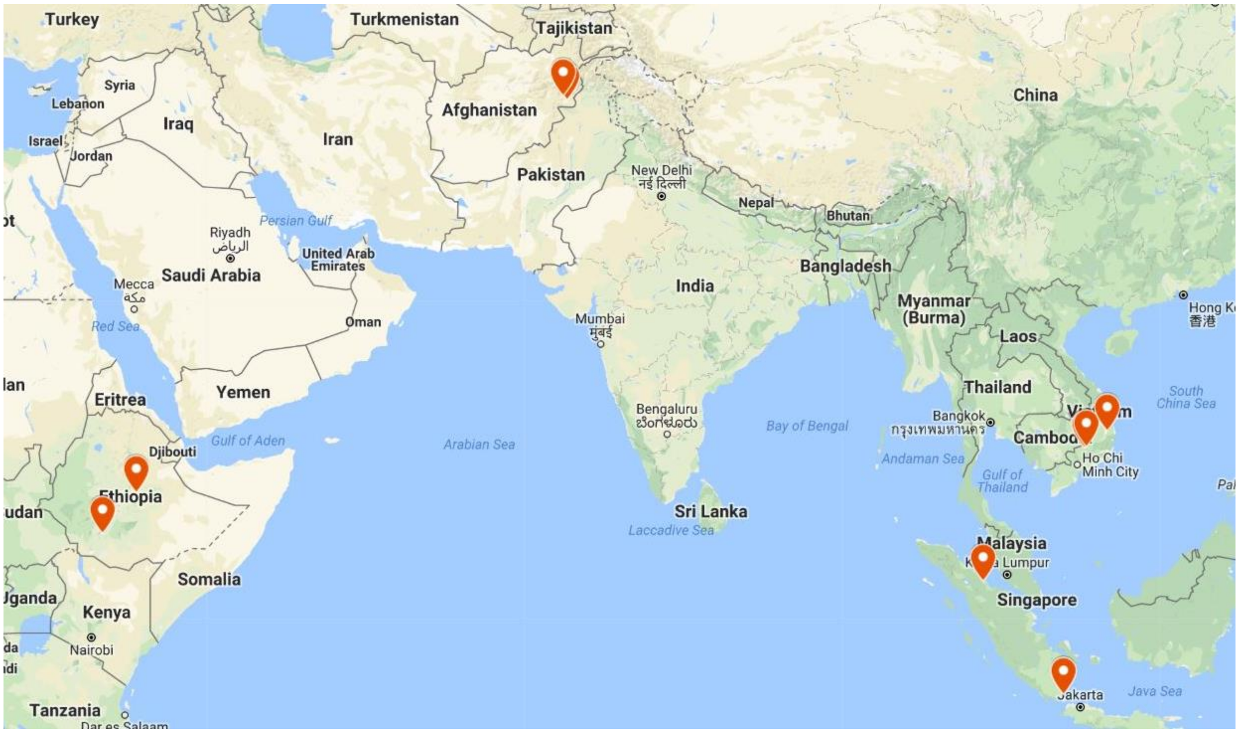

2. Information About Study Sites

	Afghanistan		Ethiopia		Indonesia		Vietnam	
**Site Name**	**Jalalabad**	**Laghman**	**Arba Minch**	**Metahara**	**Hanura**	**Tanjung Leidong**	**Dak O & Bu Gia Map**	**Krong Pa**
**Site Code**	AF001	AF008	ET001	ET002	ID004	ID005	VN001	VN002
**Name of study center**	Speen-Ghar Teaching Hospital	Laghman, Mihterlam hospital	Arba Minch Hospital	Metehara Sugar Factory Hospital	Hanura Primary Health Center	Tanjung Leidong	Dak-O & Bu Gia Map Commune Health Stations	Krong Pa Medical Center
**Study center GPS coordinates**	34.4344/ 70.4612	34.6891/ 70.1467	6.025204/ 37.556696	8.8875/ 39.9169444	-5.320619/ 105.622958	2.698056/ 99.901667	12.047594/ 107.093178	13.221819/ 108.682475
**Altitude above sea level**	575 m	779m	1285m	946 m	15 – 65 m	5m	338-408 m	136 m
**Treatment policy (incl. G6PD testing)**	**Pf:** AL+PQ (single dose 0.25 mg/kg)<br>**Pv:** CQ+PQ (0.25 mg/kg for 14 days)<br>**G6Pd testing** recommended. If not available, weekly PQ course recommended		**Pf:** AL+PQ (single dose 0.25 mg/kg)<br>**Pv:** CQ+PQ (0.25 mg/kg for 14 days)<br>**G6PD testing** not explicitly recommended		**Pf:** DHP+PQ (single dose 0.25 mg/kg)<br>**Pv:** DHP+PQ (0.25 mg/kg for 14 days).<br>**G6PD testing** not explicitly recommended		**Pf**: DHP+PQ (single dose 0.25 mg/kg)<br>**Pv**: CQ+PQ<br>(0.25 mg/kg for 14 days)<br>**G6PD testing** not explicitly recommended	
**G6PD prevalence and variants**	Mediterranean variant 7% in many ethnic groups[1]	Mediterranean variant 7% in many ethnic groups[1]	Unknown, likely A- and Mediterranean	Unknown, likely A- and Mediterranean	Unknown	2.7%, Mahidol variant[2]	Vientiane & Mahidol<br>(Kinh = 2%<br>Ethnic minorities: 10%)[3]	Vientiane & Mahidol<br>(Kinh = 2%<br>Ethnic minorities: 10%)[3]

[1] Jamornthanyawat N, Awab GR, Tanomsing N, *et al* A Population Survey of the Glucose-6-Phosphate Dehydrogenase (G6PD) 563C>T (Mediterranean) Mutation in Afghanistan. *PLoS One* 2014; 9(2):e88605.

[2] Pasaribu AP, Chokejindachai W, Sirivichayakul C, *et al.* A randomised comparison of dihydroartemisinin-piperaquine and artesunate-amodiaquine combined with primaquine for radical treatment of vivax malaria in Sumatera, Indonesia. *J Infect Dis* 2013 ;208(11):1906-13. doi: 10.1093/infdis/jit407.

[3] Personal communication Hien Tran Tinh

**Approx. catchment population of study center**	356,274	100,000	164,529	22,0259[7]	35,692	21,191	22,000	70,000
**Seasonality of malaria**	May to Nov (peak in summer /July), Pf is confined to Oct-Nov)	May to Nov (peak in summer /July), Pf is confined to Oct-Nov)	Year round with peak at Dec- Feb	Peak malaria Sept-October, smaller season April	Peak season November and march	Year round, peak between July-December	Peak malaria season October to April	Peak malaria season October to January
**P.v./ P.f. ratio**	95:5	90:10	30:70	40:60[7]	40:60[4]	95:5	40:60	40:60
**Relapse periodicity**			3-5 months[5]	3-5 months[5]	3 months	3 months	45 days	3 months
**Vectors**	*An. stephensi*<br>*An. culicifacies*<br>*An. fluviatilus*<br>*An. Annularis*<br>*An. Pulcherrimus*<br>*An. superpictus*<br>*An. hycranus*[6]	*A. stephensi*<br>*A. subpictus*and<br>*A. culicifacies*	*A.arabiensis*[7]	*A.arabiensis*[7]	*A. sundaicus*[8]	Not known, probably A. sundaicus	*A. dirus*<br>*A. minimus*[9]	*A. dirus*<br>*A. minimus*[9]
**API**	3.5 per 1,000 per year[10]	1.62 per 1,000 per year	unknown	unknown	43.9 per 1,000 per year	49 per 1,000 per year[11]	50 per 1,000 year[12]	8.5 per 1,000 year[12]

[4] Based on screening data from within IMPROV study

[5] Abreha T, Hwang J, Thriemer K, *et al*. Comparison of artemether-lumefantrine and chloroquine with and without primaquine for the treatment of Plasmodium vivax infection in Ethiopia: A randomised controlled trial. *PLoS Med* 2017 ;14(5):e1002299. doi: 10.1371/journal.pmed.1002299

[6] Rowland M, Mohammad N, Rehman H, *et al*. Anopheline vectors and malaria transmission in eastern Afghanistan. *Trans. R. Soc. Trop. Med. Hyg*. 2002; 15 (11): 17530-9. .

[7] Based on Federal democratic republic of Ethiopia population census commission, summary and statistical report of the 2007 population and housing census. 2008

[8] Sukowati S, Shinta, Suwito, *et al*. Some aspect of behavior of Malaria vector Anopheles sundaicus (Theobald) in Lampung, Sumatera. *J Ekol Kesehat.* 2011;10(4):267–78.

[9] Son DH, Thuy-Nhien N, von Seidlein L, *et al.* The prevalence, incidence and prevention of Plasmodium falciparum infections in forest rangers in Bu Gia Map National Park, Binh Phuoc province, Vietnam: a pilot study. *Malar J.* 2017; 16: 444.

[10] Based on the national strategic plan for malaria control to elimination in Afghanistan 2018-2022 by the Ministry of Public Health, Afghanistan

[11] Based on data from the Ministry of Health on annual parasite incidence (API) in 2016

[12] Based on reports of the National Malaria Control Program (NMCP of Vietnam) in 2017

3. List of Ethics Review Boards and Regulatory Agencies

**Australia**	**The Human Research Ethics Committee of the Northern Territory Department of Health (HREC)**<br>John Mathews Building (Bldg 58)<br>Royal Darwin Hospital Campus, Rock<br>PO Box: 41096, Casuarina NT 0811, Australia<br>Website: www.menzies.edu.au
**United Kingdom**	**The Oxford Tropical Research Ethics Committee (OxTREC)**<br>University of Oxford<br>Research Services, University Offices<br>Willington Square, Oxford OX1 2JD<br>Tel: +44 (0) 1865 (2) 82106<br>E-mail: oxtrec@admin.ox.ac.uk<br>Website: www.admin.ox.ac.uk/rso/
**Afghanistan**	**Islamic Republic of Afghanistan, Ministry of Public Health, Institutional Review Board**<br>3rd floor of Main Building of Ministry of Public Health<br>Masoud Circle, Wazir Akbar Khan, Kabul-Afghanistan<br>Tel: +93(0) 20 29 22 435<br>email: anphi@moph.gov.af
**Ethiopia**	**The National Research Ethics Review Committee (NRERC)**<br>Addis Ababa, Ethiopia<br>PO Box: 2490<br>Tel: +251 114-674-353<br>E-mail: most@ethionet.et<br>Fax: +251 114-660-241<br>Website: www.most.gov.et
	**Scientific & Ethical Review Committee (SERC)**<br>Ethiopian Public Health Institute<br>Addis Ababa, Ethiopia<br>PO Box: 1242/5654<br>E-mail: ephi@ethionet.et<br>Tel: +251 11 2133499, +251 11 2751522<br>Fax: +251 11 2758634<br>Website: www.ephi.gov.et
	**The Food Medicine and Health Care Administration and Control Authority (FMHACA)**<br>Addis Ababa, Ethiopia<br>Tel: 251-11-552 41 22/552 41 23<br>E-mail: regulatory@fmaca.gov.et<br>Fax: 251-11-552 13 92<br>PO Box: 5681

**Indonesia**	**The Health Research Ethics Committee of the Faculty of Medicine University of Indonesia**<br>Cipto Mangunkusumo Hospital<br>Jalan Salemba Raya No. 6, Jakarta Pusat 10430<br>Tel: 021-3157008<br>E-mail: ec_fkui@yahoo.com
	**Indonesian Food and Drug Agency (BPOM)**<br>Jl, Percetakan Negara No. 23 Jakarta Pusat 10560 Indonesia<br>E-mail: infopom@indo.net.id;<br>Tel: (021) 4244691, 4209221, 4263333, 4244755, 4241781, 4244819<br>Fax: (02) 4245139<br>Website: www.pom.go.id
**Vietnam**	**The Ministry of Health Evaluation Committee on Ethics in Biomedical Research**<br>138A Giang Vo Street, Ba Dinh District, Ha Noi, Viet Nam<br>Email: iecmoh@gmail.com<br>Tel: +84 4 6273 2156

4. Sample Size and Power Calculation

The primary aim of this trial was to demonstrate non-inferiority of a 7-day primaquine regimen to the standard 14-day primaquine regimen with respect to incidence rate of symptomatic vivax parasitaemia over 12 months. The sample size calculation was based on an assumed incidence rate of 0.2 infections per person-year in both arms, a non-inferiority margin of 0.07 infections per person-year and a one-sided significance level of 2.5%. Based on these assumptions, a total sample size of 1200 evaluable patients, randomly allocated to receive a primaquine regimen (600 patients in each treatment arm), followed for one year were calculated to provide a power of 80% to show non-inferiority or, equivalently, that the two-sided 95% confidence interval for the difference in incidence rate of malaria between the two arms excludes an excess rate of 0.07 infections per person-year or more in favour of the 14-day regimen.

A further 300 patients in the control arm were also followed for one year. With 300 patients in the control arm and 600 patients in each treatment arm (i.e. 75 controls and 150 patients in each treatment arm at each of the five study sites), the study had 95% power and 95% confidence to detect a difference (i.e. a superiority comparison of either regimen) at each of the study sites assuming an incidence rate of 0.2 infections per person-year in each of the treatment arms and 0.6 infections per person-year in the control arm (ranging from 0.2 in the Indian subcontinent and 1.0 in Vietnam and Indonesia). The combined proportion of losses to follow-up and major protocol violations was expected to be no more than 20%, so to account for this, a total of 1875 G6PD normal patients (750 per treatment arm; 375 in the control arm) were aimed to be randomised in this trial.

The inclusion of two additional sites in Ethiopia increased the sample size by 25% and increased the power to detect non-inferiority between the PQ7 and PQ14 arms to 86%, and the power to detect superiority between PQ7/PQ14 and placebo to 99.8%.

5. Primaquine Dosing of Tablets

Patient weight was rounded to the nearest integer

Weight Band	Weight/kg	PQ7		PQ14	
		1 mg/kg/day	mg/kg	0.5 mg/kg/day	mg/kg
A	5 to 22	see suspension dosing chart		see suspension dosing chart	
A	23-34	30	0.88 - 1.3	15	0.44 - 0.65
B	35-45	45	1 - 1.29	22.5	0.5 - 0.64
C	≥ 46	60	≤1.33	30	≤0.67

6. Primaquine Suspension Dosing of Children Under 23 kgs

Weight	ml/day	7 day Primaquine Regimen (PQ7)<br>Two 15mg tablets dissolved in 5ml syrup (1ml=6mg)				14 day Primaquine regimen (PQ14)<br>One 15mg tablet dissolved in 5ml syrup (1ml=3mg)			
		Daily dose in mg/day	Daily dose in mg/kg/day	Total dose in mg/kg	Total dose in mg	Daily dose in mg/day	Daily dose in mg/kg/day	Total dose in mg/kg	Total dose in mg
5	0.8	4.8	0.96	6.72	33.6	2.4	0.48	6.72	33.6
6	1	6	1	7	42	3	0.5	7	42
7	1.2	7.2	1.03	7.2	50.4	3.6	0.51	7.2	50.4
8	1.3	7.8	0.98	6.83	54.6	3.9	0.49	6.83	54.6
9	1.5	9	1	7	63	4.5	0.5	7	63
10	1.7	10.2	1.02	7.14	71.4	5.1	0.51	7.14	71.4
11	1.8	10.8	0.98	6.87	75.6	5.4	0.49	6.87	75.6
12	2	12	1	7	84	6	0.5	7	84
13	2.2	13.2	1.02	7.11	92.4	6.6	0.51	7.11	92.4
14	2.3	13.8	0.99	6.9	96.6	6.9	0.49	6.9	96.6
15	2.5	15	1	7	105	7.5	0.5	7	105
16	2.5	15	0.94	6.56	105	7.5	0.47	6.56	105
17	3	18	1.06	7.41	126	9	0.53	7.4	126
18	3	18	1	7	126	9	0.5	7	126
19	3	18	0.95	6.63	126	9	0.47	6.63	126
20	3	18	0.9	6.3	126	9	0.45	6.3	126
21	3.5	21	1	7	147	10.5	0.5	7	147
22	3.5	21	0.95	6.68	147	10.5	0.48	6.68	147

7. Microscopy Quality Control Results

Standard thick (using 6 µl blood on a 12 mm diameter template) and thin malaria films were prepared at each visit for Giemsa staining (3%, 40-50 min) according to procedures based on the Research Malaria Microscopy Standards.[13]

**All sites combined**	Readable slides with available data	4361	--
	Slides with asexual parasitaemia	2603	59.7%
	P. falciparum	112	2.6%
	P. falciparum - mixed	26	0.6%
	Non-*P. falciparum*	2465	56.5%
	Negative (no asexual parasitaemia)	1480	33.9%
	Accuracy (%)	4281/4361	98.6%
	Parasite density discordance	1625	62.4%
	**Overall sensitivity**	**2531/2603**	**97.2%**
	**Overall specificity**	**1471/1480**	**99.4%**
	**Kappa statistics for asexual parasite detection & species**	**0.93**	[0.92 - 0.94]
**AF001 Jalalabad**	Readable slides with available data	447	--
	Slides with asexual parasitaemia	223	49.9%
	P. falciparum	4	0.9%
	P. falciparum - mixed	2	0.4%
	Non-*P. falciparum*	217	48.5%
	Negative (no asexual parasitaemia)	224	50.1%
	Accuracy (%)	415/447	92.8%
	Parasite density discordance	140	62.8%
	**Overall sensitivity**	**192/223**	**86.1%**
	**Overall specificity**	**222/224**	**99.1%**
	**Kappa statistics for asexual parasite detection & species**	**0.82**	[0.76 - 0.87]
**AF008 Laghman**	Readable slides with available data	329	--
	Slides with asexual parasitaemia	197	59.9%
	P. falciparum	0	0.0%
	P. falciparum - mixed	0	0.0%

[13] WHO Special Programme for Research and Training in Tropical Diseases. Microscopy for the detection, identification and quantification of malaria parasites on stained thick and thin blood films in research settings, 2015. https://www.who.int/tdr/publications/microscopy_detec_ident_quantif/en/

	Non-*P. falciparum*	197	59.9%
	Negative (no asexual parasitaemia)	132	40.1%
	Accuracy (%)	320/329	97.3%
	Parasite density discordance	152	77.2%
	**Overall sensitivity**	**188/197**	**95.4%**
	**Overall specificity**	**132/132**	**100.0%**
	**Kappa statistics for asexual parasite detection & species**	**0.94**	[0.90 - 0.98]
**ET001 Arba Minch**	Readable slides with available data	826	--
	Slides with asexual parasitaemia	335	40.6%
	P. falciparum	15	1.8%
	P. falciparum - mixed	3	0.4%
	Non-*P. falciparum*	317	38.4%
	Negative (no asexual parasitaemia)	224	27.1%
	Accuracy (%)	817/826	99.0%
	Parasite density discordance	158	47.2%
	**Overall sensitivity**	**326/335**	**97.3%**
	**Overall specificity**	**224/224**	**100.0%**
	**Kappa statistics for asexual parasite detection & species**	**0.96**	[0.93 - 0.98]
**ET002 Metahara**	Readable slides with available data	340	--
	Slides with asexual parasitaemia	211	62.1%
	P. falciparum	5	1.5%
	P. falciparum - mixed	0	0.0%
	Non-*P. falciparum*	206	60.6%
	Negative (no asexual parasitaemia)	118	34.7%
	Accuracy (%)	332/340	97.6%
	Parasite density discordance	80	37.9%
	**Overall sensitivity**	**198/211**	**93.8%**
	**Overall specificity**	**118/118**	**100.0%**
	**Kappa statistics for asexual parasite detection & species**	**0.90**	[0.85 - 0.95]
**ID004 Hanura**	Readable slides with available data	1117	--
	Slides with asexual parasitaemia	716	64.1%
	P. falciparum	37	3.3%
	P. falciparum - mixed	10	0.9%
	Non-*P. falciparum*	669	59.9%
	Negative (no asexual parasitaemia)	401	35.9%
	Accuracy (%)	1107/1117	99.1%

	Parasite density discordance	500/716	69.8%
	**Overall sensitivity**	**709/716**	**99.0%**
	**Overall specificity**	**398/401**	**99.3%**
	**Kappa statistics for asexual parasite detection & species**	**0.92**	[0.90 - 0.94]
**ID005 Tanjung Leidong**	Readable slides with available data	630	--
	Slides with asexual parasitaemia	376	59.7%
	P. falciparum	0	0.0%
	P. falciparum - mixed	0	0.0%
	Non-*P. falciparum*	376	59.7%
	Negative (no asexual parasitaemia)	254	40.3%
	Accuracy (%)	616/630	97.8%
	Parasite density discordance	295/376	78.5%
	**Overall sensitivity**	**366/376**	**97.3%**
	**Overall specificity**	**250/254**	**98.4%**
	**Kappa statistics for asexual parasite detection & species**	**0.95**	[0.93 - 0.98]
**VN001 Dak O & Bu Gia Map**	Readable slides with available data	485	--
	Slides with asexual parasitaemia	295	60.8%
	P. falciparum	26	5.4%
	P. falciparum - mixed	3	0.6%
	Non-*P. falciparum*	266	54.8%
	Negative (no asexual parasitaemia)	190	39.2%
	Accuracy (%)	484/485	99.8%
	Parasite density discordance	25	8.5%
	**Overall sensitivity**	**295/295**	**100.0%**
	**Overall specificity**	**189/190**	**99.5%**
	**Kappa statistics for asexual parasite detection & species**	**0.96**	[0.94 - 0.98]
**VN002 Krong Pa**	Readable slides with available data	187	--
	Slides with asexual parasitaemia	109	58.3%
	P. falciparum	0	0.0%
	P. falciparum - mixed	0	0.0%
	Non-*P. falciparum*	109	58.3%
	Negative (no asexual parasitaemia)	78	41.7%
	Accuracy (%)	185/187	98.9%
	Parasite density discordance	27	24.8%
	**Overall sensitivity**	**107/109**	**98.2%**
	**Overall specificity**	**78/78**	**100.0%**
	**Kappa statistics for asexual parasite detection & species**	**0.98**	[0.95 - 1.00]

8. Statistical Methods

Full details of the Statistical Analysis Plan are available in the published protocol and associated supplementary files[14].

All statistical analyses were performed using Stata v14 (StataCorp, College Station, TX, USA).

Baseline characteristics

Patient and disease characteristics at baseline were summarised by study arm using means [SD], medians (25th – 75th percentile), geometric mean [SD (log scale), 95% reference range] and frequencies (proportion).

Summary of efficacy endpoints

Efficacy endpoints including early treatment failure (recurrent parasitaemia within 3 days), and recurrence within 28, 42, and 365 days were summarised by treatment arm. The primary efficacy endpoint, incidence rate of symptomatic *P. vivax* recurrence, was calculated as the number of symptomatic *P. vivax* recurrences per person-year, with censoring at 365 days. This approach was also followed for the incidence rate of any (and symptomatic) *P. falciparum* and *P. spp* infections, and any *P. vivax* recurrences. Incidence risks of *P. vivax* considered only the first *P. vivax* recurrence (regardless of symptoms) and were calculated using the Kaplan-Meier method with censoring at the time of failure or a non-vivax infection, or at 28 days, 42 days, or 365 days. The same approach was applied for the incidence risks of symptomatic *P.* vivax recurrence, any (and symptomatic) *P. falciparum* recurrence, and any (and symptomatic) *P. spp* recurrence. The proportion of patients with patent *P. vivax* parasitaemia or fever on day one, two, and three following the initial enrolment treatment on day 0, and the proportion of patients with patent *P. falciparum* parasitaemia or fever on day one, two, and three following an initial *P. falciparum* recurrence, was calculated by treatment arm.

Efficacy endpoints

To assess the primary efficacy endpoint for radical cure, the absolute incidence rate difference in symptomatic *P. vivax* recurrences (mono-infection or mixed) per person-year between the 7-day arm and the 14-day arm was estimated using weighted least squares regression with a robust standard error[15] [Xu et al. 2010], and a non-inferiority margin of 0.07 recurrences per person-year. To assess the secondary efficacy endpoints, incidence rate ratios were estimated using negative binomial regression (analysis of all recurrences), and cumulative incidence risks and hazard ratios were estimated using the Kaplan-Meier method and Cox regression (analysis of time to the first recurrence), with comparisons between the 7-day and control arms, the 14-day and control arms, and the 7-day and 14-day arms. The main analysis was done in the ITT (Intention to Treat) population; analyses were repeated in the PP (Per Protocol) population.

Further details on incidence rate analyses, including sensitivity analyses:

In the analysis of incidence rates (both differences and ratios), follow-up time began at enrolment and patients were censored at 365 days. The effect of post-treatment prophylaxis was assessed in a sensitivity

---

[14] Improv Study Group. Improving the radical cure of vivax malaria (IMPROV): a study protocol for a multicentre randomised, placebo-controlled comparison of short and long course primaquine regimens. *BMC Infect Dis,* 2015; **15**: 558

[15] Xu Y, Cheung YB, Lam KF*, et al.* A simple approach to the estimation of incidence rate difference. *Am J Epidemiol*. 2015; **172**(3):334-43.

analysis subtracting 28 days from a patient's follow-up time for each antimalarial treatment, irrespective of the drug. To determine the effect of providing open primaquine to all patients after the 3rd symptomatic *P. vivax* recurrence in Vietnam rather than after the 4th at other sites, two sensitivity analyses were performed – 1) excluding Vietnam; and 2) including Vietnam but censoring all patients at the time point of the 3rd recurrence at other sites.

Censoring

All analyses censored patients at the time of their last visit if they were lost to follow-up or if the intervention was discontinued. Patients were also censored from risk analyses on the day of initial parasitaemia (regardless of species or symptoms), on the day preceding a period of absence of 18 consecutive days prior to 42 days' follow-up, or two consecutive monthly visits after 42 days of follow-up. Additionally, schizontocidal efficacy analyses to 28 or 42 days censored patients at the day of the last blood smear prior to 25 or 39 days if no blood smear was taken between 25-31 or 39-45 days, respectively.

Heterogeneity between sites

For all efficacy endpoints within 365 days (radical cure), coefficients and their standard errors were first estimated within each study arm using the statistical methods described above (see **Efficacy Endpoints**) and heterogeneity was assessed visually using Forrest plots. Random effects meta-analysis was used to estimate the I^2 value for heterogeneity across sites, and the pooled measure of association across sites was presented unless there was substantial heterogeneity. There were too few events to assess heterogeneity between study sites in the analysis of secondary efficacy endpoints within 28 days and 42 days (schizontocidal efficacy).

Safety analysis - haemolysis

The incidence risk of severe anaemia or transfusion within 365 days of follow-up was calculated using the Kaplan-Meier method within each treatment arm. Patients were censored at the time of failure, at the time of the last visit, or at the last visit immediately prior to missing two or more scheduled haemoglobin measurements. There were too few outcomes for comparison between arms. The nadir of haemoglobin within 28 days and the time to nadir was summarised within treatment arms; patients who missed two or more consecutive scheduled haemoglobin measurements were excluded. Nadirs were compared between arms using linear regression adjusting for site. The median day of nadir was compared between study arms using a nonparametric K-sample test on the equality of medians. The mean and standard deviation of haemoglobin on day 0, 3, 7, and 13 (or 14 in the G6PD arm), and the mean [SD] change in haemoglobin from day 0 to days 3, 7, and 13 (or 14 in the G6PD arm), was calculated within each arm. Additionally, the proportion of patients whose haemoglobin dropped more than 5 g/dL or more than 25% from day 0 to days 3, 7, and 13 (or 14 in the G6PD arm) was calculated within each arm. Comparisons in mean changes between arms was done using linear-mixed effects modelling adjusting for site, with random effects for the intercept and slope, and an interaction parameter between the day of measurement and the study arm.

Safety analysis – adverse events

Adverse events (AEs) within 42 days of initial treatment were summarised within each treatment arm, by severity (only grade 3 and 4) and in relation to primaquine. The number of patients with AEs within 7, 14, 28, and 42 days of initial treatment was calculated within each arm, by relation to primaquine, to assess trends in the incidence of adverse events over time. All serious adverse events (SAEs) are reported and presented by relation to study drug and severity.

9. Consort Flow Chart

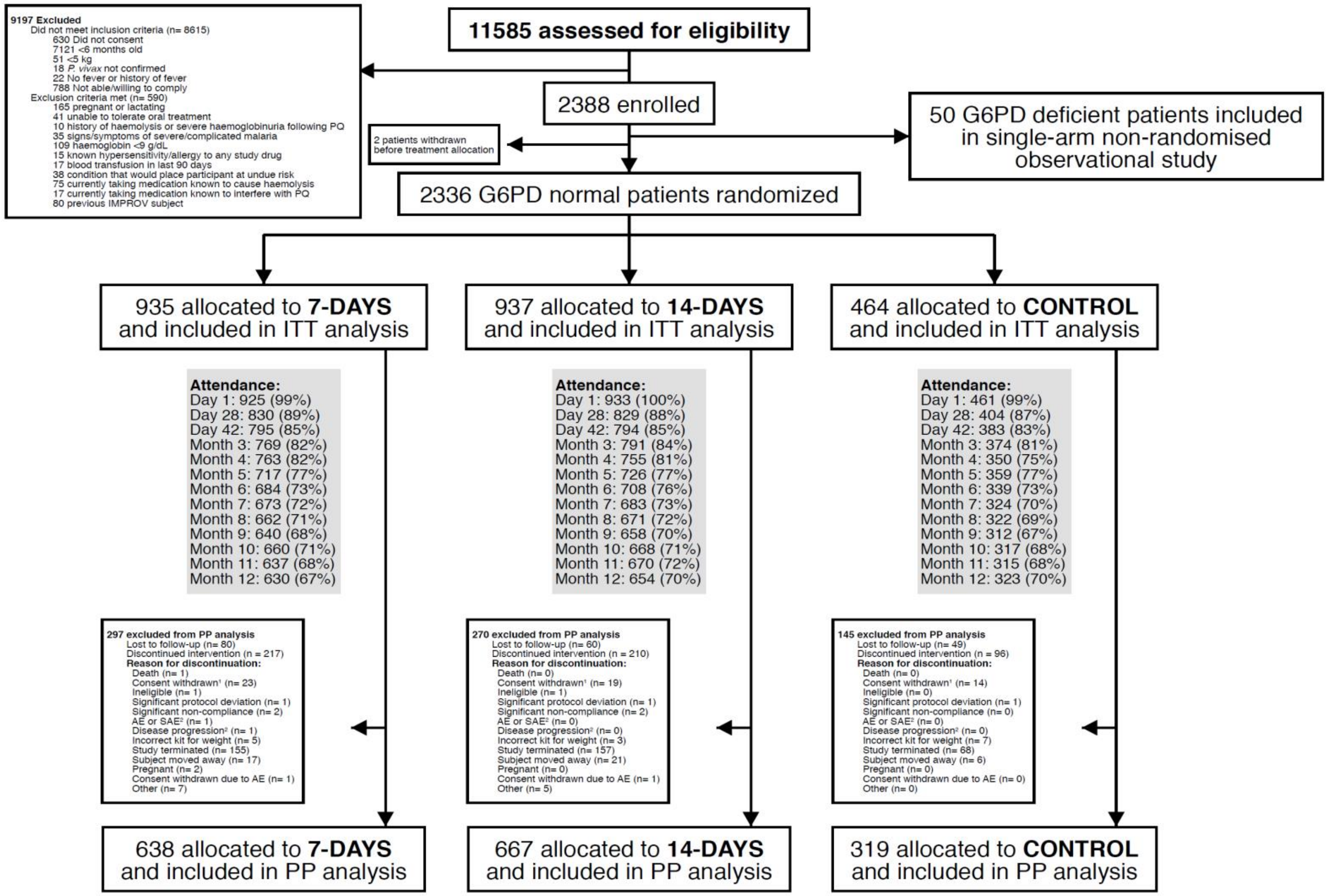

7-day arm: Primaquine/placebo 1.0 mg/kg/day x 7 days (7mg/kg total dose) followed by 7 days of primaquine placebo;
14-day arm: Primaquine/placebo 0.5 mg/kg/day x 14 days (7mg/kg total dose); Control arm: Primaquine placebo for 14 days;
AE: adverse event; SAE: serious adverse event; 1: Consent withdrawn due to reasons unrelated to an adverse event;
2: AE/SAE/Disease progression resulting in inability to comply with study procedures

10. Site-specific Baseline Tables

		Afghanistan		Ethiopia		Indonesia		Vietnam		Total
		AF001 Jalalabad	AF008 Laghman	ET001 Arba Minch	ET002 Metahara	ID004 Hanura	ID005 Tanjung Leidong	VN001 Dak O & Bu Gia Map	VN002 Krong Pa	
		N=311	N=120	N=371	N=209	N=575	N=425	N=219	N=106	N=2,336
**Age (years), median (IQR)**		14.0 (9.0 - 22.0)	11.0 (7.5 - 16.0)	16.0 (10.0 - 20.0)	16.0 (11.0 - 27.0)	14.0 (8.0 - 27.0)	17.0 (11.0 - 30.0)	22.0 (16.0 - 32.0)	25.0 (22.0 - 30.0)	16.0 (10.0 - 26.0)
**Age category N (%)**	6-12 months	1 (0.3%)	1 (0.8%)	0 (0.0%)	0 (0.0%)	1 (0.2%)	0 (0.0%)	0 (0.0%)	0 (0.0%)	3 (0.1%)
	>=1 & <5 yrs	22 (7.1%)	5 (4.2%)	43 (11.6%)	11 (5.3%)	50 (8.7%)	19 (4.5%)	1 (0.5%)	0 (0.0%)	151 (6.5%)
	>=5 & <15 yrs	135 (43.4%)	79 (65.8%)	119 (32.1%)	72 (34.4%)	264 (45.9%)	144 (33.9%)	40 (18.3%)	3 (2.8%)	856 (36.6%)
	>=15 yrs	153 (49.2%)	35 (29.2%)	209 (56.3%)	126 (60.3%)	260 (45.2%)	262 (61.6%)	178 (81.3%)	103 (97.2%)	1,326 (56.8%)
**Sex N (%)**	Male	234 (75.2%)	71 (59.2%)	195 (52.6%)	136 (65.1%)	300 (52.2%)	251 (59.1%)	184 (84.0%)	96 (90.6%)	1,467 (62.8%)
	Female	77 (24.8%)	49 (40.8%)	176 (47.4%)	73 (34.9%)	275 (47.8%)	174 (40.9%)	35 (16.0%)	10 (9.4%)	869 (37.2%)
**Weight (kg) median (IQR)**		41.0 (22.5 - 61.3)	31.9 (21.8 - 46.9)	47.0 (24.0 - 58.0)	47.1 (29.0 - 55.5)	38.0 (21.3 - 52.0)	49.0 (27.4 - 58.1)	52.0 (45.0 - 60.0)	55.0 (52.0 - 60.0)	46.6 (26.0 - 57.0)
**Weight category N (%)**	5-9.9 kg	2 (0.6%)	1 (0.8%)	7 (1.9%)	0 (0.0%)	5 (0.9%)	2 (0.5%)	0 (0.0%)	0 (0.0%)	17 (0.7%)
	10-22.9 kg	78 (25.1%)	34 (28.3%)	69 (18.6%)	28 (13.4%)	158 (27.5%)	69 (16.2%)	13 (5.9%)	0 (0.0%)	449 (19.2%)
	23-34.9 kg	55 (17.7%)	33 (27.5%)	47 (12.7%)	38 (18.2%)	95 (16.5%)	63 (14.8%)	14 (6.4%)	1 (0.9%)	346 (14.8%)
	35-45.9 kg	31 (10.0%)	21 (17.5%)	56 (15.1%)	32 (15.3%)	95 (16.5%)	47 (11.1%)	29 (13.2%)	9 (8.5%)	320 (13.7%)
	46+ kg	145 (46.6%)	31 (25.8%)	192 (51.8%)	111 (53.1%)	222 (38.6%)	244 (57.4%)	163 (74.4%)	96 (90.6%)	1,204 (51.5%)

P. vivax **parasites/uL - geometric mean (95% normal range)**		1347 (278 - 17500)	1830 (221 - 10935)	12090 (722 - 135000)	2237 (45 - 77500)	1379 (52 - 13833)	6334 (1615 - 17896)	7639 (749 - 32500)	6952 (593 - 52500)	3440 (140 - 53037)
**Gametocytes present N (%)**		311 (100.0%)	1 (0.8%)	371 (100.0%)	207 (99.0%)	574 (99.8%)	425 (100.0%)	219 (100.0%)	106 (100.0%)	2,214 (94.8%)
**Gametocytes /uL - geometric mean (95% normal range)**		441 (74-17500)	56 (. - .)	664 (56 - 3093)	499 (30 - 9701)	38 (3.7 - 307)	141 (30 - 578)	109 (15 - 734)	403 (19 - 15000)	201 (15 - 3037)
**Temperature (˚C) mean [SD]**		37.6 [0.8]	37.7 [0.8]	37.3 [1.1]	37.4 [0.8]	37.7 [1.4]	38.0 [1.2]	38.7 [1.0]	39.0 [0.9]	37.8 [1.2]
**Fever (Axillary >37.5C or Oral >38C) N (%)**		177 (59.4%)	72 (60.0%)	138 (37.2%)	107 (51.2%)	288 (50.1%)	271 (63.8%)	193 (88.1%)	96 (90.6%)	1,342 (57.8%)
**Haemoglobin (g/dL) mean [SD]**		13.3 [1.7]	12.5 [1.4]	13.2 [1.7]	13.0 [1.7]	12.4 [1.7]	12.9 [1.8]	13.5 [1.6]	13.6 [1.3]	13.0 [1.7]
**Anemia (Hb <10 g/dL) N (%)**		6 (1.9%)	4 (3.4%)	10 (2.7%)	4 (1.9%)	48 (8.3%)	15 (3.5%)	2 (0.9%)	0 (0.0%)	89 (3.8%)

11. Scatterplots of Total Dose of PQ Received by Weight

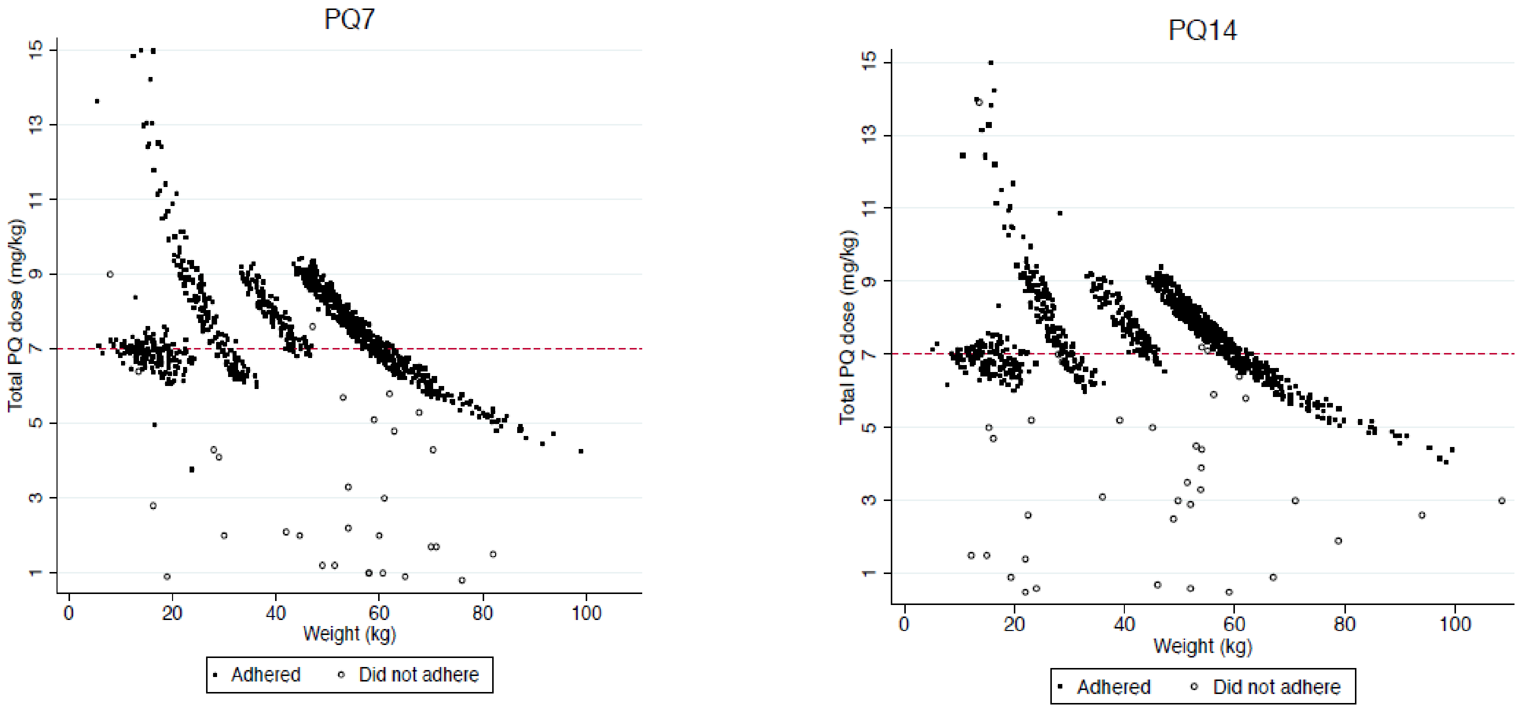

12. Adherence and Total Dose of Drug Received

	Control	PQ7	PQ14
	N=464	N=935	N=937
Number of patients (%) with incomplete treatment until day 7 [1,2]	11 (2.4%)	30 (3.2%)	19 (2.0%)
Number of patients (%) with incomplete treatment until day 14[1,3]	14 (3.0%)	53 (5.7%)	37 (3.9%)
Number of patients with interrupted treatment (> 1 day)[1]		125 (13.8%)	218 (24.2%)
Total Dose PQ received (mg/kg) – all patients[1]		7.39 [SD: 1.53] (range: 0.80 - 15.00)	7.39 [SD 1.48] (range: 0.50 - 15.00)
Total Dose PQ received (mg/kg) – only patients who received full course[1]		7.53 [SD: 1.28] (range: 3.80 - 15.00)	7.54 [SD: 1.19] (range: 4.20 - 15.00)
Total Dose PQ received (mg/kg) – only patients who received incomplete course[1, 2, 3]		3.16 [SD: 2.22], (range: 0.80- 9.00)	3.74 [SD 2.71] (range: 0.50-13.90)

[1] only recruitment episode
[2] incomplete treatment was defined as less than 7 doses
[3] incomplete treatment was defined as less than 14 doses

13. Parasite and Fever Clearance

	Control	PQ7	PQ14
	N=464	N=935	N=937
Asexual *P. vivax* parasitaemia on Day 1	231/461 (50.1%)	431/926 (46.5%)	426/934 (45.6%)
Asexual *P. vivax* parasitaemia on Day 2	24/458 (5.2%)	34/919 (3.7%)	35/933 (3.8%)
Asexual *P. vivax* parasitaemia on Day 3	1/457 (0.2%)	4/916 (0.4%)	3 /929(0.3%)
Fever on Day 1	30/460 (6.5%)	75/918 (8.2%)	59/931 (8.2%)
Fever on Day 2	5/458 (1.1%)	10/916 (1.1%)	5/931 (0.5%)
Fever on Day 3	3/451 (0.7%)	3/909 (0.3%)	4/919 (0.4%)

The denominator is the number of patients who attended on the respective day.
Patients with missing temperature were excluded from the fever denominators, but patients without a blood smear were not excluded from the parasitaemia denominators.
Fever was defined as either an axillary temperature >37.5°C or an oral temperature > 38.0 °C.

14. Histogram of the number of symptomatic *P. vivax* recurrences in each treatment arm

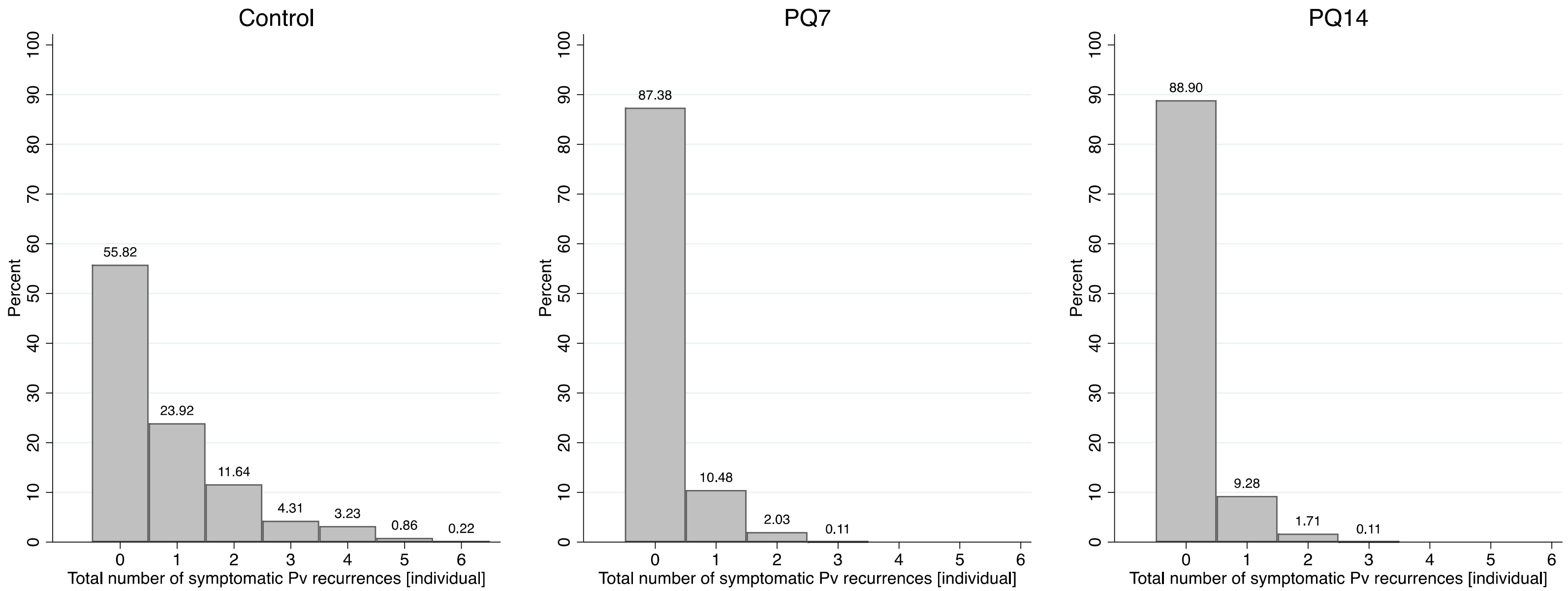

15. Site Specific Results for the Primary Endpoint – Incidence Rate of Recurrent Symptomatic Episodes of *P. vivax* within 12 months Follow up

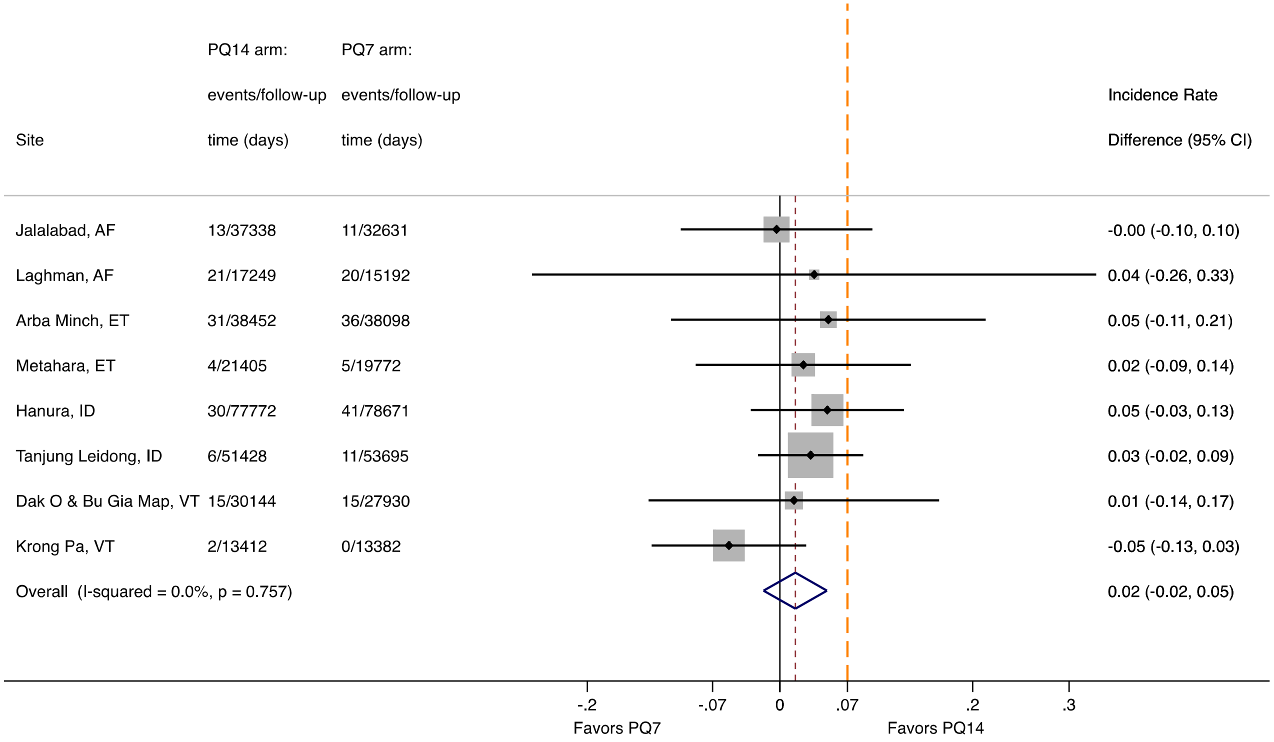

AF = Afghanistan; ET = Ethiopia; ID = Indonesia; VT = Vietnam

Absolute difference in incidence rates of symptomatic P. vivax between the 7-day and 14-day treatment arms: assessment of heterogeneity between study sites. The dashed orange line indicates the non-inferiority margin (incidence rate difference of 0.07 events per person-year). The incidence rate difference was estimated for each site using weighted least squares regression with a robust standard error; random effects meta-analysis was used to estimate the I^2 value for heterogeneity and the overall incidence rate difference across all sites.

16. Per protocol Analysis

Excludes patients who discontinued treatment and were lost to follow up.

Time point	Outcome	Measure of association	PQ7 vs Control	PQ14 vs Control	PQ7 vs PQ14
**12 months**	Symptomatic *P. vivax* parasitaemia	Pooled IRD*, (95%CI); p-value, [I^2]			0.02 (-0.02 - 0.05); 0.4162, [I^2 = 0.00]
		Pooled IRR, (95%CI); p-value, [I^2]	0.26 (0.20 - 0.35); <0.0001, [I^2 = 15.56]	0.23 (0.17 - 0.29); <0.0001, [I^2 = 5.18]	1.18 (0.90 - 1.55); 0.2320, [I^2 = 0.00]
		Pooled HR, (95%CI); p-value, [I^2]	0.22 (0.15 - 0.34); <0.0001, [I^2 = 39.11]	0.17 (0.11 - 0.26); <0.0001, [I^2 = 40.66]	1.19 (0.77 - 1.83); 0.4332, [I^2 = 26.55]
	Any *P. vivax* parasitaemia	Pooled IRR, (95%CI); p-value, [I^2]	0.24 (0.18 - 0.33); <0.0001, [I^2 = 32.50]	0.21 (0.15 - 0.29); <0.0001, [I^2 = 41.69]	1.15 (0.88 - 1.49); 0.2980, [I^2 = 0.00]
		Pooled HR, (95%CI); p-value, [I^2]	0.21 (0.14 - 0.31); <0.0001, [I^2 = 44.79]	0.17 (0.12 - 0.25); <0.0001, [I^2 = 38.48]	1.16 (0.86 - 1.58); 0.3399, [I^2 = 0.00]
	Symptomatic *P. falciparum* parasitaemia	Pooled IRR, (95%CI); p-value, [I^2]	1.08 (0.67 - 1.73); 0.7540, [I^2 = 0.00]	0.85 (0.53 - 1.38); 0.5180, [I^2 = 0.00]	1.24 (0.83 - 1.84); 0.2950, [I^2 = 0.00]
		Pooled HR, (95%CI); p-value, [I^2]	0.67 (0.38 - 1.20); 0.1814, [I^2 = 0.00]	0.56 (0.31 - 1.01); 0.0560, [I^2 = 0.00]	1.15 (0.71 - 1.86); 0.5589, [I^2 = 0.00]
	Any *P. falciparum* parasitaemia	Pooled IRR, (95%CI); p-value, [I^2]	1.09 (0.69 - 1.73); 0.7230, [I^2 = 0.00]	0.93 (0.58 - 1.48); 0.7560, [I^2 = 0.00]	1.14 (0.78 - 1.66); 0.5050, [I^2 = 0.00]
		Pooled HR, (95%CI); p-value, [I^2]	0.77 (0.46 - 1.29); 0.3292, [I^2 = 0.00]	0.65 (0.39 - 1.09); 0.1043, [I^2 = 0.00]	1.16 (0.76 - 1.75); 0.4970, [I^2 = 0.00]

*Non-Inferiority analysis
HR= Hazard ratio
IRR= Incidence rate ratio
IRD= Incidence rate difference

17. Sensitivity Analyses in the ITT Population

Exclusion of data from the Vietnam sites[1]					
**Time point**	**Outcome**	**Measure of association**	**PQ7 vs Control**	**PQ14 vs Control**	**PQ7 vs PQ14**
**12 months**	Symptomatic *P. vivax* parasitaemia	Pooled IRD*, (95%CI); p-value, [I^2]			0.03 (-0.01 - 0.07); 0.1031 [I^2 = 0.00]
		Pooled IRR, (95%CI); p-value, [I^2]	0.23 (0.17 - 0.30); <0.0001 [I^2 = 31.81]	0.19 (0.13 - 0.26); <0.0001 [I^2= 46.51]	1.24 (0.95 - 1.63); 0.1150 [I^2 = 0.00]
	Any *P.vivax* parasitaemia	Pooled IRR, (95%CI); p-value, [I^2]	0.21 (0.16 - 0.28); <0.0001 [I^2 = 33.21]	0.18 (0.13 - 0.25); <0.0001 [I^2 = 46.35]	1.20 (0.93 - 1.55); 0.1702 [I^2 = 0.00]
	Symptomatic *P. falciparum* parasitaemia	Pooled IRR, (95%CI); p-value, [I^2]	1.03 (0.65 - 1.64); 0.8882 [I^2 = 0.00]	0.72 (0.45 - 1.17); 0.1852 [I^2 = 0.00]	1.45 (0.97 - 2.15); 0.0703 [I^2 = 0.00]
	Any *P. falciparum* parasitaemia	Pooled IRR, (95%CI); p-value, [I^2]	1.12 (0.71 - 1.75); 0.6347 [I^2 = 0.00]	0.82 (0.51 - 1.30); 0.3935 [I^2 = 0.00]	1.39 (0.95 - 2.02); 0.0861 [I^2 = 0.00]
**Exclusion of data from the Indonesian sites[2]**					
**12 months**	Symptomatic *P. vivax* parasitaemia	Pooled IRD*, p-value (95%CI); p-value, [I^2]			-0.01 (-0.06 - 0.04); 0.7250 [I^2 = 0.00]
		Pooled IRR, (95%CI); p-value, [I^2]	0.18 (0.12 - 0.28); <0.0001 [I^2 = 48.36]	0.17 (0.11 - 0.26); <0.0001 [I^2 = 51.14]	1.07 (0.78 - 1.46); 0.6807 [I^2 = 0.00]
	Any *P.vivax* parasitaemia	Pooled IRR, (95%CI); p-value, [I^2]	0.17 (0.11 - 0.26); <0.0001 [I^2 = 57.05]	0.16 (0.10 - 0.24); <0.0001 [I^2 = 59.23]	1.07 (0.79 - 1.44); 0.6772 [I^2 = 0.00]
	Symptomatic *P. falciparum* parasitaemia	Pooled IRR, (95%CI); p-value, [I^2]	1.10 (0.62 - 1.96); 0.7495 [I^2 = 0.00]	0.81 (0.45 - 1.48); 0.5017 [I^2 = 0.00]	1.36 (0.85 - 2.18); 0.1986 [I^2 = 0.00]

	Any *P. falciparum* parasitaemia	Pooled IRR, (95%CI); p-value, [I^2]	1.23 (0.70 - 2.17); 0.4686 [I^2 = 0.00]	1.01 (0.57 - 1.79); 0.9661 [I^2 = 0.00]	1.22 (0.79 - 1.89); 0.3684 [I^2 = 0.00]
**Censoring patients after their 3rd Recurrence[3]**					
**12 months**	Symptomatic *P.vivax* parasitaemia	Pooled IRD*, (95%CI); p-value, [I^2]			0.02 (-0.02 - 0.05); 0.3396 [I^2 = 0.00]
		Pooled IRR, (95%CI); p-value, [I^2]	0.21 (0.15 - 0.29); <0.0001 [I^2 = 45.80]	0.18 (0.13 - 0.25); <0.0001 [I^2= 39.93]	1.19 (0.92 - 1.53); 0.1884 [I^2 = 0.00]
	Any *P.vivax* parasitaemia	Pooled IRR, (95%CI); p-value, [I^2]	0.20 (0.14 - 0.27); <0.0001 [I^2 = 49.64]	0.17 (0.12 - 0.23); <0.0001 [I^2 = 47.84]	1.17 (0.92 - 1.48); 0.2102 [I^2 = 0.00]
	Symptomatic *P. falciparum* parasitaemia	Pooled IRR, (95%CI); p-value, [I^2]	1.12 (0.70 - 1.81); 0.6386 [I^2 = 0.00]	0.92 (0.56 - 1.54); 0.7621 [I^2= 0.00]	1.28 (0.87 - 1.90); 0.2126 [I^2= 0.00]
	Any *P. falciparum* parasitaemia	Pooled IRR, (95%CI); p-value, [I^2]	1.06 (0.70 - 1.62); 0.7768 [I^2 = 0.00]	0.88 (0.56 - 1.37); 0.5645 [I^2 = 0.00]	1.24 (0.87 - 1.76); 0.2309 [I^2 = 0.00]
**Excluding the influence of post treatment prophylaxis [4]**					
**Time point**	**Outcome**	**Measure of association**			
**12 months**	Symptomatic *P. vivax* parasitaemia	Pooled IRD*, (95%CI); p-value, [I^2]			-0.01 (-0.06 - 0.04); 0.7250 [I^2 = 0.00]
		Pooled IRR, (95%CI); p-value, [I^2]	0.18 (0.12 - 0.28); <0.0001 [I^2 = 48.36]	0.17 (0.11 - 0.26); <0.0001 [I^2 = 51.14]	1.07 (0.78 - 1.46); 0.6807 [I^2 = 0.00]
	Any *P. vivax* parasitaemia	Pooled IRR, (95%CI); p-value, [I^2]	0.17 (0.11 - 0.26); <0.0001 [I^2 = 57.05]	0.16 (0.10 - 0.24); <0.0001 [I^2 = 59.23]	1.07 (0.79 - 1.44); 0.6772 [I^2 = 0.00]

	Symptomatic *P. falciparum* parasitaemia	Pooled IRR, (95%CI); p-value, [I^2]	1.10 (0.62 - 1.96); 0.7495 [I^2 = 0.00]	0.81 (0.45 - 1.48); 0.5017 [I^2 = 0.00]	1.36 (0.85 - 2.18); 0.1986 [I^2 = 0.00]
	Any *P. falciparum* parasitaemia	Pooled IRR, (95%CI); p-value, [I^2]	1.23 (0.70 - 2.17); 0.4686 [I^2 = 0.00]	1.01 (0.57 - 1.79); 0.9661 [I^2 = 0.00]	1.22 (0.79 - 1.89); 0.3684 [I^2 = 0.00]

* Non-inferiority analysis
[1] All sites except the sites in Vietnam provided the same treatment as originally randomized for up to 4 repeated episodes of *P. vivax* during follow up. The sites in Vietnam only provided the same treatment until the 3rd episode and thereafter treated patients with open primaquine for the subsequent episodes. This sensitivity analysis excludes the sites in Vietnam to accommodate for this difference in procedures.
[2] All sites except the sites in Indonesia used CQ as schizontocidal treatment, the Indonesian sites used DHAP, therefore this sensitivity analysis excludes the sites in Indonesia to accommodate for this difference in schizontocidal treatment regimen. This sensitivity analysis wasn't planned for a priori in the Statistical Analysis Plan, but added post hoc as it was perceived to be important additional information.
[3] All sites except the sites in Vietnam provided the same treatment as originally randomised for up to 4 repeated episodes of *P. vivax* during follow up. The sites in Vietnam only provided the same treatment until the 3rd episode and thereafter treated patients with open primaquine for the subsequent episodes. This sensitivity analysis includes all sites but censors all patients at the time point of the third episode in all countries to accommodate for this difference in procedures.
[4] The effect of post-treatment prophylaxis on the time of observation was assessed using sensitivity analyses by subtracting 28 days from a patient's follow-up time for each antimalarial treatment, irrespective of the drug.
IRR= Incidence rate ratio
IRD= Incidence rate difference

18. Site-Specific ITT Hazard Ratios (HR) and Incidence Rate Ratios (IRR)

The hazard ratios (HRs) were estimated using Cox regression (analysis of time to the first event). Incidence rate ratios (IRRs) were estimated using negative binomial regression (analysis of all events). Random effects meta-analysis was used to estimate the I^2 value for heterogeneity across sites. The pooled measure of association is presented across all sites.

a) <u>PQ7 vs PQ14:</u> Risks and rates for recurrent parasitemia due to symptomatic and any infection due to *P. vivax*

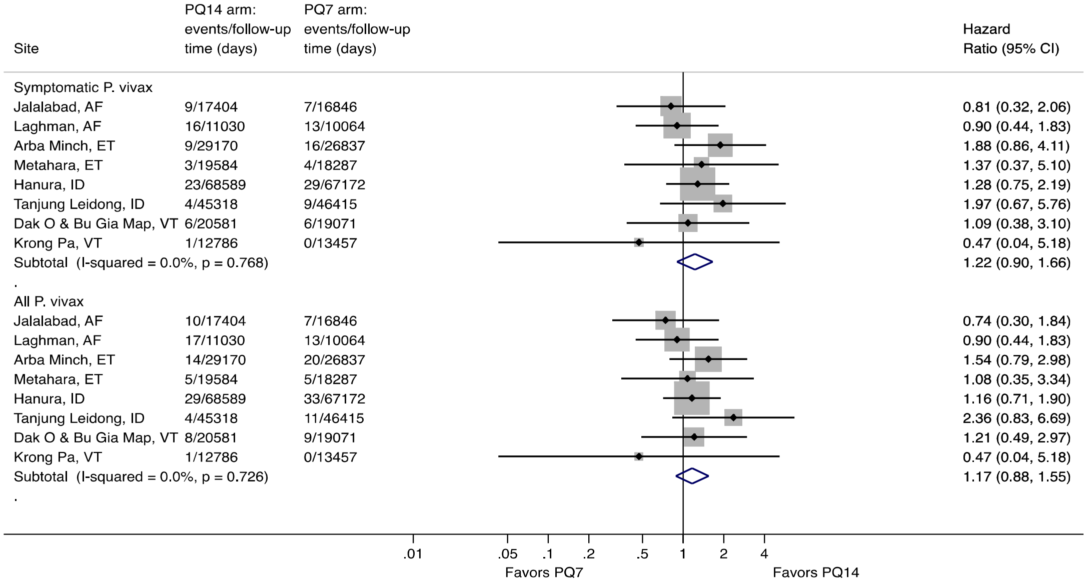

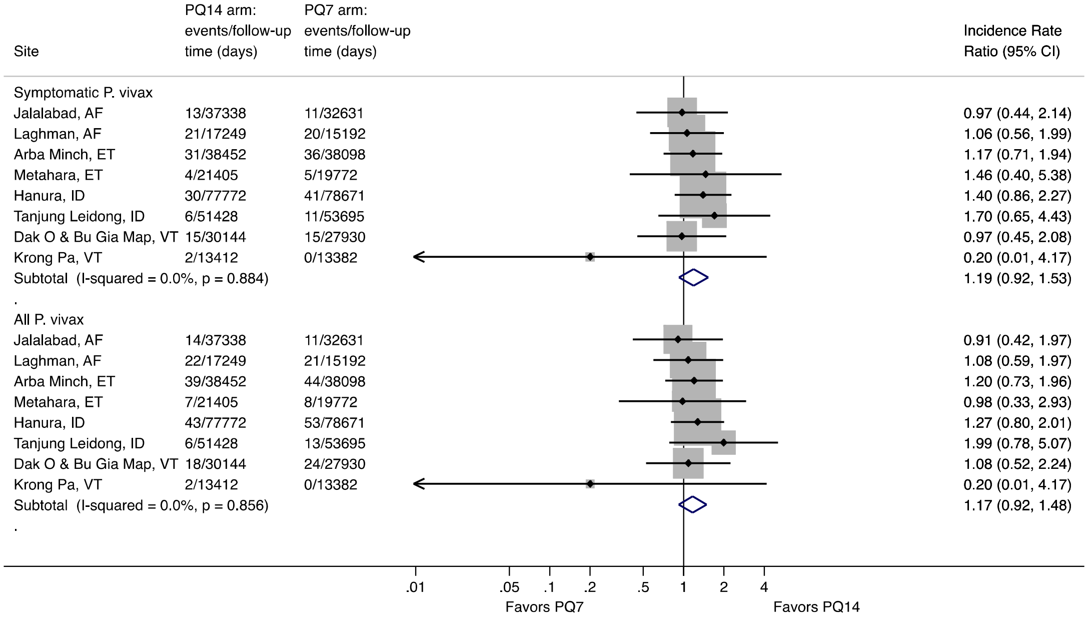

AF = Afghanistan; ET = Ethiopia; ID = Indonesia; VT = Vietnam

b) <u>PQ7 vs PQ14:</u> Risks and rates for recurrent parasitemia due to symptomatic and any infection due to *P. falciparum*

Site	PQ14 arm: events/follow-up time (days)	PQ7 arm: events/follow-up time (days)	Hazard Ratio (95% CI)
Symptomatic P. falciparum			
Jalalabad, AF	0/17404	0/16846	0.94 (0.06, 15.10)
Laghman, AF	0/11030	0/10064	0.96 (0.06, 15.37)
Arba Minch, ET	8/29170	17/26837	2.11 (0.95, 4.71)
Metahara, ET	1/19584	1/18287	1.10 (0.16, 7.84)
Hanura, ID	14/68589	17/67172	1.22 (0.62, 2.42)
Tanjung Leidong, ID	0/45318	0/46415	0.99 (0.06, 15.86)
Dak O & Bu Gia Map, VT	3/20581	4/19071	1.34 (0.36, 4.98)
Krong Pa, VT	0/12786	0/13457	0.95 (0.06, 15.12)
Subtotal (I-squared = 0.0%, p = 0.982)			1.42 (0.91, 2.22)
.			
All P. falciparum			
Jalalabad, AF	0/17404	2/16846	2.95 (0.31, 28.33)
Laghman, AF	0/11030	0/10064	0.96 (0.06, 15.37)
Arba Minch, ET	16/29170	25/26837	1.62 (0.88, 2.99)
Metahara, ET	3/19584	1/18287	0.53 (0.10, 2.91)
Hanura, ID	19/68589	23/67172	1.21 (0.67, 2.20)
Tanjung Leidong, ID	0/45318	0/46415	0.99 (0.06, 15.86)
Dak O & Bu Gia Map, VT	4/20581	4/19071	1.07 (0.31, 3.69)
Krong Pa, VT	1/12786	0/13457	0.48 (0.04, 5.31)
Subtotal (I-squared = 0.0%, p = 0.890)			1.28 (0.88, 1.86)
.			

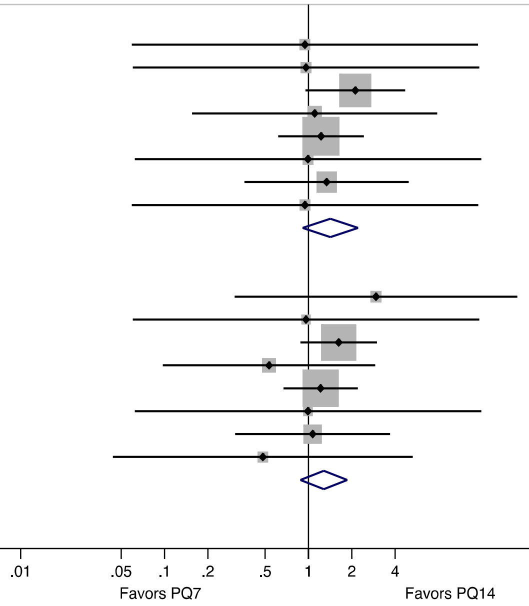

Site	PQ14 arm: events/follow-up time (days)	PQ7 arm: events/follow-up time (days)	Incidence Rate Ratio (95% CI)
Symptomatic P. falciparum			
Jalalabad, AF	0/37338	0/32631	1.14 (0.07, 18.27)
Laghman, AF	0/17249	0/15192	1.13 (0.07, 18.11)
Arba Minch, ET	18/38452	29/38098	1.62 (0.88, 2.96)
Metahara, ET	1/21405	1/19772	1.08 (0.15, 7.67)
Hanura, ID	20/77772	31/78671	1.38 (0.78, 2.47)
Tanjung Leidong, ID	0/51428	0/53695	0.96 (0.06, 15.32)
Dak O & Bu Gia Map, VT	13/30144	18/27930	1.13 (0.45, 2.84)
Krong Pa, VT	1/13412	0/13382	0.50 (0.05, 5.53)
Subtotal (I-squared = 0.0%, p = 0.989)			1.36 (0.95, 1.94)
.			
All P. falciparum			
Jalalabad, AF	0/37338	2/32631	3.43 (0.36, 32.96)
Laghman, AF	1/17249	0/15192	0.57 (0.05, 6.24)
Arba Minch, ET	29/38452	52/38098	1.63 (0.92, 2.89)
Metahara, ET	4/21405	1/19772	0.53 (0.10, 2.89)
Hanura, ID	26/77772	41/78671	1.33 (0.76, 2.32)
Tanjung Leidong, ID	0/51428	0/53695	0.96 (0.06, 15.32)
Dak O & Bu Gia Map, VT	20/30144	21/27930	0.84 (0.35, 1.98)
Krong Pa, VT	2/13412	0/13382	0.48 (0.04, 5.26)
Subtotal (I-squared = 0.0%, p = 0.718)			1.25 (0.89, 1.76)
.			

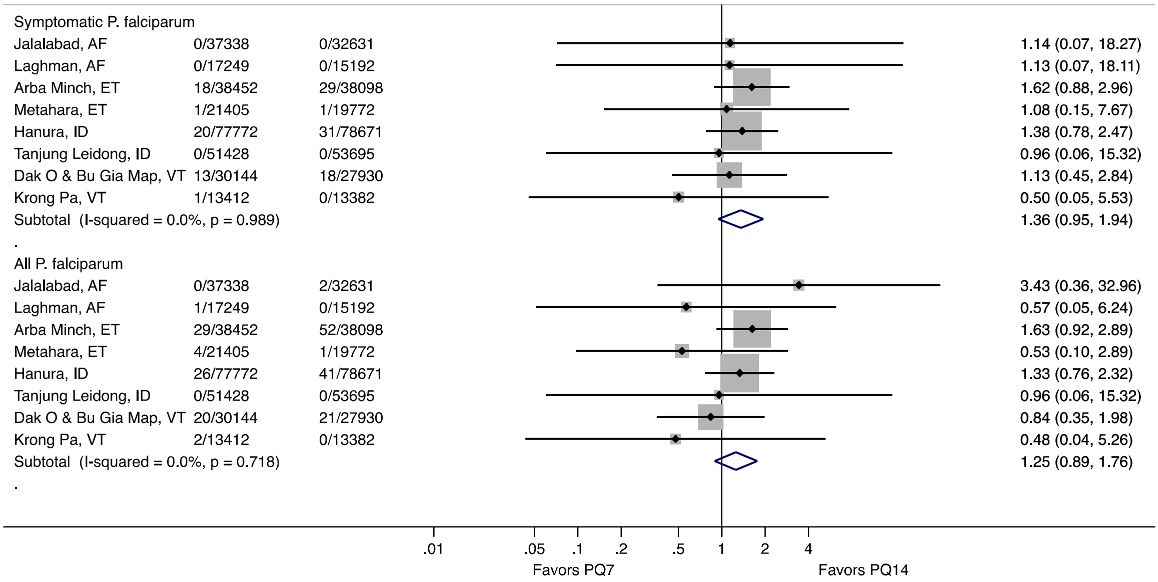

AF = Afghanistan; ET = Ethiopia; ID = Indonesia; VT = Vietnam

c) PQ7 vs Pq14: Risks and rates for recurrent parasitemia due to symptomatic and any infection due to all *Species*

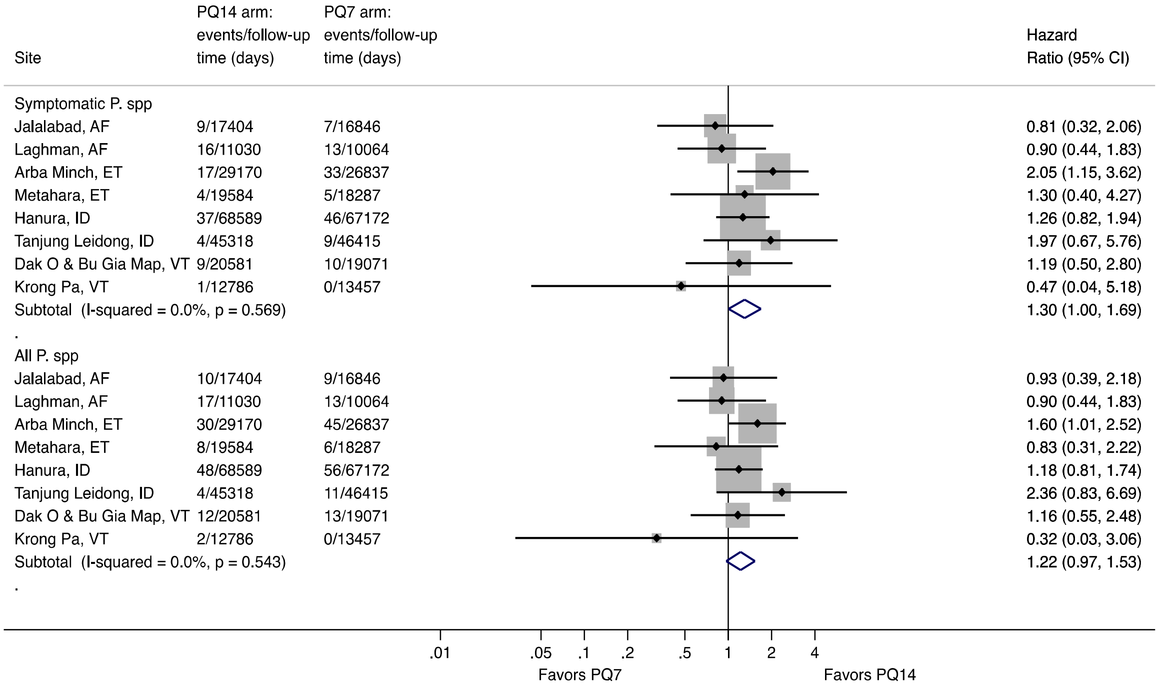

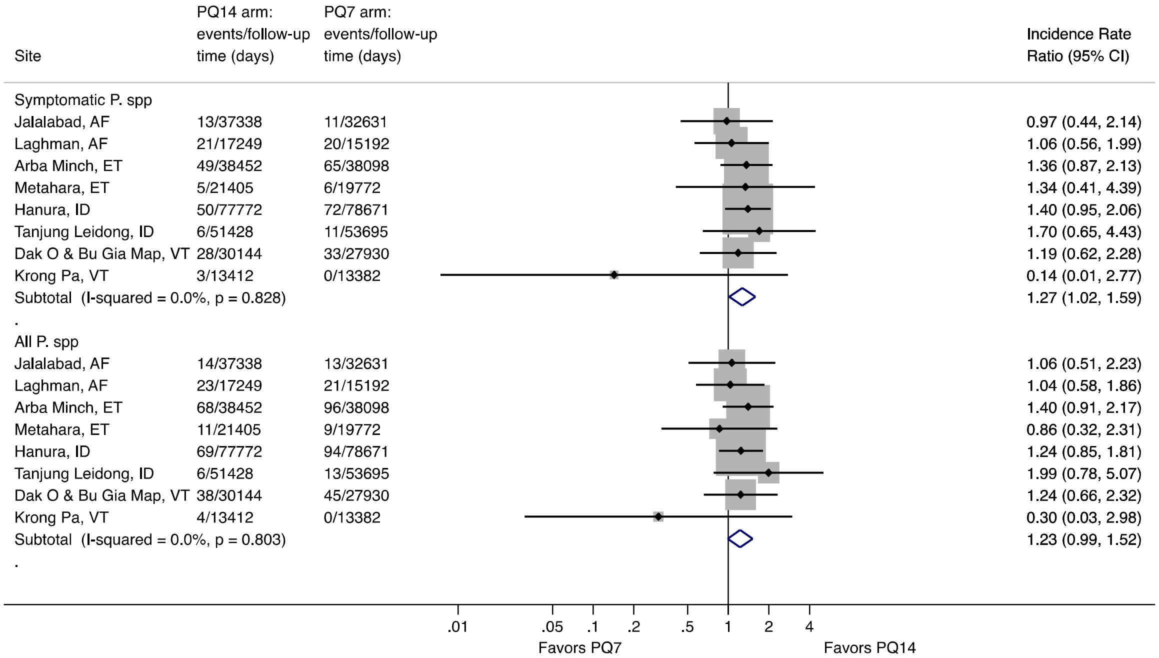

AF = Afghanistan; ET = Ethiopia; ID = Indonesia; VT = Vietnam

d) <u>PQ7 vs Control</u>: Risks and rates for recurrent parasitemia due to symptomatic and any infection due to *P. vivax*

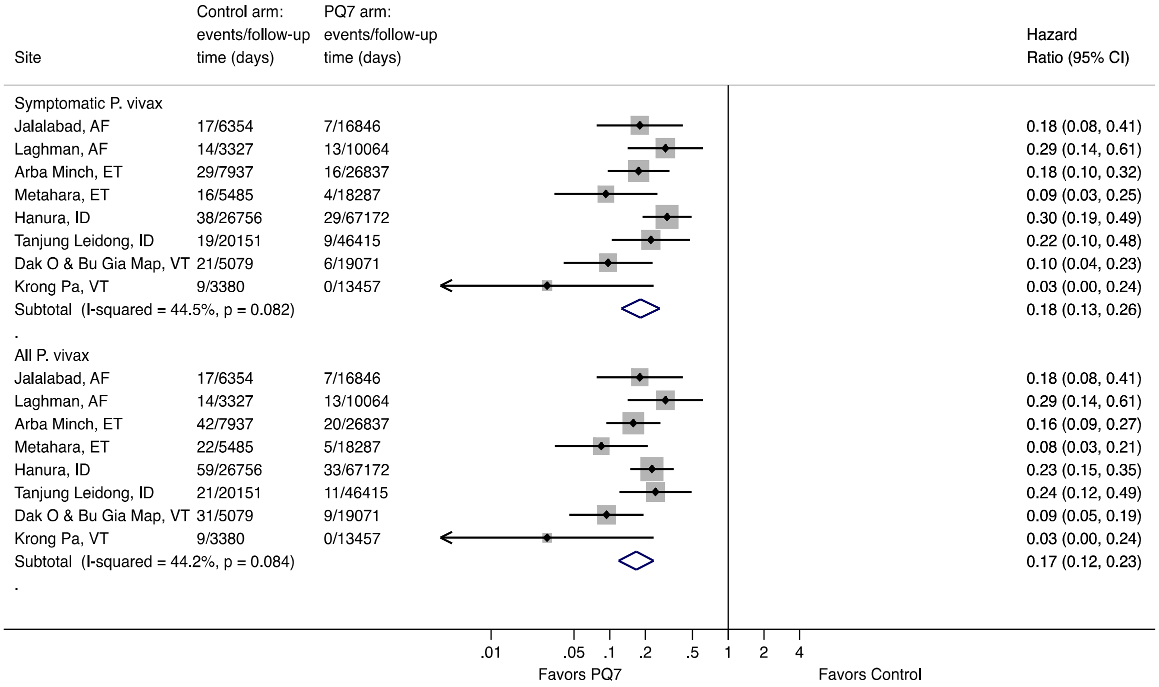

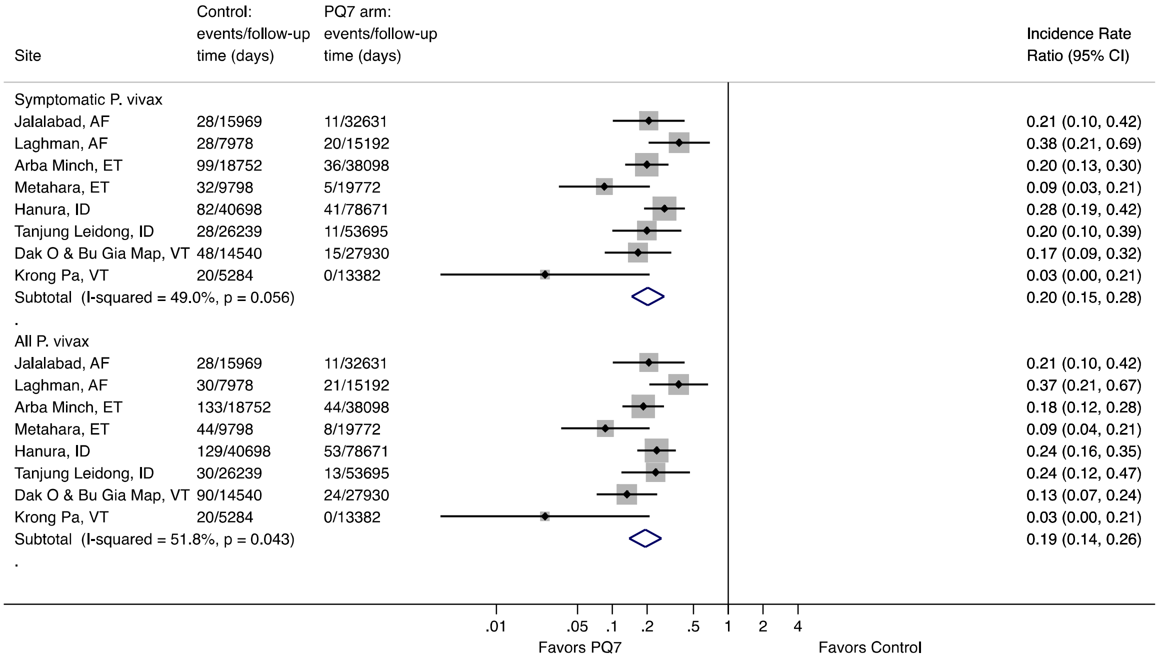

AF = Afghanistan; ET = Ethiopia; ID = Indonesia; VT = Vietnam

e) PQ7 vs Control: Risks and rates for recurrent parasitemia due to symptomatic and any infection due to *P. falciparum*

Site	Control arm: events/follow-up time (days)	PQ7 arm: events/follow-up time (days)	Hazard Ratio (95% CI)
Symptomatic P. falciparum			
Jalalabad, AF	0/6354	0/16846	0.31 (0.02, 4.89)
Laghman, AF	0/3327	0/10064	0.27 (0.02, 4.30)
Arba Minch, ET	5/7937	17/26837	1.29 (0.51, 3.27)
Metahara, ET	0/5485	1/18287	0.68 (0.06, 7.62)
Hanura, ID	8/26756	17/67172	0.83 (0.37, 1.86)
Tanjung Leidong, ID	0/20151	0/46415	0.39 (0.02, 6.16)
Dak O & Bu Gia Map, VT	0/5079	4/19071	0.77 (0.09, 6.60)
Krong Pa, VT	0/3380	0/13457	0.16 (0.01, 2.59)
Subtotal (I-squared = 0.0%, p = 0.820)			0.80 (0.47, 1.36)
.			
All P. falciparum			
Jalalabad, AF	0/6354	2/16846	1.19 (0.12, 11.59)
Laghman, AF	0/3327	0/10064	0.27 (0.02, 4.30)
Arba Minch, ET	7/7937	25/26837	1.38 (0.62, 3.06)
Metahara, ET	0/5485	1/18287	0.68 (0.06, 7.62)
Hanura, ID	9/26756	23/67172	1.05 (0.50, 2.19)
Tanjung Leidong, ID	0/20151	0/46415	0.39 (0.02, 6.16)
Dak O & Bu Gia Map, VT	1/5079	4/19071	0.39 (0.08, 2.02)
Krong Pa, VT	0/3380	0/13457	0.16 (0.01, 2.59)
Subtotal (I-squared = 0.0%, p = 0.674)			0.93 (0.58, 1.49)
.			

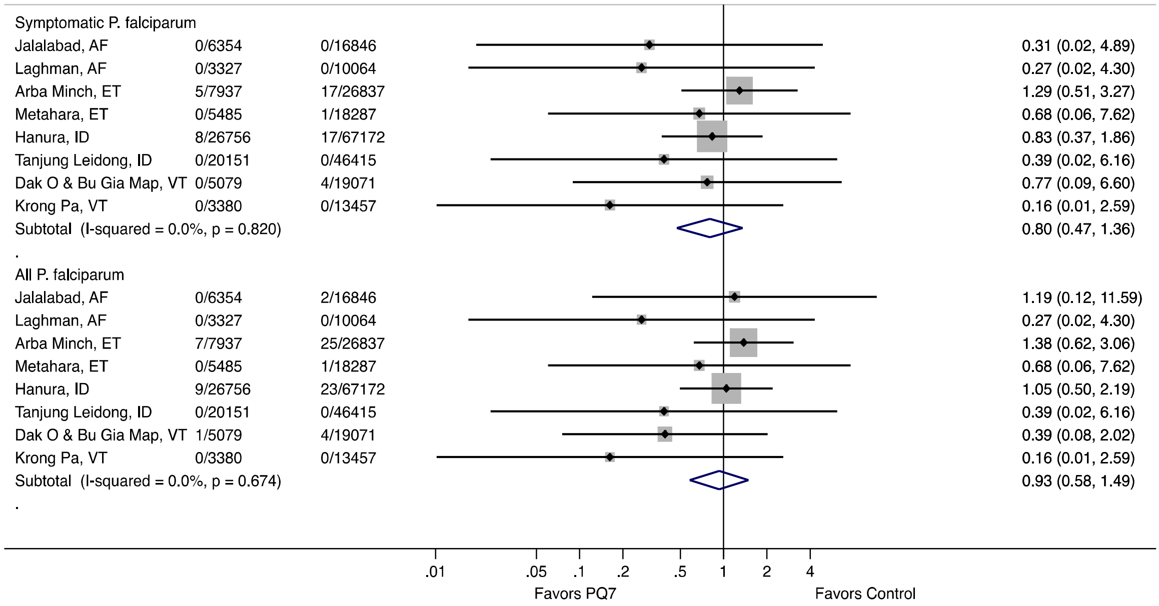

Site	Control: events/follow-up time (days)	PQ7 arm: events/follow-up time (days)	Incidence Rate Ratio (95% CI)
Symptomatic P. falciparum			
Jalalabad, AF	0/15969	0/32631	0.50 (0.03, 7.91)
Laghman, AF	0/7978	0/15192	0.54 (0.03, 8.57)
Arba Minch, ET	8/18752	29/38098	1.62 (0.76, 3.44)
Metahara, ET	1/9798	1/19772	0.50 (0.07, 3.58)
Hanura, ID	13/40698	31/78671	1.09 (0.57, 2.12)
Tanjung Leidong, ID	0/26239	0/53695	0.49 (0.03, 7.87)
Dak O & Bu Gia Map, VT	4/14540	18/27930	1.65 (0.45, 6.07)
Krong Pa, VT	0/5284	0/13382	0.41 (0.03, 6.57)
Subtotal (I-squared = 0.0%, p = 0.855)			1.16 (0.75, 1.78)
.			
All P. falciparum			
Jalalabad, AF	0/15969	2/32631	1.49 (0.15, 14.28)
Laghman, AF	0/7978	0/15192	0.54 (0.03, 8.57)
Arba Minch, ET	11/18752	52/38098	1.76 (0.84, 3.71)
Metahara, ET	1/9798	1/19772	0.50 (0.07, 3.58)
Hanura, ID	16/40698	41/78671	1.09 (0.57, 2.06)
Tanjung Leidong, ID	0/26239	0/53695	0.49 (0.03, 7.87)
Dak O & Bu Gia Map, VT	10/14540	21/27930	1.59 (0.43, 5.87)
Krong Pa, VT	0/5284	0/13382	0.41 (0.03, 6.57)
Subtotal (I-squared = 0.0%, p = 0.848)			1.22 (0.80, 1.85)
.			

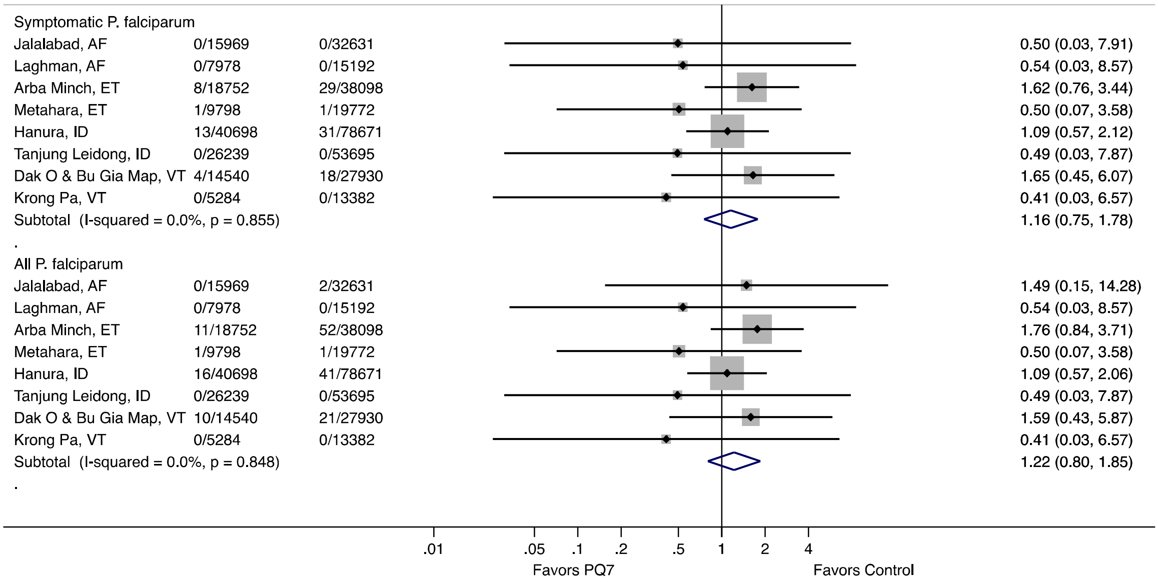

AF = Afghanistan; ET = Ethiopia; ID = Indonesia; VT = Vietnam

f) <u>PQ7 vs Control</u>: Risks and rates for recurrent parasitemia due to symptomatic and any infection due to all *species*

Site	Control arm: events/follow-up time (days)	PQ7 arm: events/follow-up time (days)	Hazard Ratio (95% CI)
Symptomatic P. spp			
Jalalabad, AF	17/6354	7/16846	0.18 (0.08, 0.41)
Laghman, AF	14/3327	13/10064	0.29 (0.14, 0.61)
Arba Minch, ET	34/7937	33/26837	0.34 (0.21, 0.55)
Metahara, ET	16/5485	5/18287	0.11 (0.04, 0.29)
Hanura, ID	46/26756	46/67172	0.40 (0.27, 0.61)
Tanjung Leidong, ID	19/20151	9/46415	0.22 (0.10, 0.48)
Dak O & Bu Gia Map, VT	21/5079	10/19071	0.14 (0.07, 0.29)
Krong Pa, VT	9/3380	0/13457	0.03 (0.00, 0.24)
Subtotal (I-squared = 57.8%, p = 0.020)			0.22 (0.15, 0.33)
.			
All P. spp			
Jalalabad, AF	17/6354	9/16846	0.23 (0.10, 0.50)
Laghman, AF	14/3327	13/10064	0.29 (0.14, 0.61)
Arba Minch, ET	49/7937	45/26837	0.33 (0.22, 0.49)
Metahara, ET	22/5485	6/18287	0.10 (0.04, 0.24)
Hanura, ID	68/26756	56/67172	0.34 (0.24, 0.48)
Tanjung Leidong, ID	21/20151	11/46415	0.24 (0.12, 0.49)
Dak O & Bu Gia Map, VT	32/5079	13/19071	0.12 (0.06, 0.22)
Krong Pa, VT	9/3380	0/13457	0.03 (0.00, 0.24)
Subtotal (I-squared = 62.4%, p = 0.009)			0.22 (0.15, 0.31)
.			

.01 .05 .1 .2 .5 1 2 4

Favors PQ7 Favors Control

Site	Control: events/follow-up time (days)	PQ7 arm: events/follow-up time (days)	Incidence Rate Ratio (95% CI)
Symptomatic P. spp			
Jalalabad, AF	28/15969	11/32631	0.21 (0.10, 0.42)
Laghman, AF	28/7978	20/15192	0.38 (0.21, 0.69)
Arba Minch, ET	107/18752	65/38098	0.30 (0.21, 0.43)
Metahara, ET	33/9798	6/19772	0.10 (0.04, 0.23)
Hanura, ID	95/40698	72/78671	0.40 (0.28, 0.56)
Tanjung Leidong, ID	28/26239	11/53695	0.20 (0.10, 0.39)
Dak O & Bu Gia Map, VT	52/14540	33/27930	0.29 (0.16, 0.51)
Krong Pa, VT	20/5284	0/13382	0.03 (0.00, 0.21)
Subtotal (I-squared = 60.3%, p = 0.014)			0.25 (0.18, 0.35)
.			
All P. spp			
Jalalabad, AF	28/15969	13/32631	0.24 (0.12, 0.47)
Laghman, AF	30/7978	21/15192	0.37 (0.21, 0.67)
Arba Minch, ET	144/18752	96/38098	0.30 (0.21, 0.43)
Metahara, ET	45/9798	9/19772	0.10 (0.04, 0.23)
Hanura, ID	145/40698	94/78671	0.33 (0.23, 0.45)
Tanjung Leidong, ID	30/26239	13/53695	0.24 (0.12, 0.47)
Dak O & Bu Gia Map, VT	100/14540	45/27930	0.21 (0.13, 0.36)
Krong Pa, VT	20/5284	0/13382	0.03 (0.00, 0.21)
Subtotal (I-squared = 50.9%, p = 0.047)			0.25 (0.18, 0.33)
.			

.01 .05 .1 .2 .5 1 2 4

Favors PQ7 Favors Control

AF = Afghanistan; ET = Ethiopia; ID = Indonesia; VT = Vietnam

g) <u>PQ14 vs Control</u>: Risks and rates for recurrent parasitemia due to symptomatic and any infection due *P. vivax*

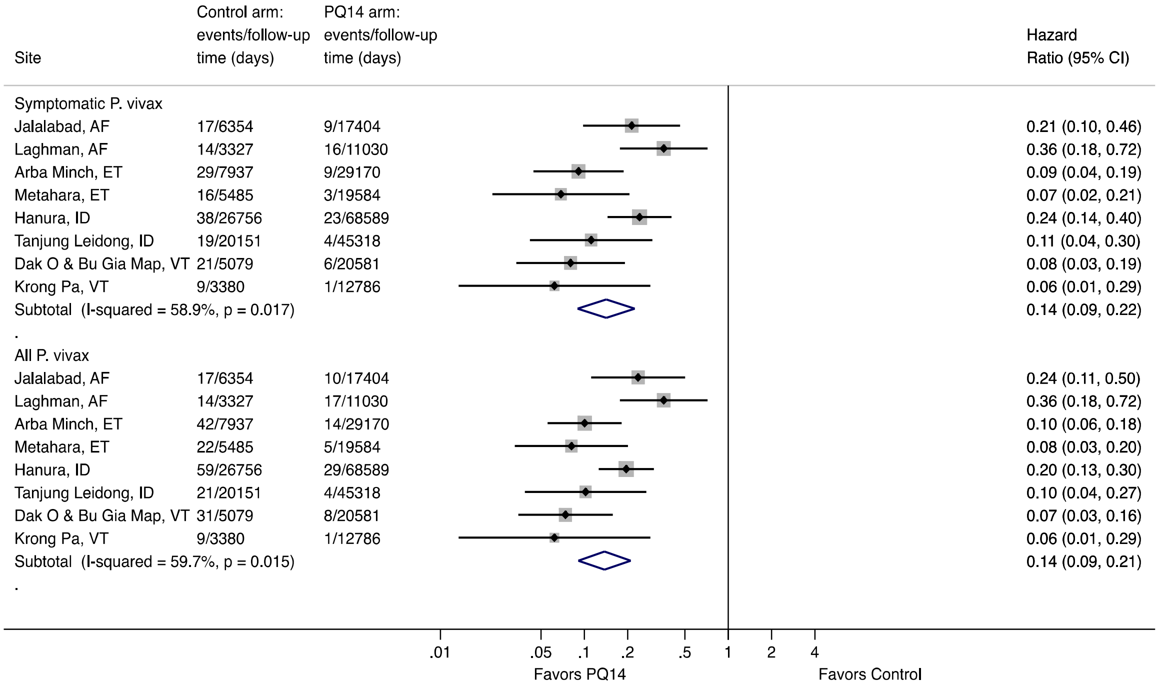

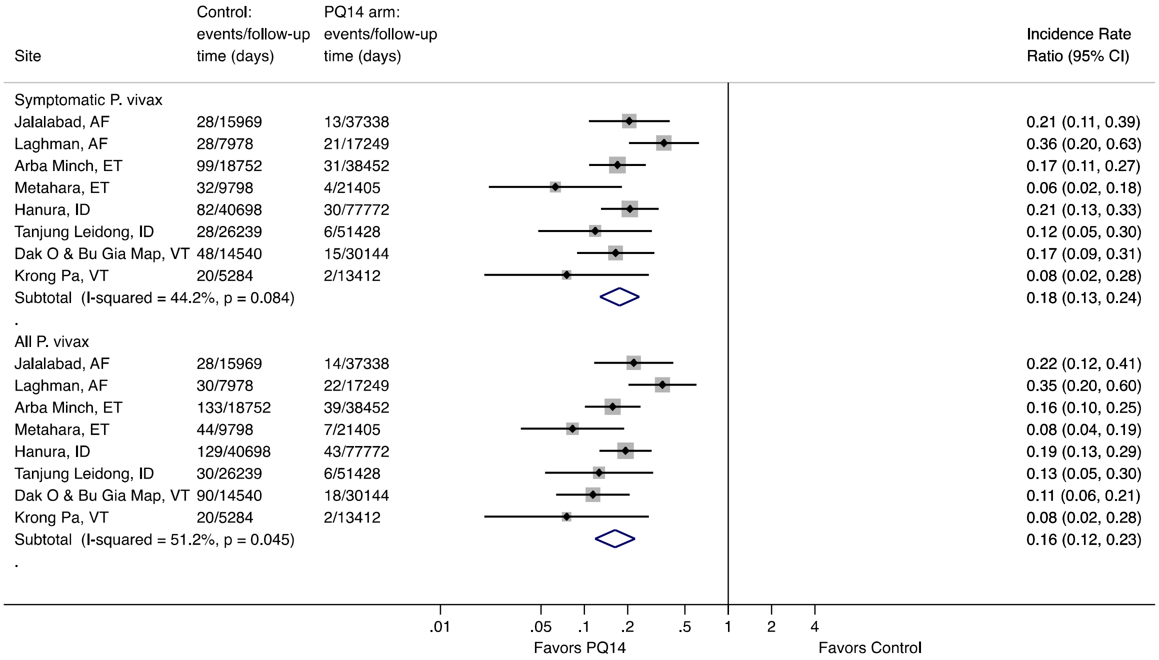

AF = Afghanistan; ET = Ethiopia; ID = Indonesia; VT = Vietnam

h) PQ14 vs Control: Risks and rates for recurrent parasitemia due to symptomatic and any infection due *P. falciparum*

Site	Control arm: events/follow-up time (days)	PQ14 arm: events/follow-up time (days)	Hazard Ratio (95% CI)
Symptomatic P. falciparum			
Jalalabad, AF	0/6354	0/17404	0.32 (0.02, 5.17)
Laghman, AF	0/3327	0/11030	0.28 (0.02, 4.48)
Arba Minch, ET	5/7937	8/29170	0.52 (0.18, 1.48)
Metahara, ET	0/5485	1/19584	0.52 (0.05, 5.80)
Hanura, ID	8/26756	14/68589	0.63 (0.27, 1.45)
Tanjung Leidong, ID	0/20151	0/45318	0.39 (0.02, 6.21)
Dak O & Bu Gia Map, VT	0/5079	3/20581	0.69 (0.07, 6.32)
Krong Pa, VT	0/3380	0/12786	0.17 (0.01, 2.74)
Subtotal (I-squared = 0.0%, p = 0.990)			0.52 (0.30, 0.91)
.			
All P. falciparum			
Jalalabad, AF	0/6354	0/17404	0.32 (0.02, 5.17)
Laghman, AF	0/3327	0/11030	0.28 (0.02, 4.48)
Arba Minch, ET	7/7937	16/29170	0.78 (0.33, 1.83)
Metahara, ET	0/5485	3/19584	1.15 (0.13, 10.37)
Hanura, ID	9/26756	19/68589	0.82 (0.38, 1.75)
Tanjung Leidong, ID	0/20151	0/45318	0.39 (0.02, 6.21)
Dak O & Bu Gia Map, VT	1/5079	4/20581	0.41 (0.08, 2.15)
Krong Pa, VT	0/3380	1/12786	0.51 (0.04, 6.04)
Subtotal (I-squared = 0.0%, p = 0.972)			0.70 (0.43, 1.13)
.			

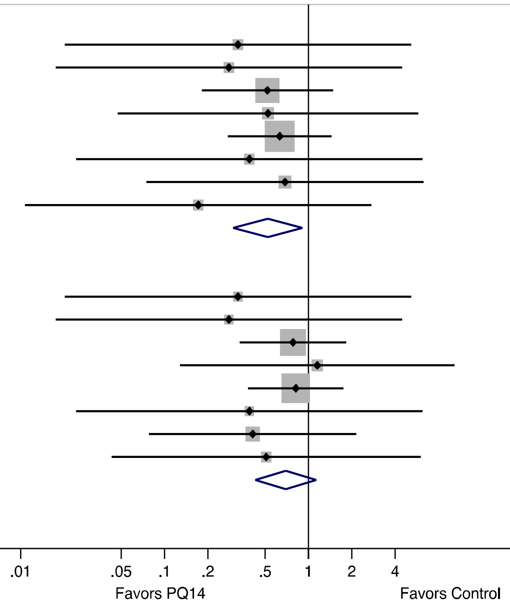

Site	Control: events/follow-up time (days)	PQ14 arm: events/follow-up time (days)	Incidence Rate Ratio (95% CI)
Symptomatic P. falciparum			
Jalalabad, AF	0/15969	0/37338	0.43 (0.03, 6.93)
Laghman, AF	0/7978	0/17249	0.47 (0.03, 7.57)
Arba Minch, ET	8/18752	18/38452	1.00 (0.44, 2.28)
Metahara, ET	1/9798	1/21405	0.47 (0.07, 3.31)
Hanura, ID	13/40698	20/77772	0.79 (0.40, 1.56)
Tanjung Leidong, ID	0/26239	0/51428	0.51 (0.03, 8.21)
Dak O & Bu Gia Map, VT	4/14540	13/30144	1.47 (0.40, 5.39)
Krong Pa, VT	0/5284	1/13412	0.82 (0.07, 9.04)
Subtotal (I-squared = 0.0%, p = 0.972)			0.85 (0.55, 1.33)
.			
All P. falciparum			
Jalalabad, AF	0/15969	0/37338	0.43 (0.03, 6.93)
Laghman, AF	0/7978	1/17249	0.95 (0.09, 10.45)
Arba Minch, ET	11/18752	29/38452	1.09 (0.50, 2.40)
Metahara, ET	1/9798	4/21405	0.96 (0.17, 5.21)
Hanura, ID	16/40698	26/77772	0.82 (0.42, 1.59)
Tanjung Leidong, ID	0/26239	0/51428	0.51 (0.03, 8.21)
Dak O & Bu Gia Map, VT	10/14540	20/30144	1.91 (0.54, 6.75)
Krong Pa, VT	0/5284	2/13412	0.86 (0.08, 9.50)
Subtotal (I-squared = 0.0%, p = 0.961)			0.97 (0.63, 1.49)
.			

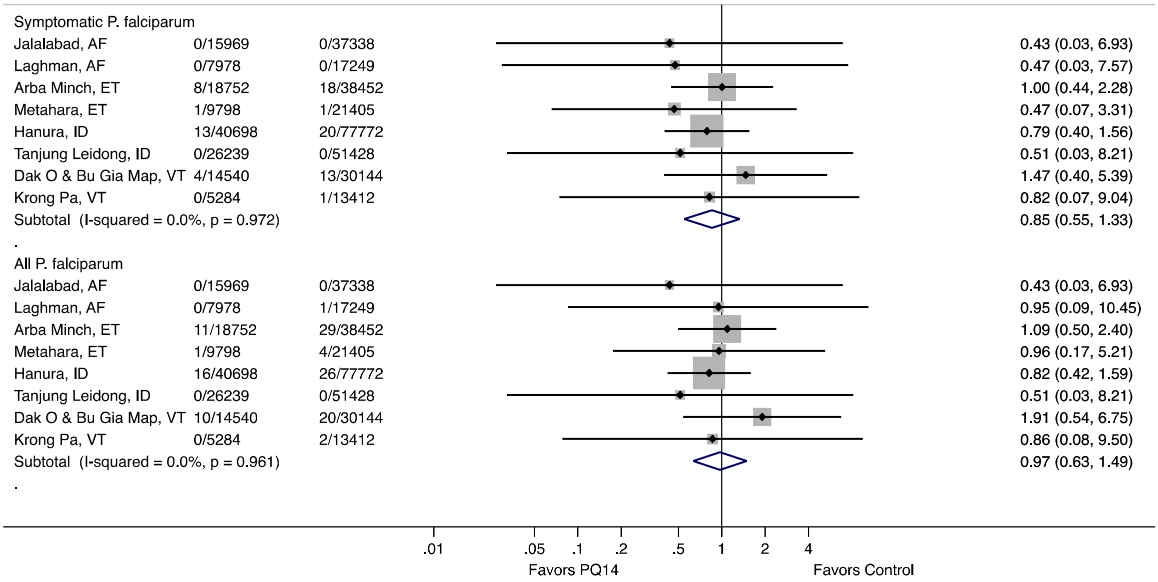

AF = Afghanistan; ET = Ethiopia; ID = Indonesia; VT = Vietnam

i) PQ14 vs Control: Risks and rates for recurrent parasitemia due to symptomatic and any infection due all species

Site	Control arm: events/follow-up time (days)	PQ14 arm: events/follow-up time (days)	Hazard Ratio (95% CI)
Symptomatic P. spp			
Jalalabad, AF	17/6354	9/17404	0.21 (0.10, 0.46)
Laghman, AF	14/3327	16/11030	0.36 (0.18, 0.72)
Arba Minch, ET	34/7937	17/29170	0.15 (0.08, 0.27)
Metahara, ET	16/5485	4/19584	0.09 (0.03, 0.24)
Hanura, ID	46/26756	37/68589	0.32 (0.21, 0.49)
Tanjung Leidong, ID	19/20151	4/45318	0.11 (0.04, 0.30)
Dak O & Bu Gia Map, VT	21/5079	9/20581	0.11 (0.05, 0.24)
Krong Pa, VT	9/3380	1/12786	0.06 (0.01, 0.29)
Subtotal (I-squared = 57.3%, p = 0.022)			0.17 (0.11, 0.26)
.			
All P. spp			
Jalalabad, AF	17/6354	10/17404	0.24 (0.11, 0.50)
Laghman, AF	14/3327	17/11030	0.36 (0.18, 0.72)
Arba Minch, ET	49/7937	30/29170	0.19 (0.12, 0.30)
Metahara, ET	22/5485	8/19584	0.12 (0.06, 0.27)
Hanura, ID	68/26756	48/68589	0.28 (0.19, 0.41)
Tanjung Leidong, ID	21/20151	4/45318	0.10 (0.04, 0.27)
Dak O & Bu Gia Map, VT	32/5079	12/20581	0.10 (0.05, 0.19)
Krong Pa, VT	9/3380	2/12786	0.10 (0.03, 0.36)
Subtotal (I-squared = 53.5%, p = 0.035)			0.18 (0.13, 0.26)
.			

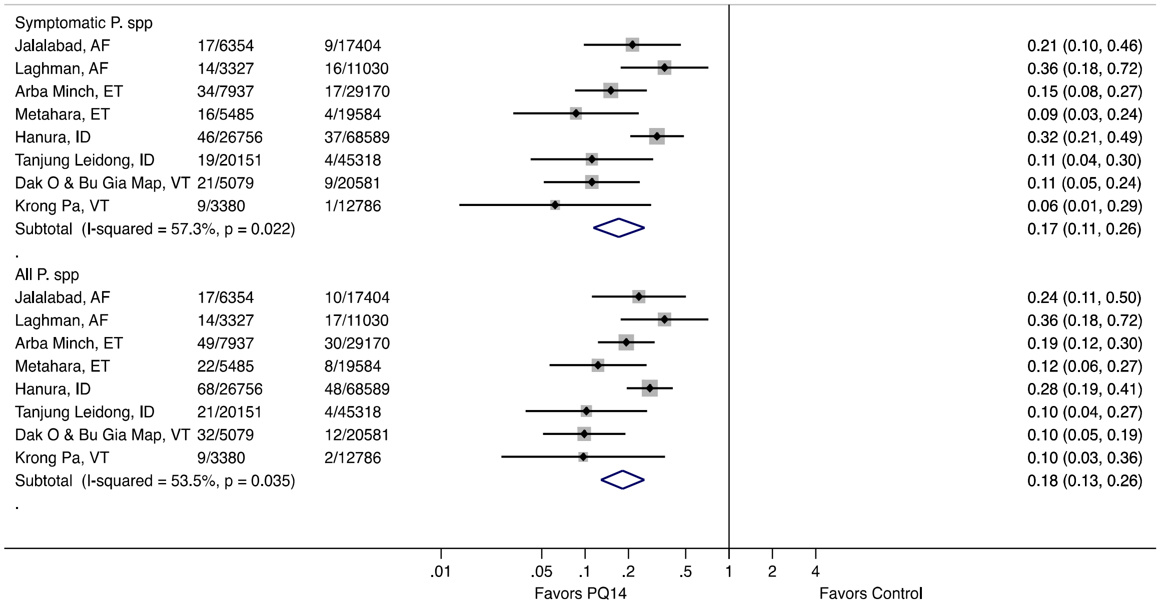

Site	Control: events/follow-up time (days)	PQ14 arm: events/follow-up time (days)	Incidence Rate Ratio (95% CI)
Symptomatic P. spp			
Jalalabad, AF	28/15969	13/37338	0.21 (0.11, 0.39)
Laghman, AF	28/7978	21/17249	0.36 (0.20, 0.63)
Arba Minch, ET	107/18752	49/38452	0.22 (0.15, 0.33)
Metahara, ET	33/9798	5/21405	0.08 (0.03, 0.20)
Hanura, ID	95/40698	50/77772	0.28 (0.19, 0.41)
Tanjung Leidong, ID	28/26239	6/51428	0.12 (0.05, 0.30)
Dak O & Bu Gia Map, VT	52/14540	28/30144	0.24 (0.13, 0.43)
Krong Pa, VT	20/5284	3/13412	0.08 (0.02, 0.31)
Subtotal (I-squared = 47.7%, p = 0.063)			0.21 (0.15, 0.28)
.			
All P. spp			
Jalalabad, AF	28/15969	14/37338	0.22 (0.12, 0.41)
Laghman, AF	30/7978	23/17249	0.36 (0.21, 0.63)
Arba Minch, ET	144/18752	68/38452	0.21 (0.14, 0.32)
Metahara, ET	45/9798	11/21405	0.11 (0.05, 0.24)
Hanura, ID	145/40698	69/77772	0.26 (0.18, 0.36)
Tanjung Leidong, ID	30/26239	6/51428	0.13 (0.05, 0.30)
Dak O & Bu Gia Map, VT	100/14540	38/30144	0.17 (0.10, 0.30)
Krong Pa, VT	20/5284	4/13412	0.09 (0.02, 0.32)
Subtotal (I-squared = 38.2%, p = 0.125)			0.21 (0.16, 0.27)
.			

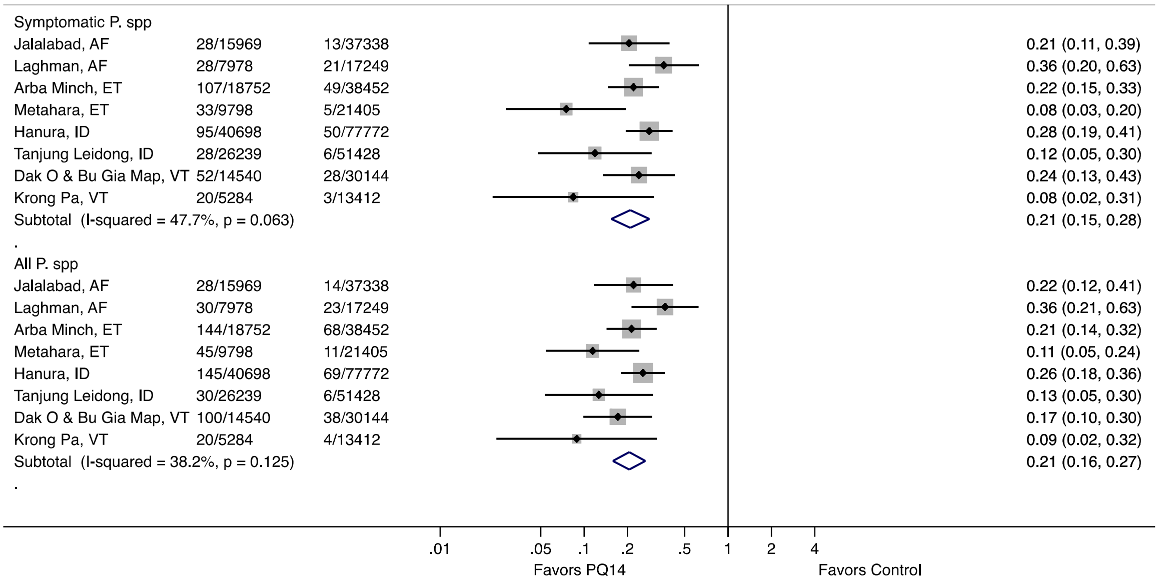

AF = Afghanistan; ET = Ethiopia; ID = Indonesia; VT = Vietnam

19. Site-specific Kaplan Meier Curves for Symptomatic *P. vivax* Recurrence

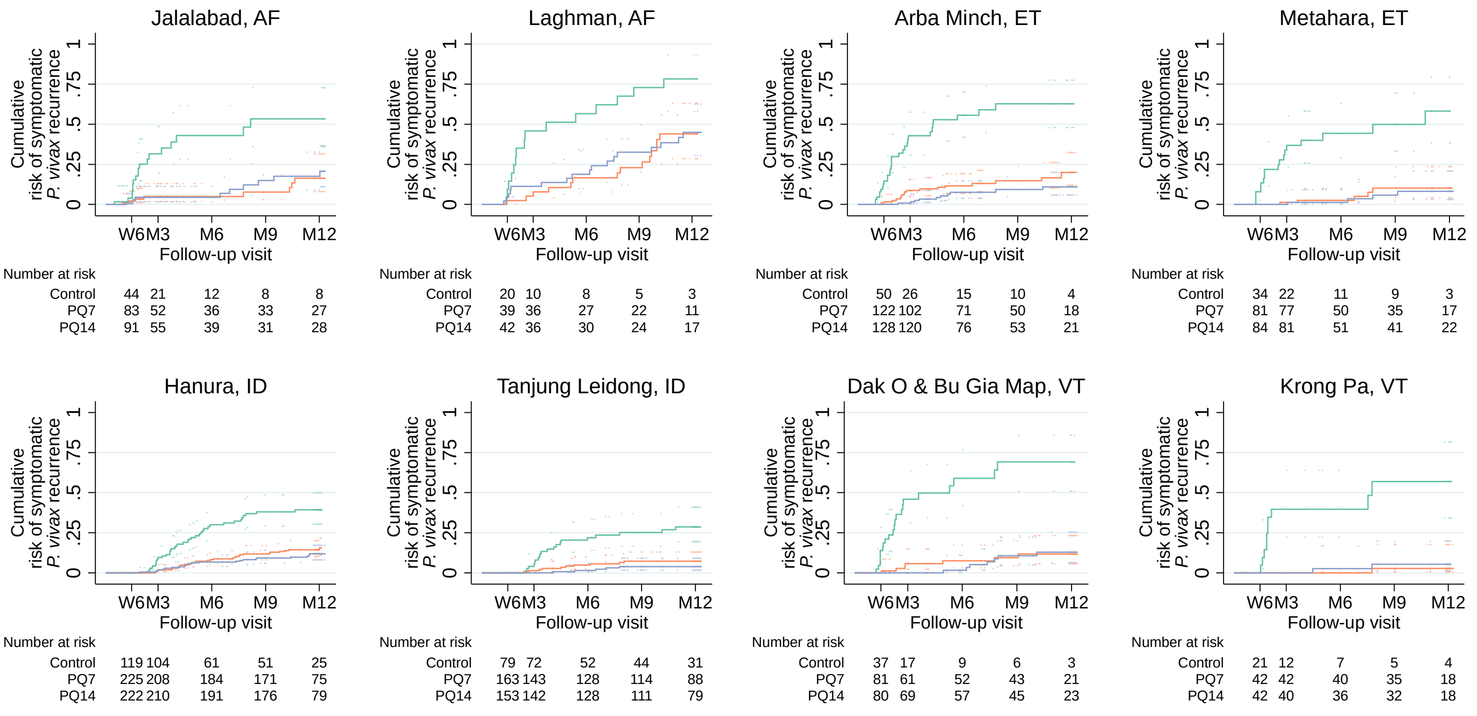

Legend: W=week, M=month

AF = Afghanistan; ET = Ethiopia; ID = Indonesia; VT = Vietnam

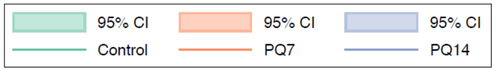

20. Details of SAEs

No	Age (yr.)	Sex	Site	Event/ diagnosis	Criteria for SAE reporting	G6PD status	Day of onset after last treatment	Duration (days)	Relation to study drug	Intensity	Outcome	Action taken to study drug
**PQ7 Treatment Arm**												
1	9	F	ET001	Severe *P. falciparum* malaria	Hospitalization	Normal	46	1	Not related	Severe	Recovered	NA
2	2	M	ET001	Severe *P. falciparum* malaria	Hospitalization	Normal	221	3	Not related	Severe	Recovered	NA
3	2	F	ET001	Severe *P. falciparum* malaria	Hospitalization	Normal	57	2	Not related	Severe	Recovered	NA
4	11	F	ET002	Persistent vomiting	Hospitalization	Normal	10	4	Not related	Severe	Recovered	Temporarily discontinued for 4 days
5	25	F	ET002	Severe malaria (*P. falciparum* +*P. vivax*)	Hospitalization	Normal	2	5	Not related	Severe	Recovered	Continued
6	15	F	ET002	Persistent vomiting	Hospitalization	Normal	4	6	Probably related	Severe	Recovered	Temporarily discontinued for 3 days
7	13	F	ET002	Persistent vomiting	Hospitalization	Normal	2	4	Possibly related	Severe	Recovered	Temporarily discontinued for 6 days
8	25	M	ID004	Abdominal pain, fever of unexplained origin	Hospitalization	Normal	6	3	Not related	Severe	Recovered	Temporarily discontinued for 5 days
9	11	F	ID004	Haemolysis (Hb drop from 11.6 to 6.9 g/dl)	Hb fall <7g/dL	Normal	3	3	Possibly related	Moderate	Recovered	Temporarily discontinued for 1 day

10	23	F	ID004	Fever, abdominal pain, dyspnoea	Hospitalization	Normal	9	5	Possibly related	Severe	Recovered	Temporarily discontinued for 7 days
11	66	M	ID004	Sudden unexpected death due to myocardial infarction	Death	Normal	151	1	Not related	Life-threatening	Fatal	NA
12	11	F	ID004	Symptomatic methaemoglobinaemia & bronchopneumonia	Hospitalization	Normal	10	7	Definitely related	Severe	Recovered	Temporarily discontinued for 2 days. 4 days later permanently discontinued
13	16	F	ID004	Acute appendicitis	Hospitalization	Normal	170	7	Not related	Moderate	Recovered	NA
14	13	M	ID004	Unilateral periorbital ecchymosis	Hospitalization	Normal	54	1	Not related	Moderate	Recovered	NA
15	19	F	ID004	Acute haemolysis (Hb drop from 13.7 to 9.5 g/dl)	Other important medical event	Normal	3	1	Definitely related	Severe	Recovered	Permanently discontinued
16	9	F	ID005	Epigastric pain	Hospitalization	Normal	4	1	Possibly related	Moderate	Recovered	Temporarily discontinued for 3 days
17	7	F	VN001	Diarrhoea	Hospitalization	Normal	7	4	Definitely related	Moderate	Recovered	Continued
18	20	M	VN001	Acute haemolysis (Hb drop from 15.3 to 6.4 g/dl)	Hospitalization and blood transfusion	Deficient*	5	6	Definitely related	Moderate	Recovered	Permanently discontinued
**PQ14 Treatment Arm**												
19	3	M	ET002	Haemolysis (Hb drop from 10.2 to 6.8/dl)	Other important medical event	Normal	3	3	Probably related	Severe	Recovered	Permanently Discontinued
20	11	M	ET002	Acute exacerbation of bronchial asthma precipitated by pneumonia	Hospitalization	Normal	91	5	Not related	Severe	Recovered	NA

21	13	M	ID004	Complicated puncture wound	Hospitalization	Normal	129	5	Not related	Pot. life-threatening	Recovered	NA
22	46	M	ID004	Generalised peritonitis	Hospitalization	Normal	92	13	Not related	Severe	Recovered	NA
23	19	M	VN001	Undifferentiated carcinoma in nasopharynx	Life-threatening	Normal	147		Not related	Moderate	Improving	NA
**Control Arm**												
24	19	F	ET001	Soft tissue injury (car accident)	Hospitalization	Normal	5	3	Not related	Moderate	Recovered	Cont.
25	7	F	ID004	Bacterial enteritis	Hospitalization	Normal	12	4	Not related	Moderate	Recovered	Temporarily discontinued for 9 days
26	9	M	ID004	Dengue fever	Hospitalization	Normal	4	3	Not related	Moderate	Recovered	Temporarily discontinued for 3 days
27	22	M	VN001	Severe *P. falciparum* malaria	Hospitalization	Normal	14	3	Not related	Severe	Recovered	NA
*Patient was wrongly enrolled into G6PD normal arms												

21. Site Specific Adverse Events

	Site	Outcome measure	Control	PQ7	PQ14
**Afghanistan**	**AF001 Jalalabad**	Number enrolled	60	125	126
		SAE PQ related - All severities	0 (0.0%)	0 (0.0%)	0 (0.0%)
		SAE PQ unrelated - All severities	0 (0.0%)	0 (0.0%)	0 (0.0%)
		AE - Grade 3 & 4	0 (0.0%)	1 (0.8%)	0 (0.0%)
		Event duration (days), median (IQR)		5.0 (5.0-5.0)	
		Treatment discontinued N (%)	0 (0.0%)	0 (0.0%)	0 (0.0%)
		Vomited within 1hr of a study dose [initial episode]	1 (1.7%)	1 (0.8%)	1 (0.8%)
	**AF008 Laghman**	Number enrolled	23	48	49
		SAE PQ related - All severities	0 (0.0%)	0 (0.0%)	0 (0.0%)
		SAE PQ unrelated - All severities	0 (0.0%)	0 (0.0%)	0 (0.0%)
		AE - Grade 3 & 4	0 (0.0%)	0 (0.0%)	0 (0.0%)
		Event duration (days), median (IQR)			
		Treatment discontinued N (%)	0 (0.0%)	0 (0.0%)	0 (0.0%)
		Vomited within 1hr of a study dose [initial episode]	0 (0.0%)	0 (0.0%)	0 (0.0%)
**Ethiopia**	**ET001 Arba Minch**	Number enrolled	74	149	148
		SAE PQ related - All severities	0 (0.0%)	0 (0.0%)	0 (0.0%)
		SAE PQ unrelated - All severities	1 (1.4%)	3 (2.0%)	0 (0.0%)
		AE - Grade 3 & 4	0 (0.0%)	0 (0.0%)	0 (0.0%)
		Event duration (days), median (IQR)	3.0 (3.0-3.0)		
		Treatment discontinued N (%)	0 (0.0%)	0 (0.0%)	0 (0.0%)

		Vomited within 1hr of a study dose [initial episode]	4 (5.4%)	2 (1.3%)	4 (2.7%)
	**ET002 Metahara**	Number enrolled	40	85	84
		SAE PQ related - All severities	0 (0.0%)	2 (2.4%)	1 (1.2%)
		SAE PQ unrelated - All severities	0 (0.0%)	2 (2.4%)	1 (1.2%)
		AE - Grade 3 & 4	0 (0.0%)	1 (1.2%)	0 (0.0%)
		Event duration (days), median (IQR)		4.0 (3.0-5.0)	32.0 (32.0-32.0)
		Treatment discontinued N (%)	0 (0.0%)	4 (4.7%)	1 (1.2%)
		Vomited within 1hr of a study dose [initial episode]	0 (0.0%)	1 (1.2%)	0 (0.0%)
**Indonesia**	**ID004 Hanura**	Number enrolled	118	229	228
		SAE PQ related - All severities	0 (0.0%)	4 (1.7%)	0 (0.0%)
		SAE PQ unrelated - All severities	2 (1.6%)	4 (1.7%)	2 (0.8%)
		AE - Grade 3 & 4	0 (0.0%)	4 (1.7%)	1 (0.4%)
		Event duration (days), median (IQR)	3.0 (3.0-3.0)	5.0 (3.0-6.0)	1.0 (1.0-1.0)
		Treatment discontinued N (%)	2 (1.7%)	7 (3.1%)	2 (0.9%)
		Vomited within 1hr of a study dose [initial episode]	5 (4.2%)	11 (4.8%)	10 (4.4%)
	**ID005 Tanjung Leidong**	Number enrolled	85	171	169
		SAE PQ related - All severities	0 (0.0%)	1 (0.6%)	0 (0.0%)
		SAE PQ unrelated - All severities	0 (0.0%)	0 (0.0%)	0 (0.0%)
		AE - Grade 3 & 4	0 (0.0%)	0 (0.0%)	0 (0.0%)
		Event duration (days), median (IQR)		1.0 (1.0-1.0)	
		Treatment discontinued N (%)	1 (1.2%)	1 (0.6%)	2 (1.2%)
		Vomited within 1hr of a study dose [initial episode]	1 (1.2%)	4 (2.3%)	3 (1.8%)
**Vietnam**	**VN001**	Number enrolled	43	87	89

	**Dak O & Bu Gia Map**	SAE PQ related - All severities	0 (0.0%)	2 (2.3%)	0 (0.0%)
		SAE PQ unrelated - All severities	1 (2.3%)	0 (0.0%)	1 (1.1%)
		AE - Grade 3 & 4	0 (0.0%)	1 (1.1%)	0 (0.0%)
		Event duration (days), median (IQR)		4.0 (1.0-6.0)	
		Treatment discontinued N (%)	1 (2.3%)	6 (6.9%)	3 (3.4%)
		Vomited within 1hr of a study dose [initial episode]	0 (0.0%)	2 (2.3%)	1 (1.1%)
	**VN002 Krong Pa**	Number enrolled	21	41	44
		SAE PQ related - All severities	0 (0.0%)	0 (0.0%)	0 (0.0%)
		SAE PQ unrelated - All severities	0 (0.0%)	0 (0.0%)	0 (0.0%)
		AE - Grade 3 & 4	0 (0.0%)	0 (0.0%)	0 (0.0%)
		Event duration (days), median (IQR)			
		Treatment discontinued N (%)	0 (0.0%)	1 (2.4%)	1 (2.3%)
		Vomited within 1hr of a study dose [initial episode]	0 (0.0%)	0 (0.0%)	1 (2.3%)

22. Details of AEs

No	Age (yr.)	Sex	Site	Event/ Diagnosis	Day of onset after last treatment	Duration (days)	Relation to study drug	Grade
**PQ7 Treatment Arm**								
1	38	Female	ID004	Dyspepsia	2	6	Possibly related	3
2	4	Male	AF001	Pneumonia	2	5	Possibly related	3
3	31	Male	VN001	Vomiting & Watery diarrhoea	4	1	Probably related	3
4	13	Female	ID004	Dyspepsia	5	6	Probably related	3
5	13	Female	ID004	Dyspepsia	5	4	Definitely related	3
6	13	Male	ID004	Dyspepsia	5	6	Probably related	3
7	15	Male	ET002	Gastritis	8	2	Possibly related	3
**PQ14 Treatment Arm**								
1	40	Female	ID004	Fever & cold symptoms	10	1	Possibly related	3

23. Symptoms Elicited from Daily Questionnaires During Treatment between Days 1 and 3

The proportion of patients reporting each symptom at least once between days 1 and 3

	Site	Outcome measure	Control	PQ7	PQ14
**Overall**		Number [1]	N=461	N=927	N=934
		Vomiting	55 (11.9%)	137 (14.7%)	110 (11.7%)
		Headache	206 (44.4%)	424 (45.3%)	417 (44.5%)
		Nausea	144 (31.0%)	308 (32.9%)	292 (31.2%)
		Diarrhoea	15 (3.2%)	40 (4.3%)	18 (1.9%)
		Skin Rash	7 (1.5%)	12 (1.3%)	14 (1.5%)
		Poor appetite	166 (35.8%)	352 (37.6%)	347 (37.0%)
		Abdominal Pain	119 (25.6%)	259 (27.7%)	235 (25.1%)
		Myalgia / Arthralgia	112 (24.1%)	219 (23.4%)	205 (21.9%)
		Fever	121 (26.1%)	241 (25.8%)	260 (27.7%)
		Passing dark urine	17 (3.7%)	35 (3.7%)	38 (4.1%)
		Dizziness	66 (14.2%)	137 (14.7%)	133 (14.2%)
		Shortness of Breath	13 (2.8%)	19 (2.0%)	15 (1.6%)
		Itching	9 (1.9%)	16 (1.7%)	18 (1.9%)
		Any GI symptoms [2]	224 (48.3%)	461 (49.3%)	456 (48.7%)
**Afghanistan**	**AF001 Jalalabad**	Number [1]	N=59	N=124	N=125
		Vomiting	1 (1.7%)	5 (4.0%)	6 (4.8%)
		Headache	13 (22.0%)	18 (14.5%)	28 (22.4%)
		Nausea	4 (6.8%)	11 (8.9%)	10 (8.0%)
		Diarrhoea	0 (0.0%)	5 (4.0%)	3 (2.4%)
		Skin Rash	0 (0.0%)	0 (0.0%)	0 (0.0%)
		Poor appetite	3 (5.1%)	13 (10.5%)	14 (11.2%)
		Abdominal Pain	3 (5.1%)	11 (8.9%)	7 (5.6%)
		Myalgia / Arthralgia	8 (13.6%)	9 (7.3%)	19 (15.2%)
		Fever	6 (10.2%)	13 (10.5%)	16 (12.8%)
		Passing dark urine	0 (0.0%)	4 (3.2%)	2 (1.6%)
		Dizziness	4 (6.8%)	13 (10.5%)	8 (6.4%)
		Shortness of Breath	0 (0.0%)	1 (0.8%)	0 (0.0%)
		Itching	1 (1.7%)	0 (0.0%)	2 (1.6%)
		Any GI symptoms [2]	6 (10.2%)	23 (18.5%)	19 (15.2%)
	**AF008 Laghman**	Number [1]	N=23	N=47	N=49
		Vomiting	0 (0.0%)	2 (4.3%)	1 (2.0%)
		Headache	1 (4.3%)	2 (4.3%)	9 (18.4%)
		Nausea	1 (4.3%)	3 (6.4%)	1 (2.0%)
		Diarrhoea	0 (0.0%)	1 (2.1%)	0 (0.0%)
		Skin Rash	0 (0.0%)	0 (0.0%)	0 (0.0%)

		Poor appetite	0 (0.0%)	4 (8.5%)	7 (14.3%)
		Abdominal Pain	0 (0.0%)	2 (4.3%)	2 (4.1%)
		Myalgia / Arthralgia	1 (4.3%)	1 (2.1%)	6 (12.2%)
		Fever	2 (8.7%)	8 (17.0%)	11 (22.4%)
		Passing dark urine	0 (0.0%)	0 (0.0%)	0 (0.0%)
		Dizziness	0 (0.0%)	1 (2.1%)	0 (0.0%)
		Shortness of Breath	0 (0.0%)	0 (0.0%)	0 (0.0%)
		Itching	0 (0.0%)	0 (0.0%)	1 (2.0%)
		Any GI symptoms [2]	1 (4.3%)	5 (10.6%)	8 (16.3%)
**Ethiopia**	**ET001 Arba Minch**	Number [1]	N=72	N=146	N=147
		Vomiting	21 (29.2%)	36 (24.7%)	39 (26.5%)
		Headache	38 (52.8%)	78 (53.4%)	80 (54.4%)
		Nausea	39 (54.2%)	81 (55.5%)	74 (50.3%)
		Diarrhoea	8 (11.1%)	18 (12.3%)	12 (8.2%)
		Skin Rash	5 (6.9%)	10 (6.8%)	11 (7.5%)
		Poor appetite	66 (91.7%)	132 (90.4%)	127 (86.4%)
		Abdominal Pain	35 (48.6%)	60 (41.1%)	61 (41.5%)
		Myalgia / Arthralgia	26 (36.1%)	47 (32.2%)	34 (23.1%)
		Fever	37 (51.4%)	73 (50.0%)	78 (53.1%)
		Passing dark urine	15 (20.8%)	29 (19.9%)	33 (22.4%)
		Dizziness	41 (56.9%)	80 (54.8%)	74 (50.3%)
		Shortness of Breath	11 (15.3%)	17 (11.6%)	15 (10.2%)
		Itching	8 (11.1%)	15 (10.3%)	11 (7.5%)
		Any GI symptoms [2]	68 (94.4%)	136 (93.2%)	135 (91.8%)
	**ET002 Metahara**	Number [1]	N=40	N=84	N=84
		Vomiting	1 (2.5%)	7 (8.3%)	5 (6.0%)
		Headache	14 (35.0%)	29 (34.5%)	30 (35.7%)
		Nausea	4 (10.0%)	11 (13.1%)	11 (13.1%)
		Diarrhoea	0 (0.0%)	1 (1.2%)	1 (1.2%)
		Skin Rash	0 (0.0%)	0 (0.0%)	1 (1.2%)
		Poor appetite	8 (20.0%)	14 (16.7%)	21 (25.0%)
		Abdominal Pain	2 (5.0%)	7 (8.3%)	5 (6.0%)
		Myalgia / Arthralgia	1 (2.5%)	8 (9.5%)	8 (9.5%)
		Fever	11 (27.5%)	19 (22.6%)	26 (31.0%)
		Passing dark urine	0 (0.0%)	0 (0.0%)	0 (0.0%)
		Dizziness	2 (5.0%)	0 (0.0%)	1 (1.2%)
		Shortness of Breath	1 (2.5%)	0 (0.0%)	0 (0.0%)
		Itching	0 (0.0%)	1 (1.2%)	1 (1.2%)
		Any GI symptoms [2]	11 (27.5%)	26 (31.0%)	29 (34.5%)
**Indonesia**	**ID004 Hanura**	Number [1]	N=118	N=228	N=228
		Vomiting	16 (13.6%)	41 (18.0%)	30 (13.2%)
		Headache	56 (47.5%)	125 (54.8%)	112 (49.1%)
		Nausea	40 (33.9%)	92 (40.4%)	82 (36.0%)

		Diarrhoea	4 (3.4%)	9 (3.9%)	2 (0.9%)
		Skin Rash	2 (1.7%)	1 (0.4%)	0 (0.0%)
		Poor appetite	35 (29.7%)	83 (36.4%)	73 (32.0%)
		Abdominal Pain	27 (22.9%)	65 (28.5%)	57 (25.0%)
		Myalgia / Arthralgia	29 (24.6%)	63 (27.6%)	60 (26.3%)
		Fever	39 (33.1%)	73 (32.0%)	76 (33.3%)
		Passing dark urine	2 (1.7%)	1 (0.4%)	3 (1.3%)
		Dizziness	14 (11.9%)	31 (13.6%)	36 (15.8%)
		Shortness of Breath	1 (0.8%)	1 (0.4%)	0 (0.0%)
		Itching	0 (0.0%)	0 (0.0%)	0 (0.0%)
		Any GI symptoms [2]	68 (57.6%)	137 (60.1%)	132 (57.9%)
	**ID005 Tanjung Leidong**	Number [1]	N=85	N=170	N=169
		Vomiting	15 (17.6%)	43 (25.3%)	28 (16.6%)
		Headache	70 (82.4%)	137 (80.6%)	134 (79.3%)
		Nausea	56 (65.9%)	110 (64.7%)	114 (67.5%)
		Diarrhoea	2 (2.4%)	4 (2.4%)	0 (0.0%)
		Skin Rash	0 (0.0%)	0 (0.0%)	0 (0.0%)
		Poor appetite	53 (62.4%)	105 (61.8%)	103 (60.9%)
		Abdominal Pain	51 (60.0%)	110 (64.7%)	101 (59.8%)
		Myalgia / Arthralgia	47 (55.3%)	89 (52.4%)	78 (46.2%)
		Fever	6 (7.1%)	7 (4.1%)	9 (5.3%)
		Passing dark urine	0 (0.0%)	0 (0.0%)	0 (0.0%)
		Dizziness	5 (5.9%)	11 (6.5%)	14 (8.3%)
		Shortness of Breath	0 (0.0%)	0 (0.0%)	0 (0.0%)
		Itching	0 (0.0%)	0 (0.0%)	1 (0.6%)
		Any GI symptoms [2]	67 (78.8%)	125 (73.5%)	128 (75.7%)
**Vietnam**	**VN001 Dak O & Bu Gia Map**	Number [1]	N=43	N=87	N=89
		Vomiting	1 (2.3%)	2 (2.3%)	1 (1.1%)
		Headache	7 (16.3%)	14 (16.1%)	10 (11.2%)
		Nausea	0 (0.0%)	0 (0.0%)	0 (0.0%)
		Diarrhoea	1 (2.3%)	2 (2.3%)	0 (0.0%)
		Skin Rash	0 (0.0%)	1 (1.1%)	2 (2.2%)
		Poor appetite	1 (2.3%)	1 (1.1%)	2 (2.2%)
		Abdominal Pain	1 (2.3%)	3 (3.4%)	2 (2.2%)
		Myalgia / Arthralgia	0 (0.0%)	0 (0.0%)	0 (0.0%)
		Fever	7 (16.3%)	17 (19.5%)	17 (19.1%)
		Passing dark urine	0 (0.0%)	0 (0.0%)	0 (0.0%)
		Dizziness	0 (0.0%)	1 (1.1%)	0 (0.0%)
		Shortness of Breath	0 (0.0%)	0 (0.0%)	0 (0.0%)
		Itching	0 (0.0%)	0 (0.0%)	2 (2.2%)
		Any GI symptoms [2]	3 (7.0%)	7 (8.0%)	5 (5.6%)
	**VN002 Krong Pa**	Number [1]	N=21	N=41	N=43
		Vomiting	0 (0.0%)	1 (2.4%)	0 (0.0%)

		Headache	7 (33.3%)	21 (51.2%)	14 (32.6%)
		Nausea	0 (0.0%)	0 (0.0%)	0 (0.0%)
		Diarrhoea	0 (0.0%)	0 (0.0%)	0 (0.0%)
		Skin Rash	0 (0.0%)	0 (0.0%)	0 (0.0%)
		Poor appetite	0 (0.0%)	0 (0.0%)	0 (0.0%)
		Abdominal Pain	0 (0.0%)	1 (2.4%)	0 (0.0%)
		Myalgia / Arthralgia	0 (0.0%)	2 (4.9%)	0 (0.0%)
		Fever	13 (61.9%)	31 (75.6%)	27 (62.8%)
		Passing dark urine	0 (0.0%)	1 (2.4%)	0 (0.0%)
		Dizziness	0 (0.0%)	0 (0.0%)	0 (0.0%)
		Shortness of Breath	0 (0.0%)	0 (0.0%)	0 (0.0%)
		Itching	0 (0.0%)	0 (0.0%)	0 (0.0%)
		Any GI symptoms [2]	0 (0.0%)	2 (4.9%)	0 (0.0%)

[1]: Number of patients with daily symptoms questionnaires during treatment
[2]: Composite of any of the following: nausea, vomiting, anorexia, diarrhoea or abdominal pain

24. Symptoms elicited from daily questionnaires during treatment – Days 4 to 14

The proportion of patients reporting each symptom at least once between days 4 and 14

	Site	Outcome measure	Control	PQ7	PQ14
**Overall**		Number [1]	N=456	N=914	N=930
		Vomiting	14 (3.0%)	72 (7.7%)	47 (5.0%)
		Headache	93 (20.0%)	236 (25.2%)	201 (21.5%)
		Nausea	41 (8.8%)	143 (15.3%)	105 (11.2%)
		Diarrhoea	11 (2.4%)	67 (7.2%)	39 (4.2%)
		Skin Rash	8 (1.7%)	15 (1.6%)	16 (1.7%)
		Poor appetite	53 (11.4%)	179 (19.1%)	129 (13.8%)
		Abdominal Pain	45 (9.7%)	282 (30.2%)	172 (18.4%)
		Myalgia / Arthralgia	23 (5.0%)	71 (7.6%)	60 (6.4%)
		Fever	36 (7.8%)	80 (8.6%)	93 (9.9%)
		Passing dark urine	7 (1.5%)	27 (2.9%)	18 (1.9%)
		Dizziness	33 (7.1%)	77 (8.2%)	56 (6.0%)
		Shortness of Breath	6 (1.3%)	17 (1.8%)	14 (1.5%)
		Itching	8 (1.7%)	12 (1.3%)	9 (1.0%)
		Any GI symptoms [2]	92 (19.8%)	372 (39.8%)	259 (27.6%)
**Afghanistan**	**AF001 Jalalabad**	Number [1]	N=57	N=116	N=123
		Vomiting	0 (0.0%)	7 (6.0%)	4 (3.3%)
		Headache	3 (5.3%)	12 (10.3%)	5 (4.1%)
		Nausea	0 (0.0%)	12 (10.3%)	3 (2.4%)
		Diarrhoea	0 (0.0%)	5 (4.3%)	2 (1.6%)
		Skin Rash	0 (0.0%)	0 (0.0%)	0 (0.0%)
		Poor appetite	1 (1.8%)	14 (12.1%)	3 (2.4%)
		Abdominal Pain	0 (0.0%)	18 (15.5%)	8 (6.5%)
		Myalgia / Arthralgia	2 (3.5%)	5 (4.3%)	3 (2.4%)
		Fever	3 (5.3%)	1 (0.9%)	3 (2.4%)
		Passing dark urine	0 (0.0%)	3 (2.6%)	1 (0.8%)
		Dizziness	1 (1.8%)	8 (6.9%)	4 (3.3%)
		Shortness of Breath	0 (0.0%)	2 (1.7%)	0 (0.0%)
		Itching	0 (0.0%)	0 (0.0%)	0 (0.0%)
		Any GI symptoms [2]	1 (1.8%)	23 (19.8%)	11 (8.9%)
	**AF008 Laghman**	Number [1]	N=23	N=46	N=49
		Vomiting	0 (0.0%)	2 (4.3%)	1 (2.0%)
		Headache	0 (0.0%)	0 (0.0%)	1 (2.0%)
		Nausea	0 (0.0%)	1 (2.2%)	1 (2.0%)
		Diarrhoea	0 (0.0%)	2 (4.3%)	1 (2.0%)
		Skin Rash	0 (0.0%)	0 (0.0%)	0 (0.0%)

		Poor appetite	0 (0.0%)	2 (4.3%)	1 (2.0%)
		Abdominal Pain	0 (0.0%)	3 (6.5%)	2 (4.1%)
		Myalgia / Arthralgia	0 (0.0%)	0 (0.0%)	1 (2.0%)
		Fever	0 (0.0%)	1 (2.2%)	1 (2.0%)
		Passing dark urine	0 (0.0%)	0 (0.0%)	0 (0.0%)
		Dizziness	0 (0.0%)	0 (0.0%)	0 (0.0%)
		Shortness of Breath	0 (0.0%)	0 (0.0%)	0 (0.0%)
		Itching	0 (0.0%)	0 (0.0%)	0 (0.0%)
		Any GI symptoms [2]	0 (0.0%)	4 (8.7%)	3 (6.1%)
**Ethiopia**	**ET001 Arba Minch**	Number [1]	N=71	N=143	N=146
		Vomiting	9 (12.7%)	23 (16.1%)	14 (9.6%)
		Headache	16 (22.5%)	40 (28.0%)	36 (24.7%)
		Nausea	16 (22.5%)	39 (27.3%)	28 (19.2%)
		Diarrhoea	7 (9.9%)	27 (18.9%)	22 (15.1%)
		Skin Rash	5 (7.0%)	12 (8.4%)	10 (6.8%)
		Poor appetite	35 (49.3%)	69 (48.3%)	59 (40.4%)
		Abdominal Pain	17 (23.9%)	62 (43.4%)	49 (33.6%)
		Myalgia / Arthralgia	8 (11.3%)	19 (13.3%)	14 (9.6%)
		Fever	6 (8.5%)	16 (11.2%)	16 (11.0%)
		Passing dark urine	4 (5.6%)	16 (11.2%)	14 (9.6%)
		Dizziness	22 (31.0%)	37 (25.9%)	26 (17.8%)
		Shortness of Breath	3 (4.2%)	5 (3.5%)	3 (2.1%)
		Itching	5 (7.0%)	11 (7.7%)	6 (4.1%)
		Any GI symptoms [2]	43 (60.6%)	93 (65.0%)	90 (61.6%)
	**ET002 Metahara**	Number [1]	N=39	N=84	N=84
		Vomiting	0 (0.0%)	8 (9.5%)	0 (0.0%)
		Headache	6 (15.4%)	12 (14.3%)	7 (8.3%)
		Nausea	0 (0.0%)	6 (7.1%)	0 (0.0%)
		Diarrhoea	0 (0.0%)	2 (2.4%)	2 (2.4%)
		Skin Rash	0 (0.0%)	1 (1.2%)	0 (0.0%)
		Poor appetite	1 (2.6%)	10 (11.9%)	1 (1.2%)
		Abdominal Pain	0 (0.0%)	13 (15.5%)	4 (4.8%)
		Myalgia / Arthralgia	0 (0.0%)	3 (3.6%)	1 (1.2%)
		Fever	2 (5.1%)	1 (1.2%)	2 (2.4%)
		Passing dark urine	0 (0.0%)	0 (0.0%)	0 (0.0%)
		Dizziness	0 (0.0%)	0 (0.0%)	1 (1.2%)
		Shortness of Breath	0 (0.0%)	0 (0.0%)	1 (1.2%)
		Itching	0 (0.0%)	0 (0.0%)	0 (0.0%)
		Any GI symptoms [2]	1 (2.6%)	21 (25.0%)	6 (7.1%)
**Indonesia**	**ID004 Hanura**	Number [1]	N=118	N=228	N=228
		Vomiting	4 (3.4%)	11 (4.8%)	21 (9.2%)
		Headache	36 (30.5%)	82 (36.0%)	80 (35.1%)
		Nausea	15 (12.7%)	35 (15.4%)	43 (18.9%)

		Diarrhoea	3 (2.5%)	10 (4.4%)	9 (3.9%)
		Skin Rash	3 (2.5%)	0 (0.0%)	5 (2.2%)
		Poor appetite	10 (8.5%)	44 (19.3%)	40 (17.5%)
		Abdominal Pain	18 (15.3%)	99 (43.4%)	61 (26.8%)
		Myalgia / Arthralgia	9 (7.6%)	20 (8.8%)	29 (12.7%)
		Fever	17 (14.4%)	41 (18.0%)	50 (21.9%)
		Passing dark urine	3 (2.5%)	7 (3.1%)	2 (0.9%)
		Dizziness	8 (6.8%)	21 (9.2%)	19 (8.3%)
		Shortness of Breath	3 (2.5%)	10 (4.4%)	10 (4.4%)
		Itching	3 (2.5%)	0 (0.0%)	1 (0.4%)
		Any GI symptoms [2]	30 (25.4%)	121 (53.1%)	90 (39.5%)
	**ID005 Tanjung Leidong**	Number [1]	N=84	N=170	N=169
		Vomiting	1 (1.2%)	12 (7.1%)	6 (3.6%)
		Headache	32 (38.1%)	89 (52.4%)	71 (42.0%)
		Nausea	10 (11.9%)	44 (25.9%)	30 (17.8%)
		Diarrhoea	1 (1.2%)	8 (4.7%)	1 (0.6%)
		Skin Rash	0 (0.0%)	0 (0.0%)	0 (0.0%)
		Poor appetite	6 (7.1%)	32 (18.8%)	25 (14.8%)
		Abdominal Pain	10 (11.9%)	61 (35.9%)	41 (24.3%)
		Myalgia / Arthralgia	4 (4.8%)	24 (14.1%)	12 (7.1%)
		Fever	6 (7.1%)	11 (6.5%)	14 (8.3%)
		Passing dark urine	0 (0.0%)	0 (0.0%)	1 (0.6%)
		Dizziness	2 (2.4%)	11 (6.5%)	6 (3.6%)
		Shortness of Breath	0 (0.0%)	0 (0.0%)	0 (0.0%)
		Itching	0 (0.0%)	0 (0.0%)	1 (0.6%)
		Any GI symptoms [2]	17 (20.2%)	78 (45.9%)	51 (30.2%)
**Vietnam**	**VN001 Dak O & Bu Gia Map**	Number [1]	N=43	N=86	N=89
		Vomiting	0 (0.0%)	7 (8.1%)	1 (1.1%)
		Headache	0 (0.0%)	1 (1.2%)	1 (1.1%)
		Nausea	0 (0.0%)	5 (5.8%)	0 (0.0%)
		Diarrhoea	0 (0.0%)	11 (12.8%)	2 (2.2%)
		Skin Rash	0 (0.0%)	2 (2.3%)	1 (1.1%)
		Poor appetite	0 (0.0%)	5 (5.8%)	0 (0.0%)
		Abdominal Pain	0 (0.0%)	22 (25.6%)	6 (6.7%)
		Myalgia / Arthralgia	0 (0.0%)	0 (0.0%)	0 (0.0%)
		Fever	2 (4.7%)	7 (8.1%)	7 (7.9%)
		Passing dark urine	0 (0.0%)	1 (1.2%)	0 (0.0%)
		Dizziness	0 (0.0%)	0 (0.0%)	0 (0.0%)
		Shortness of Breath	0 (0.0%)	0 (0.0%)	0 (0.0%)
		Itching	0 (0.0%)	1 (1.2%)	1 (1.1%)
		Any GI symptoms [2]	0 (0.0%)	28 (32.6%)	7 (7.9%)
	**VN002 Krong Pa**	Number [1]	N=21	N=41	N=42
		Vomiting	0 (0.0%)	2 (4.9%)	0 (0.0%)

		Headache	0 (0.0%)	0 (0.0%)	0 (0.0%)
		Nausea	0 (0.0%)	1 (2.4%)	0 (0.0%)
		Diarrhoea	0 (0.0%)	2 (4.9%)	0 (0.0%)
		Skin Rash	0 (0.0%)	0 (0.0%)	0 (0.0%)
		Poor appetite	0 (0.0%)	3 (7.3%)	0 (0.0%)
		Abdominal Pain	0 (0.0%)	4 (9.8%)	1 (2.4%)
		Myalgia / Arthralgia	0 (0.0%)	0 (0.0%)	0 (0.0%)
		Fever	0 (0.0%)	2 (4.9%)	0 (0.0%)
		Passing dark urine	0 (0.0%)	0 (0.0%)	0 (0.0%)
		Dizziness	0 (0.0%)	0 (0.0%)	0 (0.0%)
		Shortness of Breath	0 (0.0%)	0 (0.0%)	0 (0.0%)
		Itching	0 (0.0%)	0 (0.0%)	0 (0.0%)
		Any GI symptoms [2]	0 (0.0%)	4 (9.8%)	1 (2.4%)

[1]: Number of patients with daily symptoms questionnaires during treatment
[2]: Composite of any of the following: nausea, vomiting, anorexia, diarrhoea or abdominal pain

25. Distribution of haemoglobin (g/dL) during follow up by treatment arm

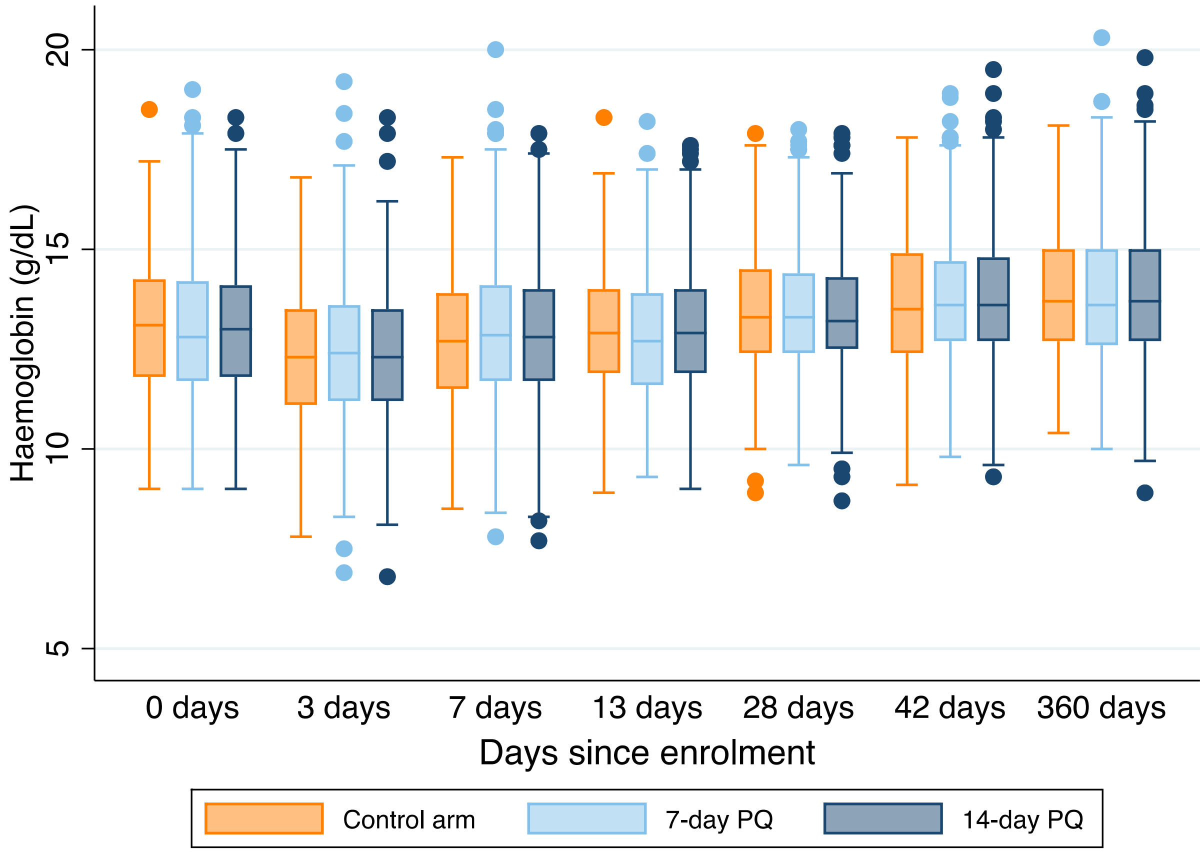

Boxes represent 25th and 75th percentiles.

26. Haemoglobin Profile by Treatment Arm

	Control	PQ7	PQ14
Number of patients	**464**	**935**	**937**
Incidence risk of severe anaemia (<7 g/dL) or transfusion within 365 days (95% CI) [N]	[N = 0]	0.40 (0.13, 1.30) [N = 3]	0.11 (0.02, 0.77) [N = 1]
Hb drop >5g/dL within 7 days of initial treatment N (%)	0 (0.00)	0 (0.00)	1 (0.00)
Hb nadir within 28 days of treatment initiation, g/dL , mean[SD]	11.96 [1.6]	11.92 [1.6]	11.86 [1.6]
Time to Hb nadir, days, median (range)	3 (3 - 7)	3 (3 - 13)	3 (3 - 13)
**Day 0**			
Number of Patients	464	934	937
Hb on day 0, g/dL, mean[SD]	13.04 [1.7]	12.96 [1.8]	12.94 [1.7]
**Day 3**			
Number of Patients	432	891	895
Hb on day 3, g/dL , mean[SD]	12.31 [1.7]	12.41 [1.7]	12.30 [1.7]
Change in Hb between Day 0 and Day 3, g/dL, mean[SD]	-0.72 [1.12]	-0.52 [1.19]	-0.62 [1.09]
Absolute drop between Day 0 and Day 3 >5 g/dL, N (%)	0 (0.00)	0 (0.00)	1 (0.11)
Fractional change in Hb between Day 0 and Day 3, %, mean[SD]	-5.26 [8.64]	-3.60 [9.17]	-4.53 [8.47]
Fractional drop between Day 0 and Day 3 >25%, N (%)	3 (0.69)	8 (0.89)	8 (0.89)
**Day 7**			
Number of patients	447	882	888
Hb on day 7, g/dL , mean[SD]	12.72 [1.7]	12.91 [1.8]	12.81 [1.7]
Change in Hb between Day 0 and Day 7, g/dL, mean[SD]	-0.31 [1.20]	-0.01 [1.23]	-0.12 [1.09]
Absolute drop between Day 0 and Day 7 >5 g/dL, N (%)	0 (0.00)	0 (0.00)	0 (0.00)
Fractional change in Hb between Day 0 and Day 7, % , mean[SD]	-2.08 [9.01]	0.32 [9.58]	-0.62 [8.72]
Fractional drop between Day 0 and Day 7 >25%, N (%)	2 (0.45)	9 (1.02)	2 (0.23)
**Day 13**			
Number of patients	438	852	866
Hb on day 13, g/dL , mean[SD]	12.97 [1.6]	12.82 [1.6]	12.98 [1.5]
Change in Hb between Day 0 and Day 13, g/dL, mean[SD]	-0.07 [1.23]	-0.10 [1.26]	0.07 [1.15]
Absolute drop between Day 0 and Day 13 >5 g/dL, N (%)	0 (0.00)	1 (0.12)	0 (0.00)

Fractional change in Hb between Day 0 and Day 13, %, mean[SD]	-0.02 [9.48]	-0.08 [9.85]	1.08 [9.30]
Fractional drop between Day 0 and Day 13 >25%, N (%)	0 (0.00)	3 (0.35)	2 (0.23)
**Day 28**			
Number of patients	396	817	813
Hb on day 28, g/dL , mean[SD]	13.43 [1.6]	13.47 [1.5]	13.38 [1.5]
**Day 42**			
Number of patients	377	785	773
Hb on day 42, g/dL , mean[SD]	13.63 [1.7]	13.75 [1.5]	13.76 [1.6]
